# Data-driven approach to elucidate the correlation between photocatalytic activity and rate constants from excited states

**DOI:** 10.1039/d5sc06465a

**Published:** 2025-11-25

**Authors:** Ryuga Kunisada, Manami Hayashi, Tabea Rohlfs, Taiki Nagano, Koki Sano, Naoto Inai, Naoki Noto, Takuya Ogaki, Yasunori Matsui, Hiroshi Ikeda, Olga García Mancheño, Takeshi Yanai, Susumu Saito

**Affiliations:** a Graduate School of Science, Nagoya University Nagoya Aichi 464-8602 Japan yanai.takeshi.e4@f.mail.nagoya-u.ac.jp saito.susumu.c4@f.mail.nagoya-u.ac.jp; b Organic Chemistry Institute, University of Münster Münster 48149 Germany; c Integrated Research Consortium on Chemical Sciences (IRCCS), Nagoya University Nagoya Aichi 464-8602 Japan noto.naoki.f5@f.mail.nagoya-u.ac.jp; d Graduate School of Engineering, Osaka Metropolitan University, Sakai Osaka 599-8531 Japan; e Institute of Transformative Bio-Molecules (WPI-ITbM), Nagoya University Nagoya Aichi 464-8602 Japan

## Abstract

Even though excited-state properties play a crucial role in photocatalysis, directly correlating these with photocatalytic activity remains challenging. Herein, we propose a method to elucidate the correlations between the catalytic activity of organic photosensitizers and the rate constants of various excited-state processes through integrating machine learning (ML), quantum chemical calculations, and chemical experiments. This approach was applied to interpolative predictions of the yield of the nickel/photocatalytic formation of C–O bonds and radical additions to alkenes using various organic photosensitizers with satisfactory accuracy (*R*^2^ = 0.83 and 0.77 on the test set, respectively). The calculated rate constants obtained through quantum chemical calculations proved to be comparable or even superior to the experimentally measured excited-state lifetimes as descriptors. SHAP-based visual analysis revealed that the rate constants corresponding to transitions from the T_1_ state provide significant contributions to the interpolative prediction of photocatalytic activity. Additionally, the non-radiative decay process between the S_1_ and S_0_ states helps describe the low catalytic activity of poorly emissive photosensitizers. These findings highlight the potential of the proposed method to provide insights into photocatalytic properties that are difficult to obtain using conventional approaches.

## Abstract 2

A data-driven framework integrating machine learning and quantum chemical calculations enables elucidation of how rate constants from excited states govern the photocatalytic activity of organic photosensitizers.

## Graphical abstract

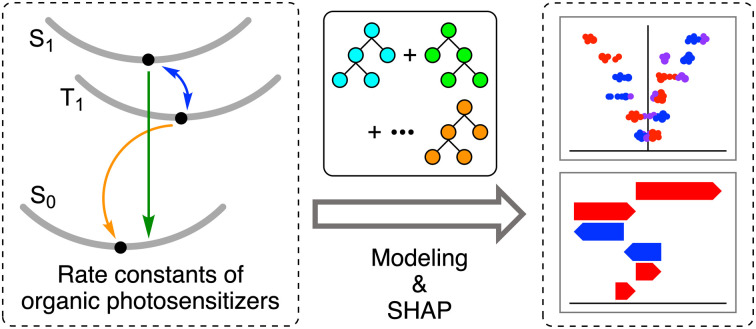

## Introduction

The excitation of organic molecules induces intriguing phenomena, including photocatalysis.^1–9^ However, elucidating the relationship between excited-state properties and the resulting photochemical behavior is highly challenging using conventional experimental approaches.

The excited-state lifetimes of photosensitizers are considered to play a crucial role in facilitating energy-transfer and electron-transfer processes with substrates, although a long excited-state lifetime does not necessarily correlate with a higher product yield.^10^ Therefore, clarifying the relationship between photocatalytic activity and excited-state lifetimes, or related properties such as decay rate constants, is essential to understand the behavior of organic photosensitizers (OPSs), which exhibit diverse excited-state properties. Recent advances in quantum chemical calculations have made the prediction of excited-state characteristics, *e.g.*, first-principles prediction of rate constants for various processes in the excited state, more accessible.^11–21^ However, to elucidate how the complex excited-state properties influence photocatalytic activity using quantum chemical calculations, further integration with robust tools to decipher these relationships is required.

Machine learning (ML) is increasingly being applied across diverse chemical fields, including organic synthesis.^22–27^ A common application of ML in this field is the prediction of the product yield and selectivity in order to identify the optimal catalyst and reaction conditions.^28–34^ In addition to its predictive capabilities, ML is valuable for uncovering correlations between inputs, *e.g.*, the properties of substrates and catalysts, and outputs, *e.g.*, the product yield and selectivity of reactions.^35–37^ Shapley additive explanations (SHAP), a tool based on game theory that has been developed to enhance the interpretability of ML models, is highly useful in this context.^38–44^ SHAP enables the quantitative assessment of how individual descriptors contribute to the trends in the overall predicted outputs and to the predictions for individual samples. However, despite its utility, the application of ML to the characterization of photocatalytic properties remains an area with considerable room for further development.^40,45–54^ In particular, approaches that can correlate essential yet elusive excited-state properties, such as rate constants from excited states, with photocatalytic activity are still underdeveloped.

**Fig. 1:**
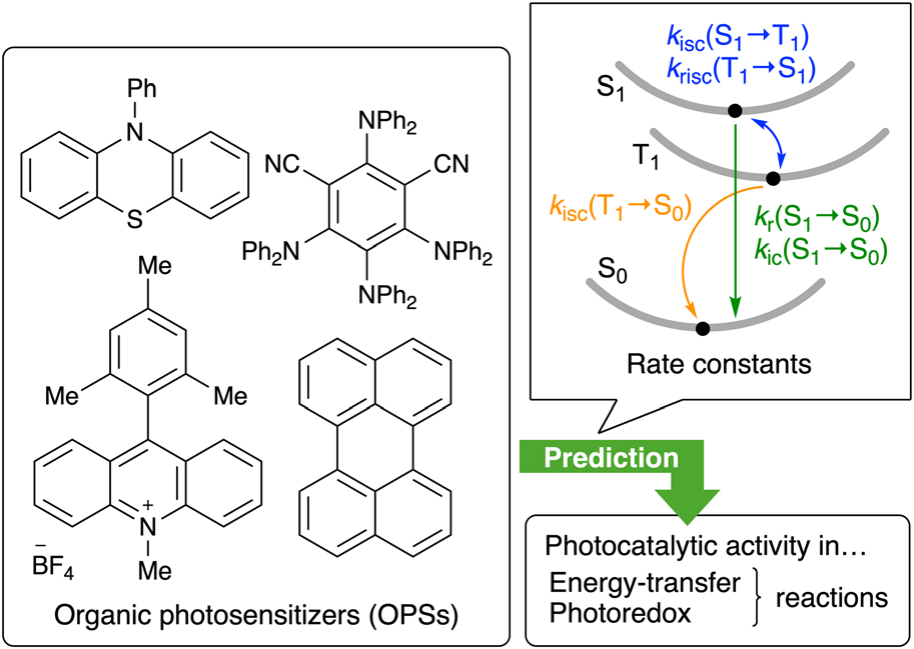
Rate constants obtained from quantum chemical calculations for the prediction of photocatalytic activity.

Here, we propose a data-driven approach to estimate the catalytic activity of OPSs using theoretically simulated rate constants from excited states (Fig. 1), whose effectiveness in ML for photocatalysis remains underexplored. Specifically, rate constants for the radiative (*k*_r_(S_1_ → S_0_)) and non-radiative (*k*_ic_(S_1_ → S_0_)) processes from the S_1_ to the S_0_ state, the intersystem crossing (ISC; *k*_isc_(S_1_ → T_1_)) and reverse ISC (*k*_risc_(T_1_ → S_1_)) processes between the S_1_ and T_1_ states, and the ISC process from the T_1_ state to the S_0_ state (*k*_isc_(T_1_ → S_0_)) were simulated using a combination of time-dependent density functional theory (TD-DFT) and excited-state-dynamics theory based on the thermal vibration correlation function (TVCF). Descriptor sets incorporating these DFT-based properties were used for the ML-based interpolative prediction of the photocatalytic activity in energy-transfer and photoredox reactions, *i.e.*, nickel-catalyzed C–O bond formation and radical addition. Our protocol demonstrates that the integration of advanced quantum chemical calculations into ML represents a pertinent tool to elucidate how complex excited-state characteristics influence photocatalytic activity, thereby highlighting a potential avenue through which data science can contribute to chemical research.

## Results and discussion

### Experimentally measured and theoretically simulated rate constants of representative OPSs

Recently, Inai and Yanai have reported a photochemical study in which a first-principles method based on TD-DFT and TVCF calculations was used to predict the decay rate constants of organic dyes.^55^ We applied this method to simulate the rate constants of various excited-state processes for OPSs. We first selected four compounds (OPS1, OPS5, OPS7, and OPS47) from among the 60 OPSs used in this study and compared their experimentally measured rate constants with the theoretically simulated ones. A complete list of all OPSs is provided in the SI (Fig. S2).

OPS1, commonly known as 4CzIPN, is an organic compound that exhibits thermally activated delayed fluorescence (TADF).^56^OPS5 and OPS7 are derivatives of OPS1 featuring 3,6-dimethoxycarbazolyl and diphenylamino groups, respectively, instead of the carbazolyl groups of OPS1. OPS47 also contains diphenylamino groups as electron-donor moieties but differs in having a nitrophenyl group as an electron acceptor. The photophysical properties of OPS1 in toluene, including fluorescence lifetimes (*τ*) and quantum yield (*Φ*), have already been reported.^56–60^ Methoxy-substituted OPS5 exhibits shorter *τ* values and a much lower *Φ* than OPS1, while OPS7 has a lower *Φ* value together with a significantly extended *τ*. OPS47 has a very low *Φ* with very short *τ* values and does not exhibit TADF properties. All these *τ* and *Φ* values are summarized in Table 1.

**Table 1:** Excited-state properties of OPS1, OPS5, OPS7, and OPS47^a^

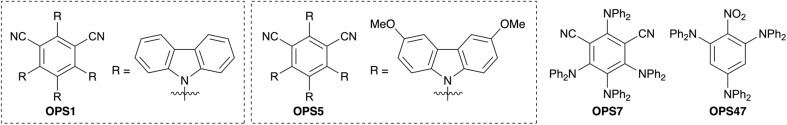					
OPS	τ (*Φ*)^b^	Δ*E*_ST_/eV	*k*_r_(S_1_ → S_0_)/s^−1^	*k*_isc_(S_1_ → T_1_)/s^−1^	*k*_risc_(T_1_ → S_1_)/s^−1^^c^
OPS1	14.2 ns, 1.8 µs^c^ (0.94)^d^	0.08^d^^,^^e^ (0.37)^f^	1.7 × 10^7^^c^^,^^e^ (2.0 × 10^7^)^f^	5.1 × 10^7^^c^^,^^e^ (2.6 × 10^7^^g^, 1.1 × 10^7^^h^)	2.7 × 10^6^^c^^,^^e^ (5.3 × 10^g^, 2.7 × 10^6^^h^)
OPS5	4.8 ns, 0.6 µs (0.06)	0.03^e^ (0.39)^f^	1.1 × 10^7^^e^ (7.8 × 10^6^)^f^	2.0 × 10^8^^e^ (4.1 × 10^6^^g^, 2.5 × 10^8^^h^)	2.9 × 10^5^^e^ (2.6 × 10^−1^^g^, 1.6 × 10^7^^h^)
OPS7	1.7 ns, 34.4 µs (0.20)	0.12^e^ (0.70)^f^	4.8 × 10^7^^e^ (2.4 × 10^7^)^f^	5.5 × 10^8^^e^ (3.3 × 10^5^^g^, 5.4 × 10^8^^h^)	4.7 × 10^4^^e^ (6.8 × 10^−5^^g^, 3.7 × 10^5^^h^)
OPS47	3.5 ns, 9.9 ns (0.02)	0.15^e^ (0.42)^f^	5.7 × 10^6^^e^ (1.9 × 10^6^)^f^	2.1 × 10^8^^e^ (1.7 × 10^8^^g^, 3.8 × 10^6^^h^)	6.2 × 10^5^^e^ (6.0 × 10^2^^g^, 6.9 × 10^5^^h^)

a For details of how these properties were obtained, see the SI.

b Luminescence lifetimes (*τ*) and quantum yields (*Φ*) were measured in toluene under an argon atmosphere.

c The excited-state lifetimes and rate constants of OPS1 were obtained from ref. 57.

d The *Φ* and Δ*E*_ST_ values of OPS1 were obtained from ref. 56 and 58–60.

e Experimentally determined values are presented.

f Theoretically simulated values are presented.

g Theoretically simulated *k*_isc_(S_1_ → T_1_) and *k*_risc_(T_1_ → S_1_) values, which were calculated using computational (DFT-based) Δ*E*_ST_ values, are presented.

h Theoretically simulated *k*_isc_(S_1_ → T_1_) and *k*_risc_(T_1_ → S_1_) values, which were calculated using experimental (measurement-based) Δ*E*_ST_ values, are presented.

The rate constants of these OPSs were experimentally determined (Table 1). Detailed procedures for estimating the rate constants are described in the SI (Experimentally determined rate constants section). The experimentally measured rate constants of OPS1 have also been reported previously.^57^OPS5 and OPS7 exhibit higher *k*_isc_(S_1_ → T_1_) values than OPS1, whereas their *k*_risc_(T_1_ → S_1_) values are lower. OPS47 shows lower *k*_r_(S_1_ → S_0_) and higher *k*_isc_(S_1_ → T_1_) values than OPS1, which might explain its poor luminescence properties.

The theoretically simulated rate constants for these OPSs are also summarized in Table 1. The calculations were conducted using the TD-DFT and TVCF methods described in the previous study.^55^ The underlying DFT and TD-DFT calculations were carried out at the PCM(toluene)-CAM-B3LYP/6-31G(d) level, and the theoretically simulated *k*_r_(S_1_ → S_0_) values show good agreement with the experimentally determined values for all four OPSs.

To calculate *k*_isc_(S_1_ → T_1_) and *k*_risc_(T_1_ → S_1_), the adiabatic singlet–triplet splitting (Δ*E*_ST_) values of the OPSs are required. Specifically, the TVCF method sums over vibronic levels under the harmonic approximation and employs Δ*E*_ST_ as the detuning parameter in the phase factor rather than a standalone proxy for the activation. Considering that accurately computing Δ*E*_ST_ values *via* DFT-based methods remains challenging,^61–63^ we compared the effects of using the computational (DFT-based) or experimental (measurement-based) Δ*E*_ST_ values on the resulting *k*_isc_(S_1_ → T_1_) and *k*_risc_(T_1_ → S_1_) values. The Δ*E*_ST_ values of OPS1, OPS5, OPS7, and OPS47 obtained *via* quantum chemical calculations (OPS1: 0.37 eV; OPS5: 0.39 eV; OPS7: 0.70 eV; OPS47: 0.42 eV) and experiments (OPS1: 0.08 eV; OPS5: 0.03 eV; OPS7: 0.12 eV; OPS47: 0.15 eV) are provided in Table 1. In addition, theoretically simulated *k*_isc_(S_1_ → T_1_) and *k*_risc_(T_1_ → S_1_) values that were refined using the experimental Δ*E*_ST_ are also listed in Table 1.

The relative effectiveness of using the computational or experimental Δ*E*_ST_ value for the calculation of the *k*_isc_(S_1_ → T_1_) value varied on a case-by-case basis. Both methods provided similar *k*_isc_(S_1_ → T_1_) values for OPS1 (computational Δ*E*_ST_: 2.6 × 10^7^ s^−1^; experimental Δ*E*_ST_: 1.1 × 10^7^ s^−1^). The computational Δ*E*_ST_ values resulted in better agreement with the experimentally determined *k*_isc_(S_1_ → T_1_) for OPS47 (computational Δ*E*_ST_: 1.7 × 10^8^ s^−1^; experimental Δ*E*_ST_: 3.8 × 10^6^ s^−1^), whereas the opposite effect was observed for OPS5 (computational Δ*E*_ST_: 4.1 × 10^6^ s^−1^; experimental Δ*E*_ST_: 2.5 × 10^8^ s^−1^) and OPS7 (computational Δ*E*_ST_: 3.3 × 10^5^ s^−1^; experimental Δ*E*_ST_: 5.4 × 10^8^ s^−1^).

In contrast, the experimental Δ*E*_ST_ values were clearly more effective for simulating *k*_risc_(T_1_ → S_1_). The fully computation-based approach significantly underestimated the *k*_risc_(T_1_ → S_1_) values (OPS1: 5.3 × 10 s^−1^; OPS5: 2.6 × 10^−1^ s^−1^; OPS7: 6.8 × 10^−5^ s^−1^; OPS47: 6.0 × 10^2^ s^−1^) compared to the cases where the experimental Δ*E*_ST_ values were used (OPS1: 2.7 × 10^6^ s^−1^; OPS5: 1.6 × 10^7^ s^−1^; OPS7: 3.7 × 10^5^ s^−1^; OPS47: 6.9 × 10^5^ s^−1^). However, despite this underestimation, the former method captured more accurately the relative magnitudes of OPS1 and OPS5 than the latter, indicating that the use of fully computational values in ML is not always inferior.

For processes involving ISC, using the experimental Δ*E*_ST_ value for the TVCF calculations tended to provide values closer to the experimentally determined rate constants, due to the difficulty of accurately estimating Δ*E*_ST_*via* DFT-based approaches. Meanwhile, for compounds with poor emission properties, experimentally determining the Δ*E*_ST_ value is difficult. In addition, a fully computational approach is more promising in terms of ensuring the future applicability of this framework to compounds that have not yet been synthesized. Alternatively, the prediction accuracy of Δ*E*_ST_ can be significantly improved using higher-level wavefunction-based methods such as SCS-CC2.^62,63^ However, the computational cost of such methods still remains prohibitively high for the relatively large molecular datasets used for ML. Therefore, we chose to use the Δ*E*_ST_ values obtained from DFT-level calculations, *i.e.*, the computational Δ*E*_ST_ values, in the subsequent ML investigations.

### Comparison of the model performance in C–O bond-forming reactions

**Fig. 2:**
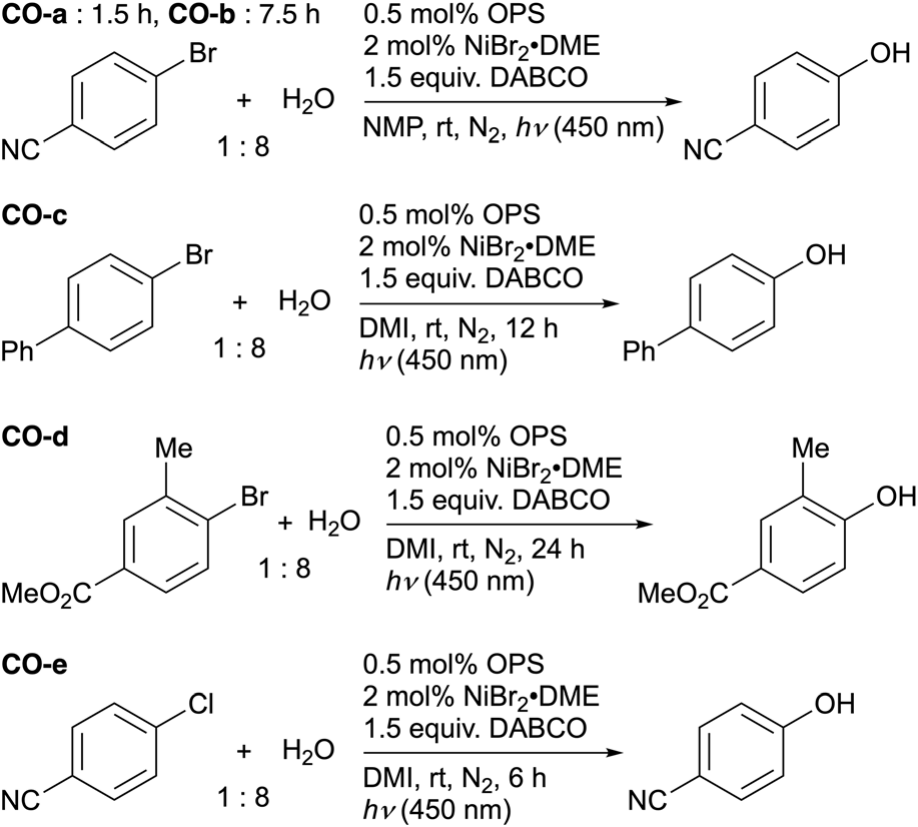
C–O bond-forming reactions tested in this study.

Although the focus of the present study was on quantifying the relative contributions of the model inputs (OPS properties) to the model output (predicted yield) rather than achieving maximum model accuracy, a certain level of model performance is crucial for meaningful feature interpretation. In our case, the challenge lies in attaining reasonable model performance while using only descriptors that are meaningful from a photochemical perspective. Therefore, we first investigated the impact of the employed descriptors on the accuracy of interpolative predictions for C–O bond-forming reactions using various aryl halides,^64–66^ which are considered to be promoted mainly by energy transfer (Fig. 2).^50,67–69^ Our dataset included reactions using four different aryl halides, *i.e.*, 4-bromobenzonitrile (CO-a, CO-b), 4-bromobiphenyl (CO-c), methyl 4-bromo-3-methylbenzoate (CO-d), and 4-chlorobenzonitrile (CO-e). For 4-bromobenzonitrile, data for two distinct reaction times (1.5 h: CO-a; 7.5 h: CO-b) were considered. In our previous study, 60 OPSs were tested for each C–O bond-forming reaction,^54^ resulting in a dataset consisting of 300 data points. The differences among the OPSs were represented by their DFT-derived properties, and the reaction types (*i.e.*, CO-a–CO-e) were encoded using one-hot encoding. In all the ML investigations, the target variable was the product yield. Since the yield was determined using the employed OPS and the reaction type, the developed models can be regarded as ML models for the estimation of the photocatalytic activity in similar reactions. Other details of the ML setup are summarized in Table 2.

**Table 2:** Model performance for the interpolative prediction of the photocatalytic activity in C–O bond-forming reactions^a^

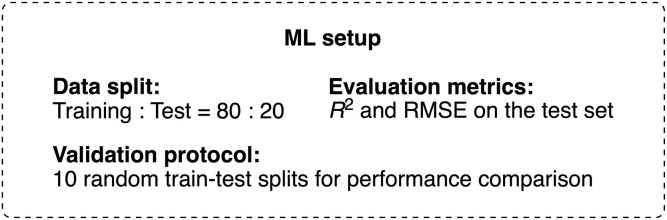			
Entry	Descriptor set	*R* ^2^	RMSE
1	RC	0.79 (0.05)	13.1 (1.2)
2	s_RC	0.78 (0.04)	13.5 (1.1)
3	s_RC + ‘*E*_HOMO_, *f*_S1_, Δ*E*_ST_, ΔDM’	0.83 (0.04)	11.8 (1.3)
4	‘*E*_HOMO_, *f*_S1_, Δ*E*_ST_, ΔDM’	0.79 (0.06)	13.0 (1.4)
5	LT_t	0.66 (0.06)	16.9 (1.9)
6	LT_d	0.64 (0.08)	17.2 (1.4)
7	LT_t + ‘*E*_HOMO_, *f*_S1_, Δ*E*_ST_, ΔDM’	0.80 (0.05)	12.8 (1.5)
8	LT_d + ‘*E*_HOMO_, *f*_S1_, Δ*E*_ST_, ΔDM’	0.79 (0.05)	13.1 (1.2)

a *R* ^2^ and RMSE scores on the test set were averaged over 10 runs (standard deviations in parentheses).

In addition to the three theoretically simulated rate constants mentioned earlier, *i.e.*, *k*_r_(S_1_ → S_0_), *k*_isc_(S_1_ → T_1_), and *k*_risc_(T_1_ → S_1_), we also incorporated *k*_ic_(S_1_ → S_0_) and *k*_isc_(T_1_ → S_0_). The adiabatic singlet–triplet splitting between the S_0_ and T_1_ states was required to calculate *k*_isc_(T_1_ → S_0_) instead of that between the S_1_ and T_1_ states, which was used for the estimation of *k*_isc_(S_1_ → T_1_) and *k*_risc_(T_1_ → S_1_). In contrast, the rate constant for the radiative process from the T_1_ state to the S_0_ state was not included because phosphorescence is intrinsically weak in OPSs and its contribution to the overall excited-state kinetics is negligible. These five rate constants were used directly to generate a descriptor set comprising five descriptors (referred to as RC). Alternatively, scaled descriptors expressed as ratios among the five rate constants were used to generate another five-descriptor set (denoted as s_RC). The method used to prepare the s_RC descriptor set is summarized in the SI (Computational details for the design of descriptors section). A preliminary investigation identified histogram-based gradient boosting (HGB) as an effective ML model (Table S13). Among the rate constants calculated at different levels of theory, those derived from the PCM(toluene)-CAM-B3LYP/6-31G(d) level provided the best model performance for both RC and s_RC, although the differences were not significant (Table S15). Therefore, descriptors calculated at this level were used in subsequent investigations. The mean *R*^2^ scores on the test set were 0.79 for RC and 0.78 for s_RC (Table 2, entries 1 and 2), indicating that reasonable interpolative predictions can be achieved using only the rate-constant information.

Subsequently, we examined whether incorporating other physical properties relevant to the photoreactions could lead to more robust models. The additional descriptors include the HOMO (*E*_HOMO_) and LUMO (*E*_LUMO_) energy levels, the vertical-excitation (absorption) energy of the lowest singlet (*E*_S1_) and triplet (*E*_T1_) excited states, the corresponding vertical Δ*E*_ST_, the oscillator strength of the lowest singlet excitation (*f*_S1_), and the differences between the ground- and excited-state dipole moments (ΔDM). These descriptors were calculated at the same PCM(toluene)-CAM-B3LYP/6-31G(d) level. Details of the preparation for these descriptors are provided in the SI (Computational details for the design of descriptors section). We compared the model performance of all 127 combinations of these descriptors in conjunction with RC or s_RC, and identified s_RC, *E*_HOMO_, *f*_S1_, Δ*E*_ST_, and ΔDM as the best descriptor set (entry 3; *R*^2^ = 0.83, RMSE = 11.8). When the rate constants were excluded from the descriptor set, the model performance was lower than the best case (entry 4; *R*^2^ = 0.79, RMSE = 13.0), indicating that combining s_RC with other descriptors leads to improved accuracy.

Furthermore, the effectiveness of using experimentally measured excited-state lifetimes as descriptors was examined. The excited-state lifetimes were measured using transient absorption spectroscopy in toluene or DMSO (referred to as LT_t and LT_d, respectively). When either LT_t or LT_d was used as a standalone descriptor, the model performance significantly decreased (entries 5 and 6; LT_t: *R*^2^ = 0.66, RMSE = 16.9; LT_d: *R*^2^ = 0.64, RMSE = 17.2). Additionally, when either LT_t or LT_d was combined with *E*_HOMO_, *f*_S1_, Δ*E*_ST_, and ΔDM, the resulting scores (entries 7 and 8; *R*^2^ = 0.79–0.80, RMSE = 13.1–12.8) did not surpass those obtained using the calculated rate constants.

One major issue with constructing a database of experimentally measured excited-state properties is the difficulty of performing all measurements under identical conditions. For example, while most of the excited-state lifetimes in this study were measured using toluene or DMSO as the solvent, some data points were obtained in other solvents, such as acetonitrile (OPS59, OPS60) or DMF (OPS44, OPS56, OPS59, OPS60), due to solubility and related issues (Table S12). Additionally, unlike theoretically simulated rate constants, experimentally measured excited-state lifetimes represent a combined value that encompasses various excited-state processes. These inconsistencies in measurement conditions and the limited ability to distinguish individual excited-state processes may have contributed to the decreased accuracy observed when using **LT_t** or **LT_d**. Furthermore, clarifying each individual kinetic parameter requires considerable experimental effort. Therefore, the theoretically simulated rate constants, which can provide more details regarding the molecular excited states, have superior utility as descriptors as well as greater interpretability (*vide infra*).

### SHAP-based analysis for C–O bond-forming reactions

**Fig. 3:**
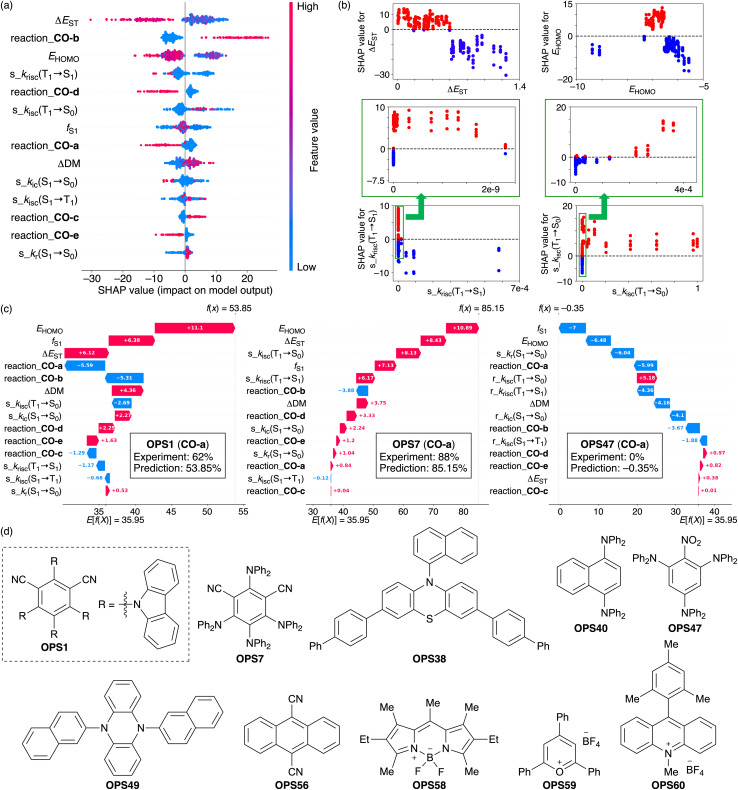
SHAP (a) summary, (b) scatter, and (c) waterfall plots (for CO-a) for the quantification of feature contributions. (d) OPSs discussed in the SHAP-based analysis. Plots including more detailed information are displayed in Fig. S7. The original plots are included in the GitHub repository (https://github.com/Naoki-Noto/P6-20240509-RK).

We next used SHAP to quantify the contributions of descriptors to the interpolative predictions in the case where the best model performance was achieved (all data: *R*^2^ = 0.96; test set: *R*^2^ = 0.88). In this analysis, we generated a SHAP summary plot (Fig. 3a) to capture the overall trends, and SHAP scatter plots (Fig. 3b) to clarify the contribution of each descriptor. In addition, SHAP waterfall plots were used to visually display the feature contributions of individual OPSs for **CO-a** (Fig. 2). In this reaction, the experimental yields for OPS1, OPS7, and OPS47 were 62%, 88%, and 0%, respectively. Briefly, a higher absolute SHAP value indicates a greater contribution of the corresponding descriptor, and its signum shows whether the contribution affects the prediction positively or negatively. Additional explanations on how to interpret these plots are provided in the SI (Description of SHAP plots section). Descriptors prefixed with ‘reaction_’ represent the one-hot encoding used to identify reaction types. Additionally, the term ‘feature’ is used synonymously with ‘descriptor’.

The SHAP summary plot revealed that Δ*E*_ST_ and *E*_HOMO_ are the two most impactful descriptors excluding the one-hot encoding descriptors (Fig. 3a). Highly negative SHAP values for Δ*E*_ST_ were observed for high Δ*E*_ST_ values, whereas lower Δ*E*_ST_ values lead to positive SHAP values (Fig. 3b). A range of moderate *E*_HOMO_ values resulted in highly positive SHAP values, while those outside this range yielded negative SHAP values (Fig. 3b). In the C–O bond-forming reactions, OPSs that meet these conditions to afford positive SHAP values, such as OPS1 and OPS7 (Fig. 3c), generally exhibit better catalytic activity. In contrast, OPSs with high Δ*E*_ST_ values, *e.g.*, OPS56 and OPS58, those with strong reducing capacity, *e.g.*, OPS38, OPS40, and OPS49, and those with strong oxidizing capacity, *e.g.*, OPS59 and OPS60, tend to furnish low product yields (Table S17). This SHAP-based analysis demonstrates the ability to effectively capture the correlations between physical properties and photocatalytic activity.

Next, we analyzed the contributions of the rate constants from the summary plot (Fig. 3a). As mentioned earlier, these descriptors are expressed as ratios of the five processes and are prefixed with ‘s_’. Among the five descriptors derived from rate constants, the descriptors for two processes, *i.e.*, s_*k*_risc_(T_1_ → S_1_) and s_*k*_isc_(T_1_ → S_0_), showed greater contributions than the other three. Given that the triplet state of OPSs is highly likely to be involved in photosensitization,^4,6,70,71^ it is reasonable to assume that descriptors representing transitions from the T_1_ state to other states are important. Although the s_*k*_risc_(T_1_ → S_1_) values tended to be underestimated in the employed descriptor set, the SHAP scatter plots revealed that s_*k*_risc_(T_1_ → S_1_) values within a certain range tend to result in positive SHAP values, whereas those outside this range produce negative SHAP values (Fig. 3b). Similarly, s_*k*_isc_(T_1_ → S_0_) values that fell within a specific range also tend to exhibit positive SHAP values (Fig. 3b). The SHAP waterfall plots for OPS1 and OPS7 (Fig. 3c) indicate that while *E*_HOMO_, *f*_S1_, Δ*E*_ST_, and ΔDM exhibit relatively similar positive SHAP values, s_*k*_risc_(T_1_ → S_1_) and s_*k*_isc_(T_1_ → S_0_) are the primary contributors to distinguishing the catalytic activity of OPS1 and OPS7. The predicted yields were 53.85% for OPS1 and 85.15% for OPS7, demonstrating relatively high accuracy. The SHAP values for s_*k*_risc_(T_1_ → S_1_) were −1.17 for OPS1 and +6.17 for OPS7, while those for s_*k*_isc_(T_1_ → S_0_) were −2.69 for OPS1 and +8.13 for OPS7. Thus, based on SHAP, 18.16% of the 31.30% difference in predicted yields between OPS1 and OPS7 can be attributed to these two descriptors derived from rate constants.

Meanwhile, OPS47 is structurally distinct from OPS7 in terms of its acceptor moiety, resulting in a significantly lower quantum yield (OPS1: *Φ* = 0.94; OPS7: *Φ* = 0.20; OPS47: *Φ* = 0.02) and catalytic activity compared to OPS1 and OPS7. The SHAP waterfall plots revealed that Δ*E*_ST_, *E*_HOMO_, *f*_S1_, and ΔDM, which provided highly positive SHAP values for OPS1 and OPS7, do not contribute positively to the model output for OPS47 (Fig. 3c). Additionally, the negative SHAP value derived from s_*k*_risc_(T_1_ → S_1_) (−4.36) for OPS47 contributes to distinguishing the catalytic activity of OPS7 and OPS47. The s_*k*_r_(S_1_ → S_0_) of OPS47 was 2.7 × 10^−4^ and its s_*k*_ic_(S_1_ → S_0_) was 0.768, indicating that the former is significantly lower and the latter significantly higher compared to those of OPS1 (s_*k*_r_(S_1_ → S_0_): 0.431; s_*k*_ic_(S_1_ → S_0_): 9.6 × 10^−4^) and OPS7 (s_*k*_r_(S_1_ → S_0_): 0.573; s_*k*_ic_(S_1_ → S_0_): 2.4 × 10^−4^). Unlike in the case of OPS1 and OPS7, these descriptors have negative SHAP values of −6.04 and −4.10 for OPS47. The low s_*k*_r_(S_1_ → S_0_) and high s_*k*_ic_(S_1_ → S_0_) observed for OPS47 reflect its poor luminescence properties. The low catalytic activity of the OPSs, for which non-radiative decay pathways unrelated to the transition to the triplet state are favored, is consistent with chemical intuition. The ML-derived outcome, in which the s_*k*_r_(S_1_ → S_0_) and s_*k*_ic_(S_1_ → S_0_) values of OPS47 negatively impact its output, support this notion.

### Application to radical–addition reactions to alkenes

We then extended the scope of our ML strategy to photoredox reactions. As target reactions, we selected the addition of trifluoromethyl, 2,2,2-trifluoroethyl, monofluoromethyl, cyclohexyl, and trifluoromethylthio radicals to 1,1-di(*p*-tolyl)ethylene.^72–78^ In this study, these reactions to synthesize trisubstituted alkenes are referred to as **CF**_**3**_, **CF**_**3**_**CH**_**2**_, **CH**_**2**_**F**, **Cy**, and **SCF**_**3**_, respectively (Table 3). By testing the same database of 60 OPSs for these five photoredox reactions, we constructed a dataset consisting of 300 data points. The HGB-based ML models were constructed using the same setup as that used for the C–O bond-forming reactions (Table 2).

**Table 3:** Model performance for the interpolative prediction of the photocatalytic activity in the radical–addition reactions to 1,1-di(*p*-tolyl)ethylene^a^

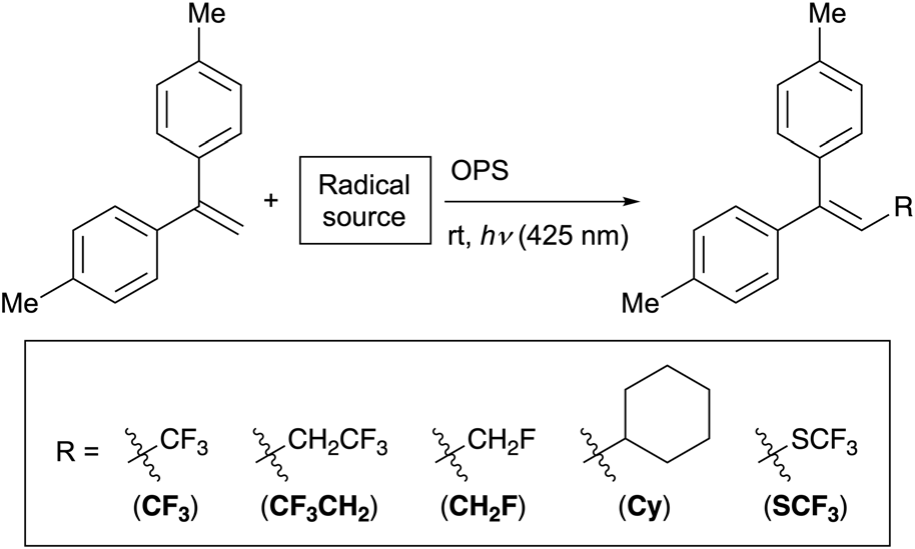			
Entry	Descriptor set	*R* ^2^	RMSE
1	**s_RC**	0.67 (0.05)	19.3 (1.9)
2	**s_RC** + ‘*E*_LUMO_, *f*_S1_, *E*_T1_’	0.77 (0.03)	16.1 (1.5)
3	**LT_t** + ‘*E*_LUMO_, *f*_S1_, *E*_T1_’	0.73 (0.05)	17.5 (1.6)
4	**LT_d** + ‘*E*_LUMO_, *f*_S1_, *E*_T1_’	0.75 (0.03)	16.6 (0.9)

a For details of reaction conditions, see the ESI. *R*^2^ and RMSE scores on the test set were averaged over 10 runs (standard deviations in parentheses).

Since the model performance did not differ significantly among the descriptor sets based on rate constants (Table S16), we continued to use the **s_RC** descriptor set calculated at the PCM(toluene)-CAM-B3LYP/6-31G(d) level in order to maintain consistency with the results of the C–O bond-forming reactions. Although the model performance using **s_RC** alone was relatively poor (Table 3, entry 1; *R*^2^ = 0.67), combining **s_RC** with *E*_LUMO_, *f*_S1_, and *E*_T1_ improved the interpolative predictions, yielding a mean *R*^2^ score of 0.77 and a mean RMSE of 16.1 on the test set (entry 2). While a long excited-state lifetime is known to contribute to efficient electron transfer, it is not always the sole factor determining the product yield, as reported for similar photoredox reactions.^10,79,80^ Thus, it is reasonable that the model based solely on **s_RC** exhibits relatively poor performance, and combining **s_RC** with other descriptors such as *E*_LUMO_ improves the model accuracy. When the experimentally obtained **LT_t** or **LT_d** descriptor set was used instead of **s_RC**, the resulting *R*^2^ scores were 0.73 and 0.75, respectively (Table 3, entries 3 and 4), confirming the satisfactory performance of an entirely DFT-derived descriptor set.

**Fig. 4:**
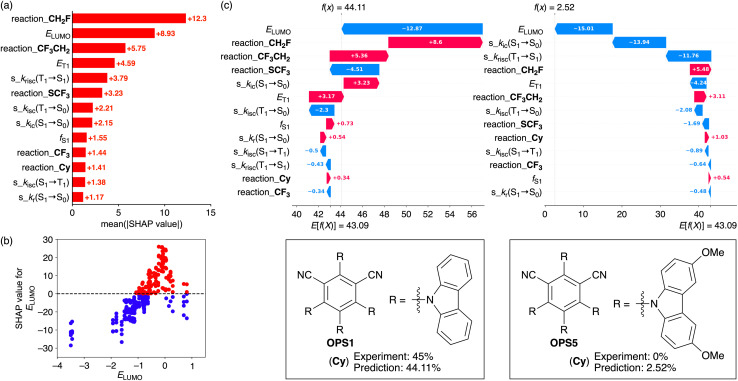
SHAP (a) bar and (b) scatter plots as well as (c) waterfall plots (for **Cy**) for the quantification of feature contributions. Waterfall plots including the more detailed information are displayed in Fig. S8. The original plots are included in the GitHub repository (https://github.com/Naoki-Noto/P6-20240509-RK).

A SHAP-based analysis was subsequently conducted for the best-performing model (all data: *R*^2^ = 0.94; test set: *R*^2^ = 0.82). The SHAP bar plot depicting the mean SHAP values of the descriptors indicated that *E*_LUMO_, which exhibits a high correlation coefficient with *E*_HOMO_ (0.86) and is associated with the redox capacity of the OPSs, shows the largest contribution excluding the one-hot encoding descriptors (Fig. 4a). The SHAP scatter plot (Fig. 4b) revealed that a high *E*_LUMO_ contributes to positive SHAP values, but when the *E*_LUMO_ is too high, the SHAP values shift negatively. Both oxidizing and reducing properties are important in photoredox reactions, and an excessively high *E*_LUMO_ values implies a weak oxidizing capacity for an OPS. Therefore, the SHAP-derived result that an excessively high *E*_LUMO_ negatively influences the product yield appears reasonable. Furthermore, it is known that while the product yield in photoredox reactions is strongly affected by the lifetime of radicals generated *in situ*, an excessively high *E*_LUMO_ can negatively influence the lifetime of radicals derived from OPSs and consequently reduce the product yield.^81,82^ This insight is also consistent with the result obtained from SHAP.

The trends in the feature contribution of rate constants in the ML model for the radical–addition reactions are similar to those observed in the ML model for the C–O bond-forming reactions (Fig. 3a and 4a). Notably, s_*k*_risc_(T_1_ → S_1_) and s_*k*_isc_(T_1_ → S_0_), which are key descriptors for the C–O bond-forming reactions, also play an important role in the ML model for the radical–addition reactions. Next, SHAP waterfall plots were generated for OPS1 and OPS5 in **Cy** (Fig. 4c). In this reaction, OPS1 and OPS5 afforded experimental yields of 45% and 0%, respectively. OPS1 and OPS5 differ in the absence or presence of methoxy groups on their skeletons. The predicted yields for OPS1 and OPS5 were 44.11% and 2.52%, respectively, indicating that the ML model can distinguish the difference in product yields based on their physical properties. The SHAP waterfall plots (Fig. 4c) revealed that, while *E*_LUMO_ exhibits a highly negative SHAP value in both cases (OPS1: −12.87; OPS5: −15.01), *E*_T1_ exhibits significantly different SHAP values (OPS1: +3.17; OPS5: −4.24). The s_*k*_ic_(S_1_ → S_0_) and s_*k*_risc_(T_1_ → S_1_) parameters exhibit significantly more negative SHAP values for OPS5 than OPS1, with larger differences in SHAP values (17.17 and 11.33) than *E*_T1_ (7.41). Thus, based on SHAP, 28.50% of the 41.59% difference in predicted yield can be attributed to s_*k*_ic_(S_1_ → S_0_) and s_*k*_risc_(T_1_ → S_1_).

The higher s_*k*_ic_(S_1_ → S_0_) value of OPS5 (3.4 × 10^−2^) than that of OPS1 (9.6 × 10^−4^) supports its poor luminescence properties. The lower s_*k*_risc_(T_1_ → S_1_) of OPS5 (2.1 × 10^−8^) than that of OPS1 (1.2 × 10^−6^) is consistent with the trend in the experimental values. We have long recognized that the methoxy group is a substituent that specifically impairs luminescence properties and catalytic activity,^50,77^ although explaining the dramatic effects of “MeO” using more fundamental physical properties is challenging. It is noteworthy that incorporating theoretically simulated rate constants into ML enabled us to capture such small yet specific differences. Moreover, as mentioned earlier, our study clarified that when rate constants are generated using the same computational method, their correlation with photocatalytic activity in energy-transfer and photoredox reactions is similar.

## Conclusions

We have developed a data-driven approach to elucidate the correlation between the rate constants from excited states and the catalytic activity of organic photosensitizers (OPSs) by integrating machine learning (ML), quantum chemical calculations, and experiments. The theoretically simulated rate constants provided a model performance that was comparable, or even superior, to that obtained using the experimental excited-state lifetimes. A SHAP-based analysis revealed that the rate constants associated with transitions from the T_1_ state to other states, as well as those from the S_1_ state to the S_0_ state, play a crucial role in determining the photocatalytic activity.

Among the effective OPSs reported so far, there have been cases where S_1_-state contributions were observed,^80,83^ indicating complexity and diversity in their excited-state behavior. Nevertheless, part of the SHAP-derived outcomes demonstrates that, overall, properties associated with transitions from the T_1_ state play an important role in governing the photocatalytic activity. Researchers are often influenced by biases derived from a limited set of experimental observations, particularly from compounds they are most familiar with. Therefore, incorporating statistical, data-driven approaches can provide a more comprehensive and objective perspective for catalyst design and mechanistic understanding.

Beyond its utility for capturing general trends across the dataset, SHAP is particularly useful for case-by-case analyses. Through this framework, we successfully elucidated the correlations between excited-state properties influenced by subtle structural variations and the corresponding photocatalytic activity. For example, differences were observed in s_*k*_isc_(T_1_ → S_0_) and s_*k*_risc_(T_1_ → S_1_) for OPS1 and OPS7 (carbazolyl *vs.* diphenylamino groups), in s_*k*_r_(S_1_ → S_0_) and s_*k*_ic_(S_1_ → S_0_) for OPS7 and OPS47 (nitro *vs.* cyano groups), and in s_*k*_ic_(S_1_ → S_0_) and s_*k*_risc_(T_1_ → S_1_) for OPS1 and OPS5 (the presence or absence of methoxy groups), which are consistent with experimental observations and chemical intuitions. Statistical analyses based on our dataset suggest that these factors account for the observed differences in catalytic activity. In particular, the developed descriptors, *e.g.*, s_*k*_ic_(S_1_ → S_0_), successfully captured the characteristics of OPSs with poor luminescent properties. Such case-specific analyses are compatible with the nature of organic chemistry.

Although the DFT-level computational approach introduces some numerical uncertainty particularly in *k*_isc_(S_1_ → T_1_) and *k*_risc_(T_1_ → S_1_), the resulting relative relationships are sufficient for our ML framework to capture the overall trends, as substantiated by the improved model performance and the agreement between the SHAP-based analysis and experimental interpretations. We show that excited-state properties, which are often difficult to capture experimentally, can be reasonably related to photocatalytic activity at a feasible computational cost. This provides a chemically meaningful contribution beyond purely data-driven aspects. Meanwhile, to further generalize this strategy, continued experimental and computational efforts to construct databases that encompass a broader range of compounds and that incorporate more accurate photophysical properties are essential. For instance, when incorporating complex photosensitizers based on iridium or ruthenium, which are known to be highly effective, we should consider, for example, ultrafast excited-state processes^84^ and the radiative rate constant associated with the T_1_–S_0_ transition, which are requirements that differ significantly from those of OPSs. Developing rational strategies to integrate such differences will be an important challenge for future research.

## Author contributions

This study was primarily conducted by R. K., who performed experiments and machine-learning studies, and by M. H., who processed calculations of the descriptors. T. R., T. N., and K. S. supported the photoreaction experiments, while O. G. M. supervised T. R. The computational method used for calculating rate constants in this study was previously reported by N. I. and T. Y., who also contributed to the descriptor design in this study. The photophysical experiments were carried out under the guidance and discussions led by Y. M., with support from T. O. and supervision by H. I. The overall research was directed by N. N., who was also involved in experiments and machine-learning studies, under the supervision of S. S. The initial manuscript was drafted by N. N. with contributions from R. K., M. H., N. I., T. Y., and Y. M., and was edited by all co-authors.

## Conflicts of interest

There are no conflicts to declare.

## Abbreviations

DFT: Density functional theory
HGB: Histogram-based gradient boosting
ISC: Intersystem crossing
ML: Machine learning
OPS: Organic photosensitizer
RISC: Reverse intersystem crossing
SHAP: Shapley additive explanations
TADF: Thermally activated delayed fluorescence
TD-DFT: Time-dependent density functional theory
TVCF: Thermal vibration correlation function

## Supplementary Material

SC-017-D5SC06465A-s001

## Data Availability

Example code used within this study and corresponding data are available at our GitHub repository (https://github.com/Naoki-Noto/P6-20240509-RK). The data supporting this article have been included in the supplementary information (SI). Supplementary information: full experimental methods including detailed synthetic procedures and characterization data, computational details, and NMR spectra are compiled. See DOI: https://doi.org/10.1039/d5sc06465a.
