# Supplementary material for: Data-driven approach to elucidate the correlation between photocatalytic activity and rate constants from excited states
Source: Chem Sci. 2025 Nov 25;17(1):176–86. doi: 10.1039/d5sc06465a (PMC12700171; doi:10.1039/d5sc06465a)

Supplementary Information (SI) for Chemical Science.
This journal is © The Royal Society of Chemistry 2025

Supplementary Information

Data-driven approach to elucidate the correlation between photocatalytic activity and rate constants from excited states

Ryuga Kunisada,[a] Manami Hayashi,[a] Tabea Rohlfs,[b] Taiki Nagano,[a] Koki Sano,[a] Naoto Inai,[a] Naoki Noto,*[c] Takuya Ogaki,[d] Yasunori Matsui,[d] Hiroshi Ikeda,[d] Olga García Mancheño,[b] Takeshi Yanai,*[a,e] Susumu Saito*[a,c]

[a]Graduate School of Science, Nagoya University, Nagoya 464-8602, Japan.

[b]Organic Chemistry Institute, University of Münster, Münster 48149, Germany.

[c]Integrated Research Consortium on Chemical Sciences (IRCCS), Nagoya University, Nagoya 464-8602, Japan.

[d]Graduate School of Engineering, Osaka Metropolitan University, Sakai, Osaka 599-8531, Japan.

[e]Institute of Transformative Bio-Molecules (WPI-ITbM), Nagoya University, Nagoya 464-8602, Japan.

Table of Contents

Materials and Methods S2
Reaction Apparatuses S2
List of Organic Photosensitizers (OPSs) S3
Investigation into the Catalytic Activity of OPSs S4
Synthesis of Authentic Samples S9
Spectroscopic Measurements for OPSs S12
Experimentally Determined Rate Constants S16
Measurement of Excited-State Lifetimes Used for ML by Transient Absorption Spectroscopy S18
Computational Details for the Design of Descriptors S32
Results of Machine Learning S35
Description of SHAP Plots S39
References S42
NMR Spectra S44

Materials and Methods

All the chemicals for synthesis of the substrates and the catalysts were commercially available and purchased from Tokyo Chemical Industry Co., Ltd., Fujifilm Wako Pure Chemical Corporation, Kanto Chemical Co., Inc., Combi-Blocks, Inc., BLD Pharmatech Ltd., or Sigma-Aldrich Co. LLC. Thin-layer chromatography was performed on TLC plates with silica gel 60 GF254 0.25 nm (Merck). Purification was performed by flash column chromatography on silica gel (Kanto Chemical Co., Inc., spherical, neutral, diameter: 40–100 mm).

Experiments and measurements were carried out with the following apparatuses.

Visible light irradiation: HepatoChem PhotoRedOx Box (λ = 450 nm or 425 nm). NMR: JEOL ECA-600 (600 MHz for ^{1}H NMR) (Reference of ^{1}H NMR spectra: Tetramethylsilane ($CDCl_3$, acetone-d_6: 0.00 ppm), or residual protio impurities in the deuterated solvents (DMSO-d_6 and C_6D_6). Reference of ^{19}F NMR spectra: Trifluoroacetic acid (–76.55 ppm)). HRMS (ESI-QTOF): Bruker compact. GC-MS: Agilent 6850 series network GC system and Agilent 5975C series Mass Selective Detector (EI) [column: HP-5MS capillary column (l = 30 m, d = 0.25 mm, film thickness = 0.25 mm)].

Reaction Apparatus

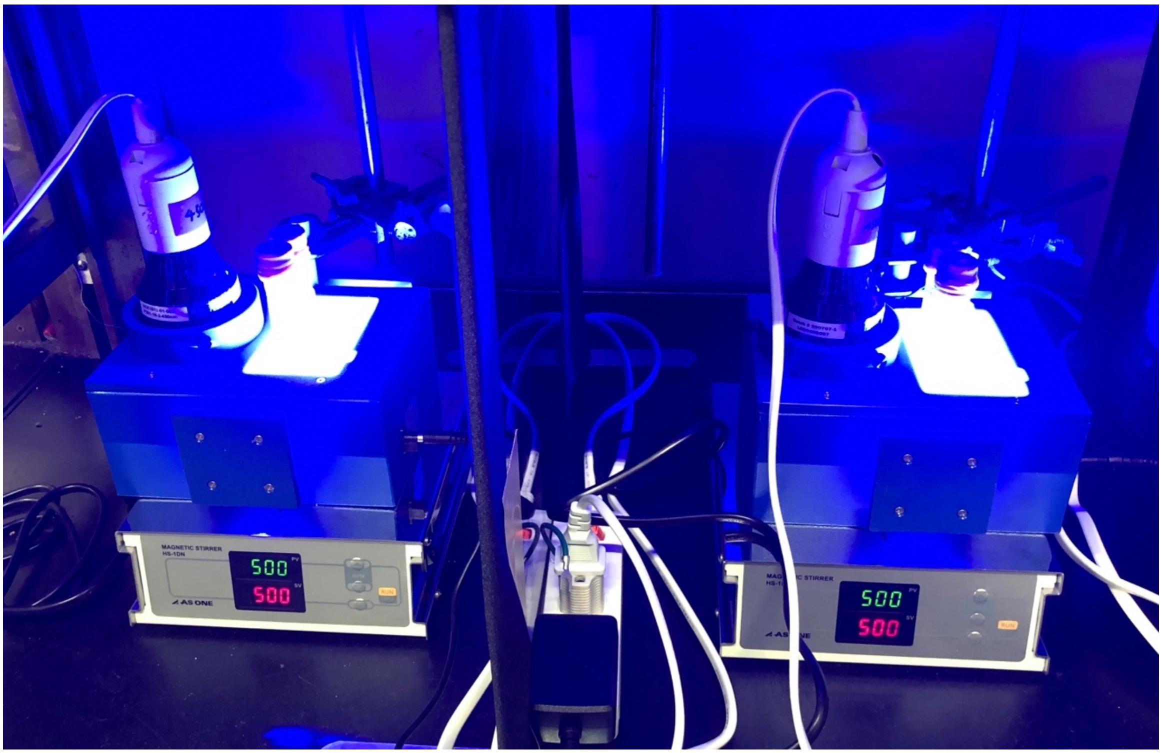

Figure S1. Reaction apparatuses.

List of Organic Photosensitizers (OPSs)[S1]

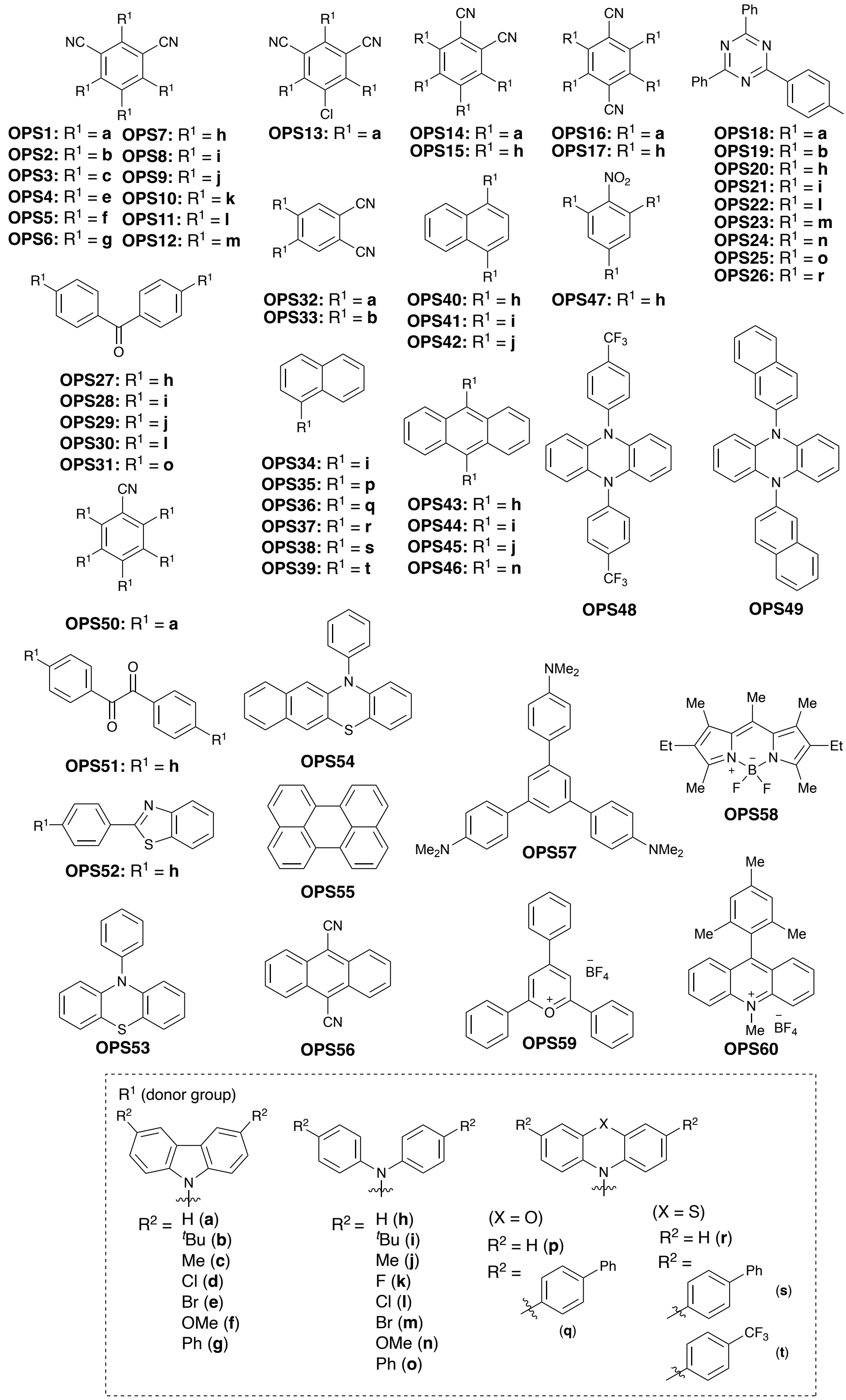

Figure S2. List of organic photosensitizers.

Investigation into the Catalytic Activity of OPSs

The dataset for C–O bond forming reactions (energy transfer-type reactions; **CO-a**, **CO-b**, **CO-c**, **CO-d**, **CO-e**) was prepared in our previous report.[S1] In this research, we have newly constructed the following dataset involved in radical addition reactions (photoredox reactions). The reaction dataset is available in our Github repository at https://github.com/Naoki-Noto/P6-20240509-RK/tree/main/data.

Trifluoromethylation of 1,1-di(*p*-tolyl)ethylene (denoted as **CF_3**)

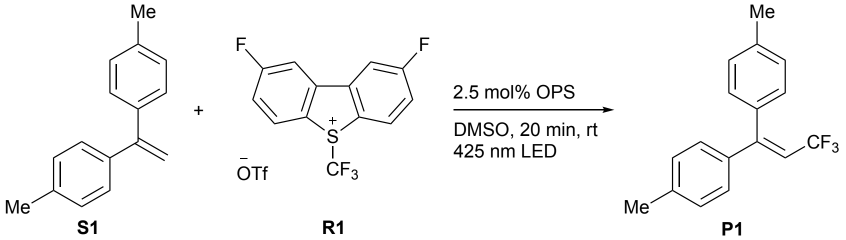

1,1-Di(*p*-tolyl)ethylene (**S1**, 20.8 mg, 0.100 mmol), Umemoto reagent II (**R1**, 52.6 mg, 0.120 mmol), a photosensitizer (0.0025 mmol), and DMSO (1.5 mL) were added to a test tube under air at room temperature. After the mixture was degassed by three freeze-pump-thaw cycles, the test tube was put in PhotoRedOx Box and the reaction was carried out under visible light irradiation (λ = 425 nm) for 20 min at room temperature. Water (15 mL) was added, and the mixture was extracted with Et_2O (15 mL x 3). The organic phase was dried over Na_2SO_4, filtered, and evaporated. The yield was determined by ^{1}H NMR spectroscopy with dibromomethane as an internal standard.

(Commercially available reagents used)
Umemoto reagent II: Combi-Blocks (Cat. No.: QW-8873, Lot. No.: B50765)
DMSO: Wako (Cat. No.: 048-32811, Lot. No.: AC2333)

Table S1. Investigation into the catalytic activity of OPSs in **CF_3**

Name	Yield	Name	Yield	Name	Yield	Name	Yield	Name	Yield
**OPS1**	57	**OPS13**	36	**OPS25**	36	**OPS37**	97	**OPS49**	68
**OPS2**	68	**OPS14**	20	**OPS26**	33	**OPS38**	85	**OPS50**	59
**OPS3**	39	**OPS15**	91	**OPS27**	87	**OPS39**	90	**OPS51**	8
**OPS4**	52	**OPS16**	2	**OPS28**	29	**OPS40**	87	**OPS52**	29
**OPS5**	4	**OPS17**	90	**OPS29**	30	**OPS41**	80	**OPS53**	51
**OPS6**	34	**OPS18**	13	**OPS30**	91	**OPS42**	70	**OPS54**	77
**OPS7**	93	**OPS19**	37	**OPS31**	45	**OPS43**	20	**OPS55**	32
**OPS8**	85	**OPS20**	45	**OPS32**	46	**OPS44**	3	**OPS56**	4
**OPS9**	85	**OPS21**	33	**OPS33**	67	**OPS45**	11	**OPS57**	27
**OPS10**	92	**OPS22**	43	**OPS34**	37	**OPS46**	40	**OPS58**	9
**OPS11**	88	**OPS23**	50	**OPS35**	48	**OPS47**	10	**OPS59**	6
**OPS12**	90	**OPS24**	36	**OPS36**	89	**OPS48**	61	**OPS60**	9

2,2,2-trifluoroethylation of 1,1-di(*p*-tolyl)ethylene (denoted as **CH_2CF_3**)

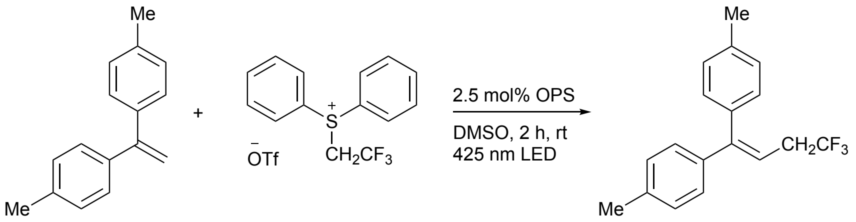

1,1-Di(*p*-tolyl)ethylene (**S1**, 20.8 mg, 0.100 mmol), diphenyl(2,2,2-trifluoroethyl)sulfonium triflate (**R2**, 62.8 mg, 0.150 mmol), a photosensitizer (0.0025 mmol), and DMSO (1.5 mL) were added to a test tube under air at room temperature. After the mixture was degassed by three freeze-pump-thaw cycles, the test tube was put in PhotoRedOx Box and the reaction was carried out under visible light irradiation (λ = 425 nm) for 2 h at room temperature. Water (15 mL) was added, and the mixture was extracted with Et_2O (15 mL x 3). The organic phase was dried over Na_2SO_4, filtered, and evaporated. The yield was determined by ^{1}H NMR spectroscopy with dibromomethane as an internal standard.

(Commercially available reagents used)
DMSO: Wako (Cat. No.: 048-32811, Lot. No.: AC2333)

Table S2. Investigation into the catalytic activity of OPSs in **CH_2CF_3**

Name	Yield	Name	Yield	Name	Yield	Name	Yield	Name	Yield
**OPS1**	20	**OPS13**	20	**OPS25**	35	**OPS37**	39	**OPS49**	74
**OPS2**	0	**OPS14**	0	**OPS26**	8	**OPS38**	63	**OPS50**	6
**OPS3**	0	**OPS15**	13	**OPS27**	63	**OPS39**	77	**OPS51**	0
**OPS4**	51	**OPS16**	0	**OPS28**	37	**OPS40**	62	**OPS52**	34
**OPS5**	0	**OPS17**	7	**OPS29**	30	**OPS41**	50	**OPS53**	28
**OPS6**	0	**OPS18**	14	**OPS30**	71	**OPS42**	54	**OPS54**	71
**OPS7**	55	**OPS19**	39	**OPS31**	53	**OPS43**	58	**OPS55**	60
**OPS8**	48	**OPS20**	41	**OPS32**	0	**OPS44**	26	**OPS56**	0
**OPS9**	68	**OPS21**	53	**OPS33**	0	**OPS45**	32	**OPS57**	24
**OPS10**	48	**OPS22**	41	**OPS34**	38	**OPS46**	29	**OPS58**	2
**OPS11**	37	**OPS23**	32	**OPS35**	54	**OPS47**	0	**OPS59**	1
**OPS12**	35	**OPS24**	42	**OPS36**	83	**OPS48**	51	**OPS60**	2

Monofluoromethylation of 1,1-di(*p*-tolyl)ethylene (denoted as **CH_2F**)

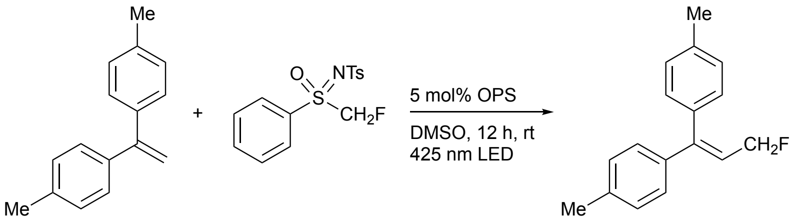

1,1-Di(*p*-tolyl)ethylene (**S1**, 20.8 mg, 0.100 mmol), *N*-((fluoromethyl)(oxo)(phenyl)-λ^6-sulfaneylidene)-4-methylbenzenesulfonamide (**R3**, 49.1 mg, 0.150 mmol), a photosensitizer (0.0050 mmol), and DMSO (1.5 mL) were added to a test tube under air at room temperature. After the mixture was degassed by three freeze-pump-thaw cycles, the test tube was put in PhotoRedOx Box and the reaction was carried out under visible light irradiation (λ = 425 nm) for 12 h at room temperature. After the completion of the reaction, the DMSO solution was extracted with Et_2O (15 mL x 3). Na_2SO_4 was added to the organic phase, and it was filtered and evaporated. The yield was determined by ^{1}H NMR spectroscopy with 1,3,5-trimethoxybenzene as an internal standard.

(Commercially available reagents used)

DMSO: Wako (Cat. No.: 048-32811, Lot. No.: AC2333)

Table S3. Investigation into the catalytic activity of OPSs in **CH_2F**

Name	Yield	Name	Yield	Name	Yield	Name	Yield	Name	Yield
**OPS1**	1	**OPS13**	0	**OPS25**	0	**OPS37**	57	**OPS49**	26
**OPS2**	0	**OPS14**	0	**OPS26**	0	**OPS38**	52	**OPS50**	1
**OPS3**	0	**OPS15**	0	**OPS27**	4	**OPS39**	57	**OPS51**	0
**OPS4**	0	**OPS16**	0	**OPS28**	1	**OPS40**	56	**OPS52**	1
**OPS5**	0	**OPS17**	0	**OPS29**	4	**OPS41**	59	**OPS53**	30
**OPS6**	0	**OPS18**	0	**OPS30**	0	**OPS42**	48	**OPS54**	28
**OPS7**	28	**OPS19**	0	**OPS31**	10	**OPS43**	0	**OPS55**	14
**OPS8**	11	**OPS20**	2	**OPS32**	0	**OPS44**	4	**OPS56**	0
**OPS9**	14	**OPS21**	0	**OPS33**	0	**OPS45**	9	**OPS57**	11
**OPS10**	2	**OPS22**	0	**OPS34**	27	**OPS46**	2	**OPS58**	0
**OPS11**	6	**OPS23**	0	**OPS35**	38	**OPS47**	0	**OPS59**	0
**OPS12**	2	**OPS24**	0	**OPS36**	42	**OPS48**	25	**OPS60**	0

Alkylation of 1,1-di(*p*-tolyl)ethylene (denoted as **Cy**)

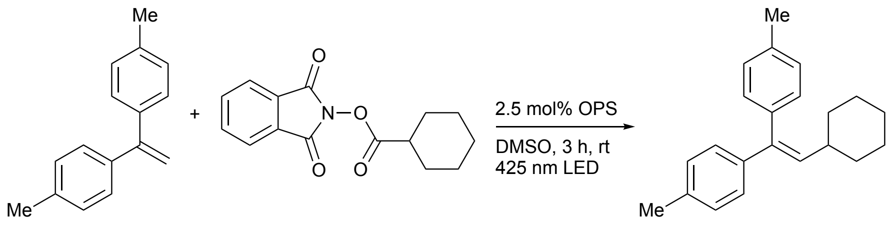

1,1-Di(*p*-tolyl)ethylene (**S1**, 20.8 mg, 0.100 mmol), 1,3-dihydro-1,3-dioxo-2H-isoindol-2-yl cyclohexanecarboxylate (**R4**, 41.0 mg, 0.150 mmol), a photosensitizer (0.0025 mmol), and DMSO (1.5 mL) were added to a test tube under air at room temperature. After the mixture was degassed by three freeze-pump-thaw cycles, the test tube was put in PhotoRedOx Box and the reaction was carried out under visible light irradiation (λ = 425 nm) for 3 h at room temperature. Water (15 mL) was added, and the mixture was extracted with Et_2O (15 mL x 3). The organic phase was dried over Na_2SO_4, filtered, and evaporated. The yield was determined by ^1H NMR spectroscopy with 1,3,5-trimethoxybenzene as an internal standard.

(Commercially available reagents used)

1,3-Dihydro-1,3-dioxo-2*H*-isoindol-2-yl cyclohexanecarboxylate: BLDpharm (Cat. No.: BD01145079, Lot. No.: CRU828)

DMSO: Wako (Cat. No.: 048-32811, Lot. No.: AC2333)

Table S4. Investigation into the catalytic activity of OPSs in **Cy**

Name	Yield	Name	Yield	Name	Yield	Name	Yield	Name	Yield
**OPS1**	45	**OPS13**	40	**OPS25**	88	**OPS37**	85	**OPS49**	73
**OPS2**	12	**OPS14**	9	**OPS26**	55	**OPS38**	91	**OPS50**	44
**OPS3**	8	**OPS15**	91	**OPS27**	82	**OPS39**	88	**OPS51**	7
**OPS4**	22	**OPS16**	6	**OPS28**	84	**OPS40**	88	**OPS52**	85
**OPS5**	0	**OPS17**	91	**OPS29**	86	**OPS41**	87	**OPS53**	44
**OPS6**	5	**OPS18**	36	**OPS30**	79	**OPS42**	91	**OPS54**	68
**OPS7**	90	**OPS19**	34	**OPS31**	77	**OPS43**	20	**OPS55**	58
**OPS8**	91	**OPS20**	88	**OPS32**	22	**OPS44**	5	**OPS56**	0
**OPS9**	98	**OPS21**	89	**OPS33**	60	**OPS45**	32	**OPS57**	20
**OPS10**	90	**OPS22**	82	**OPS34**	73	**OPS46**	26	**OPS58**	4
**OPS11**	81	**OPS23**	72	**OPS35**	91	**OPS47**	28	**OPS59**	0
**OPS12**	80	**OPS24**	66	**OPS36**	94	**OPS48**	65	**OPS60**	0

Thiotrifluoromethylation of 1,1-di(*p*-tolyl)ethylene (denoted as **SCF_3**)

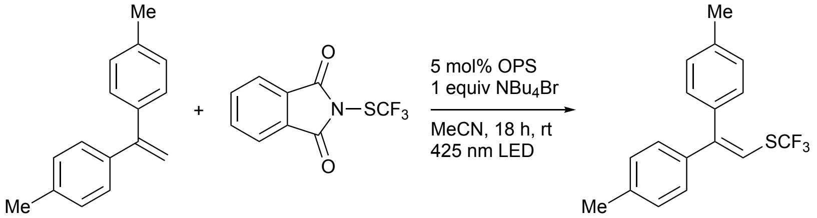

1,1-Di(*p*-tolyl)ethylene (**S1**, 20.8 mg, 0.100 mmol), *N*-(trifluoromethylthio)phthalimide (**R5**, 32.1 mg, 0.130 mmol), a photosensitizer (0.0050 mmol), tetrabutylammonium bromide (32.2 mg, 0.100 mmol) and MeCN (1.5 mL) were added to a test tube under air at room temperature. After the mixture was degassed by three freeze-pump-thaw cycles, the test tube was put in PhotoRedOx Box and the reaction was carried out under visible light irradiation (λ = 425 nm) for 18 h at room temperature. Water (15 mL) was added, and the mixture was extracted with Et_2O (15 mL x 3). The organic phase was dried over Na_2SO_4, filtered, and evaporated. The yield was determined by ^{1}H NMR spectroscopy with 1,3,5-trimethoxybenzene as an internal standard.

(Commercially available reagents used)
N-(Trifluoromethylthio)phthalimide: TCI (Cat. No.: T3143, Lot. No.: MG6QC-OQ)
NBu_4Br: TCI (Cat. No.: T0054, Lot. No.:NL63L-RR)
MeCN: Wako (Cat. No.: 010-22905, Lot. No.: ACJ1191)

Table S5. Investigation into the catalytic activity of OPSs in **SCF_3**

Name	Yield	Name	Yield	Name	Yield	Name	Yield	Name	Yield
**OPS1**	86	**OPS13**	77	**OPS25**	89	**OPS37**	50	**OPS49**	1
**OPS2**	70	**OPS14**	76	**OPS26**	85	**OPS38**	94	**OPS50**	87
**OPS3**	27	**OPS15**	90	**OPS27**	88	**OPS39**	95	**OPS51**	21
**OPS4**	80	**OPS16**	37	**OPS28**	91	**OPS40**	68	**OPS52**	88
**OPS5**	12	**OPS17**	32	**OPS29**	91	**OPS41**	43	**OPS53**	31
**OPS6**	74	**OPS18**	83	**OPS30**	87	**OPS42**	52	**OPS54**	94
**OPS7**	84	**OPS19**	87	**OPS31**	75	**OPS43**	44	**OPS55**	76
**OPS8**	90	**OPS20**	86	**OPS32**	82	**OPS44**	54	**OPS56**	31
**OPS9**	94	**OPS21**	92	**OPS33**	92	**OPS45**	21	**OPS57**	26
**OPS10**	91	**OPS22**	88	**OPS34**	94	**OPS46**	14	**OPS58**	2
**OPS11**	79	**OPS23**	90	**OPS35**	68	**OPS47**	4	**OPS59**	37
**OPS12**	82	**OPS24**	62	**OPS36**	90	**OPS48**	5	**OPS60**	0

Synthesis of Authentic Samples

Synthesis of 4,4'-(3,3,3-Trifluoroprop-1-ene-1,1-diyl)bis(methylbenzene) (**P1**)

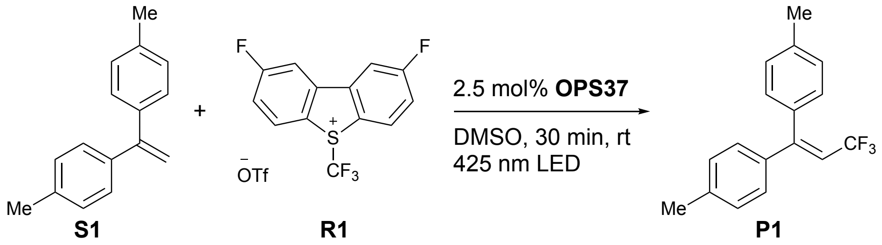

1,1-Di(*p*-tolyl)ethylene (**S1**, 41.7 mg, 0.200 mmol), Umemoto reagent II (**R1**, 105 mg, 0.240 mmol), **OPS37** (1.6 mg, 4.92 mmol), and DMSO (3 mL) were added to a test tube under air at room temperature. After the mixture was degassed by three freeze-pump-thaw cycles, the test tube was put in PhotoRedOx Box and the reaction was carried out under visible light irradiation (λ = 425 nm) for 30 min at room temperature. Water (15 mL) was added, and the mixture was extracted with Et_2O (15 mL x 3). The organic phase was dried over Na_2SO_4, filtered, and evaporated. The desired product (**P1**) was obtained as a colorless oil (42.6 mg, 0.154 mmol, 77%) after the purification through column chromatography on silica-gel (hexane/ethyl acetate = 20:1) and recycling preparative HPLC.

Spectral data agree with the reported literature.[S2]

^{1}H NMR (600 MHz, $CDCl_3$, rt): δ 7.19–7.17 (m, 2H), 7.14–7.11 (6H), 6.05 (q, *J* = 8.4 Hz, 1H), 2.39 (s, 3H), 2.35 (s, 3H). **^{13}C NMR** (151 MHz, $CDCl_3$, rt): δ 152.6 (q, *J* = 5.9 Hz), 139.6, 138.5, 137.7, 134.7, 129.3, 129.2, 128.8, 128.1, 123.4 (q, *J* = 270.6 Hz), 114.4 (q, *J* = 33.2 Hz), 21.5, 21.3. **^{19}F NMR** (564 MHz, $CDCl_3$, rt): δ –56.5 (d, *J* = 6.8 Hz).

Synthesis of 4,4'-(4,4,4-Trifluorobut-1-ene-1,1-diyl)bis(methylbenzene) (**P2**)

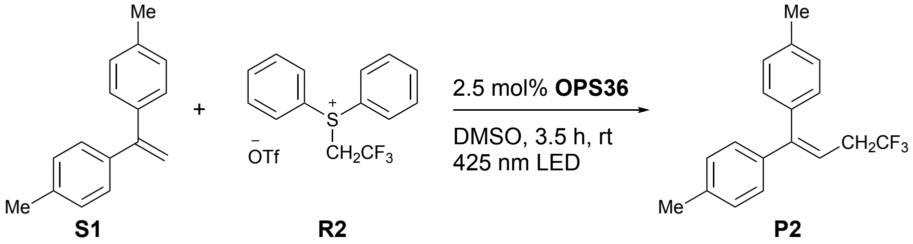

1,1-Di(*p*-tolyl)ethylene (**S1**, 41.7 mg, 0.200 mmol), diphenyl(2,2,2-trifluoroethyl)sulfonium triflate (**R2**, 100 mg, 0.240 mmol), **OPS36** (3.1 mg, 5.05 mmol), and DMSO (3 mL) were added to a test tube under air at room temperature. After the mixture was degassed by three freeze-pump-thaw cycles, the test tube was put in PhotoRedOx Box and the reaction was carried out under visible light irradiation (λ = 425 nm) for 3.5 h at room temperature. Water (15 mL) was added, and the mixture was extracted with Et_2O (15 mL x 3). The organic phase was dried over Na_2SO_4, filtered, and evaporated. The desired product (**P2**) was obtained as a colorless oil (39.5 mg, 0.136 mmol, 68%) after the purification through column chromatography on silica-gel (hexane) and recycling preparative HPLC.

^{1}H NMR (600 MHz, $CDCl_3$, rt): δ 7.20 (d, *J* = 7.8 Hz, 2H), 7.13 (d, *J* = 8.4 Hz, 2H), 7.08 (d, *J* = 8.4 Hz, 2H),

7.05 (d, J = 7.8 Hz, 2H), 6.00 (t, J = 7.2 Hz, 1H), 2.93–2.87 (m, 2H), 2.39 (s, 3H), 2.33 (s, 3H). **^{13}C NMR** (151 MHz, $CDCl_3$, rt): δ 147.6, 139.0, 137.8, 137.5, 136.0, 129.6, 129.3, 129.1, 127.4, 126.4 (q, J = 277.8 Hz), 115.1, 34.9 (q, J = 29.0 Hz), 21.4, 21.2. **^{19}F NMR** (564 MHz, $CDCl_3$, rt): δ –66.8 (t, J = 11.3 Hz).
HRMS (ESI-QTOF) calcd m/z for $[C_{18}H_{17}F+Na]^+$ 313.1175 found 313.1177.

Synthesis of 4,4'-(3-Fluoroprop-1-ene-1,1-diyl)bis(methylbenzene) (**P3**)

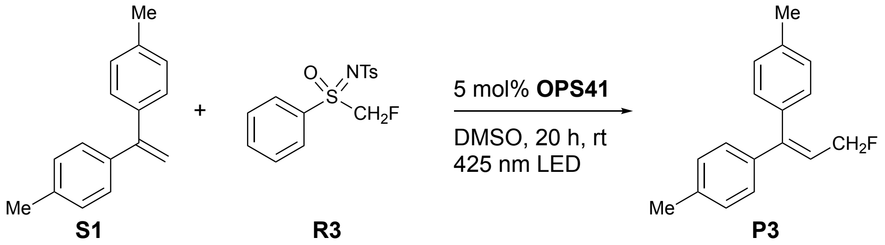

1,1-Di(*p*-tolyl)ethylene (**S1**, 41.7 mg, 0.200 mmol), *N*-((fluoromethyl)(oxo)(phenyl)-λ^6-sulfaneylidene)-4-methylbenzenesulfonamide (**R3**, 98.2 mg, 0.300 mmol), **OPS41** (6.9 mg, 10.0 mmol), and DMSO (3 mL) were added to a test tube under air at room temperature. After the mixture was degassed by three freeze-pump-thaw cycles, the test tube was put in PhotoRedOx Box and the reaction was carried out under visible light irradiation (λ = 425 nm) for 20 h at room temperature. After the completion of the reaction, the DMSO solution was extracted with hexane (15 mL x 3). Na_2SO_4 was added to the organic phase, and it was filtered and evaporated. The desired product (**P3**) was obtained as a colorless oil (28.1 mg, 0.117 mmol, 58%) after the purification through recycling preparative HPLC.
^{1}H NMR (600 MHz, $CDCl_3$, rt): δ 7.19–7.05 (8H), 6.23 (dt, J = 10.8 Hz, 7.8 Hz, 1H), 4.92 (dd, J = 47.4 Hz, 7.2 Hz, 2H), 2.39 (s, 3H), 2.35 (s, 3H). **^{13}C NMR** (151 MHz, $CDCl_3$, rt): δ 147.7 (d, J = 11.6 Hz), 138.9 (d, J = 3.0 Hz), 138.1, 137.8, 135.7, 129.8, 129.1, 129.0, 127.9, 121.5 (d, J = 17.4 Hz), 80.9 (d, J = 157.6 Hz), 21.4, 21.3. **^{19}F NMR** (564 MHz, $CDCl_3$, rt): δ –206.3 (dt, J = 47.4 Hz, 10.7 Hz).
HRMS (ESI-QTOF) calcd m/z for $[C_{17}H_{17}F+Na]^+$ 263.1206 found 263.1206.

Synthesis of 4,4'-(2-Cyclohexylethene-1,1-diyl)bis(methylbenzene) (**P4**)

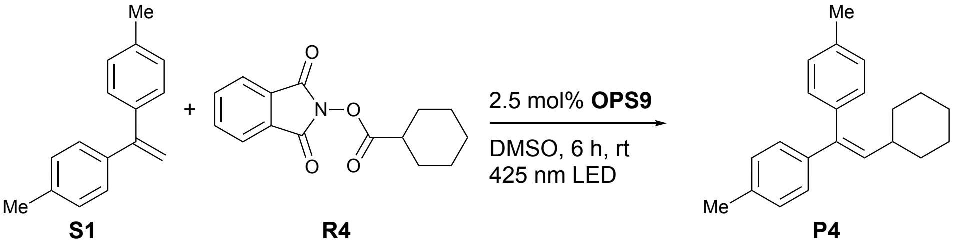

1,1-Di(*p*-tolyl)ethylene (**S1**, 41.7 mg, 0.200 mmol), 1,3-dihydro-1,3-dioxo-2H-isoindol-2-yl cyclohexanecarboxylate (**R4**, 82.0 mg, 0.300 mmol), **OPS9** (4.5 mg, 4.95 mmol), and DMSO (3 mL) were added to a test tube under air at room temperature. After the mixture was degassed by three freeze-pump-thaw cycles, the test tube was put in PhotoRedOx Box and the reaction was carried out under visible light irradiation (λ = 425 nm) for 6 h at room temperature. Water (15 mL) was added, and the mixture was

extracted with Et_2O (15 mL x 3). The organic phase was dried over Na_2SO_4, filtered, and evaporated. The desired product (**P5**) was obtained as a white solid (44.3 mg, 0.153 mmol, 76%) after the purification through column chromatography on silica-gel (hexane) and recycling preparative HPLC.

Spectral data agree with the reported literature.[S3]

^{1}H NMR (600 MHz, $CDCl_3$, rt): δ 7.17-7.04 (m, 8H), 5.82 (d, J = 10.0 Hz, 1H), 2.38 (s, 3H), 2.31 (s, 3H), 2.14-2.13 (m, 1H), 1.67-1.59 (m, 5H), 1.25-1.15 (m, 5H). **^{13}C NMR** (151 MHz, $CDCl_3$, rt): δ 140.6, 139.4, 137.9, 136.5, 136.4, 135.2, 129.8, 128.9, 128.8, 127.3, 38.4, 33.6, 26.2, 25.8, 21.4, 21.2

Synthesis of (2,2-Di-*p*-tolylvinyl)(trifluoromethyl)sulfane (**P5**)

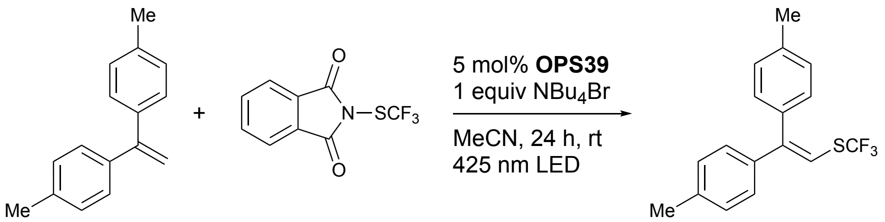

1,1-Di(*p*-tolyl)ethylene (**S1**, 41.7 mg, 0.200 mmol), *N*-(trifluoromethylthio)phthalimide (**R5**, 64.3 mg, 0.260 mmol), **OPS39** (6.3 mg, 10.0 mmol), tetrabutylammonium bromide (64.5 mg, 0.200 mmol) and MeCN (3 mL) were added to a test tube under air at room temperature. After the mixture was degassed by three freeze-pump-thaw cycles, the test tube was put in PhotoRedOx Box and the reaction was carried out under visible light irradiation (λ = 425 nm) for 24 h at room temperature. Water (15 mL) was added, and the mixture was extracted with Et_2O (15 mL x 3). The organic phase was dried over Na_2SO_4, filtered, and evaporated. The desired product (**P5**) was obtained as a colorless oil (55.0 mg, 0.178 mmol, 89%) after the purification through column chromatography on silica-gel (hexane/ethyl acetate = 20:1) and recycling preparative HPLC.

Spectral data agree with the reported literature.[S4]

^{1}H NMR (600 MHz, $CDCl_3$, rt): δ 7.22 (d, *J* = 8.4 Hz, 2H), 7.14–7.09 (6H), 6.60 (s, 1H), 2.40 (s, 3H), 2.35 (s, 3H). **^{13}C NMR** (151 MHz, $CDCl_3$, rt): δ 146.8, 138.6, 138.5, 137.9, 135.4, 130.0 (q, *J* = 308.2 Hz), 129.43, 129.40, 127.5, 110.8 (q, *J* = 2.9 Hz), 21.5, 21.3. **^{19}F NMR** (564 MHz, $CDCl_3$, rt): δ –43.9.

Spectroscopic Measurements for OPSs

(1) General information

Photoluminescence spectra were recorded on FP-8500 spectrophotometers. Absolute fluorescence (FL) quantum yields were determined by utilizing the integrating sphere method with a Hamamatsu Photonics C9920-02 absolute photoluminescence quantum yields measurement system. FL decay profiles were measured by using a HORIBA Jobin Yvon FluoroCube lifetime spectrofluorometer equipped with HORIBA NanoLED-370 and analyzed using DAS6 FL decay analysis software. Transient absorption spectra were recorded using a picoTAS system (Unisoku Co., Ltd.) based on a Randomly-Interleaved-Pulse-Train method. The pump laser used was a wavelength-tunable picosecond laser (PL-2210A and PG403, EKSPLA) with a pulse width of 25 ps.

(2) Measurement of fluorescence quantum yield and lifetime

Measurements of fluorescence quantum yield and fluorescence decay profiles were performed under Ar in toluene. The sample solutions were degassed with Ar for 10 minutes of bubbling before measurements. To obtain the experimentally determined rate constants, measurements were also conducted under air for TADF (**OPS5**, **OPS7**).

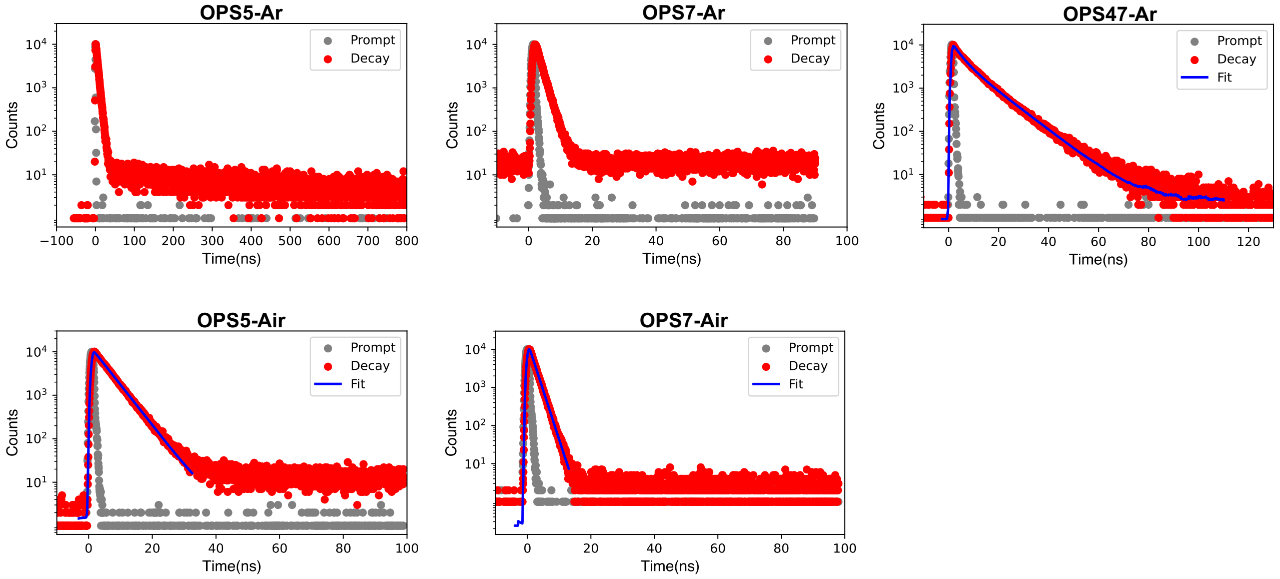

Figure S3. Result of FL lifetime measurement under Ar and Air

Table S6. Results of FL quantum yields and lifetimes

OPS	$\Phi_{FL,(air)}$	$\Phi_{FL,(Ar)}$	$\tau_{prompt,(air)}$ (ns)	$\tau_{prompt,(Ar)}$ (ns)	$\tau_{delay,(Ar)}$ (ns)
**OPS5**	0.05	0.06	4.66	4.78	616
**OPS7**	0.08	0.20	1.68	1.68	34400
**OPS47**	–	0.02	–	3.51	9.86

Φ: Photoluminescence quantum yield, τ_{prompt} : Prompt emission, τ_{delay} : Delayed emission

Note: The fluorescence decay profile of **OPS47** was also fitted with a double exponential function. However, since the lifetime of the delayed component was less than 10 ns, **OPS47** was determined not to be a TADF compound. Therefore, the shorter component (3.51 ns) was assigned to the prompt excited-state lifetime.

(3) Measurement of ΔE_{ST}

The sample solutions (solvent: 2-methyltetrahydrofuran) were degassed by three freeze (77 K)–pump (0.1 mmHg)–thaw (RT) cycles before measurements. Fluorescence and phosphorescence spectra were measured at room temperature and at 77 K using liquid nitrogen, respectively. The ΔE_{ST} values were determined from the difference in peak energies of the fluorescence and phosphorescence spectra.

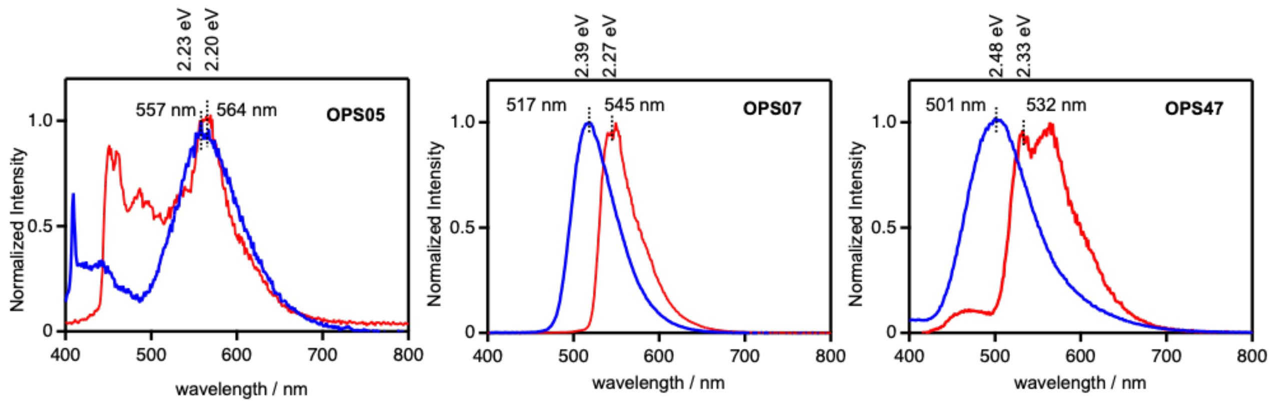

Figure S4. Fluorescence and phosphorescence spectra of **OPS5**, **OPS7**, and **OPS47**

Table S7. Experimentally determined ΔE_{ST} values

**OPS1** (eV)	**OPS5** (eV)	**OPS7** (eV)	**OPS47** (eV)
0.08[a]	0.03	0.12	0.15

[a] Literature data measured in toluene, which is taken from ref. [S5].

Note 1: In contrast, the computed values (**OPS1**: 0.37 eV, **OPS5**: 0.39 eV, **OPS7**: 0.70 eV, **OPS47**: 0.42 eV) were obtained by calculations at the PCM(toluene)-CAM-B3LYP-D3/6-31G(d) level.

Note 2: For **OPS1**, **OPS5**, and **OPS7**, these values were not employed in the determination of the experimental rate constants. However, they were used in the quantum chemical calculations where the experimentally determined ΔE_{ST} values were applied.

(4) Multi-wavelength global analysis[S6]

To determine the rate constants $k_{isc}(S_1 \rightarrow T_1)$ and $k_{risc}(T_1 \rightarrow S_0)$ for **OPS47**, transient absorption measurements were conducted using picoTAS (excited at 355 nm). Multi-wavelength global analysis was carried out using Glotaran 1.5 software to obtain rate constants involved in the intersystem crossing and to resolve the dynamics of the S_1 and T_1 species.

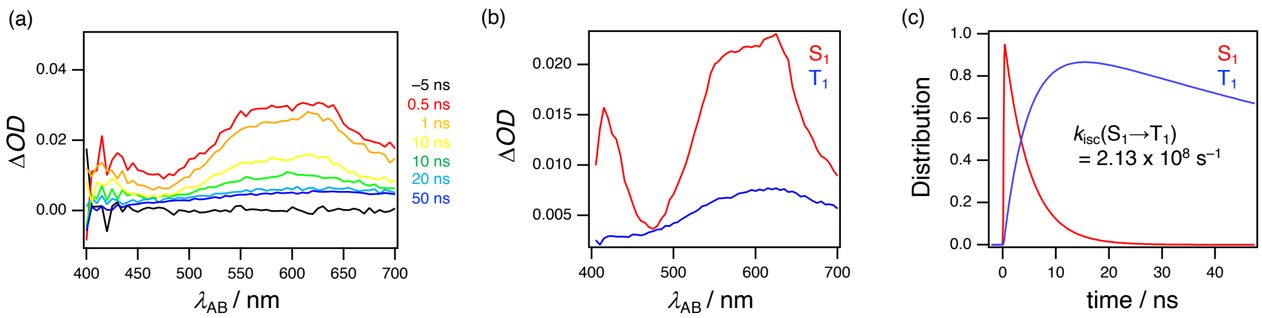

Figure S5. Result of global analysis for **OPS47**. (a) Transient absorption spectrum observed upon excitation of **OPS47** in Ar-saturated toluene. (b) Absorption spectra of the S_1 (red) and T_1 (blue) species obtained by multi-wavelength global analysis. (c) Time-dependent changes of distribution of the S_1 (red) and T_1 (blue) species.

Table S8. Result of global analysis

OPS	$\tau_{S_1 \rightarrow T_1 (Ar)}$ (ns)	$\tau_{T_1 \rightarrow S_0 (Ar)}$ (ns)
**OPS47**	4.68	106

Experimentally Determined Rate Constants

The experimentally determined rate constants were calculated using two distinct methods, depending on whether OPSs exhibited TADF or not.

[Method-A]

For OPSs exhibiting TADF (**OPS5**, **OPS7**), it is possible to calculate the rate constants for several excited-state processes by measuring the fluorescence quantum yield and fluorescence lifetimes under an Ar atmosphere and air. In this case, three rate constants $k_r(S_1 \rightarrow S_0)$, $k_{isc}(S_1 \rightarrow T_1)$, and $k_{risc}(T_1 \rightarrow S_1)$ were obtained, similar to previous studies (for instance, ref. [S7]).

$$k_p = 1 \,/\, \tau_{p,(Ar)}$$

$$k_d = 1 \,/\, \tau_{d,(Ar)}$$

$$\Phi_p = \Phi_{FL,(air)} \times \tau_{p,(Ar)} \,/\, \tau_{p,(air)}$$

$$\Phi_d = \Phi_{FL,(Ar)} - \Phi_p$$

$$k_r(S_1 \rightarrow S_0) = \Phi_p \times k_p + \Phi_d \times k_d \approx \Phi_p \times k_p$$

$$k_{isc}(S_1 \rightarrow S_0) = (1 - \Phi_p) \times k_p$$

$$k_{risc}(T_1 \rightarrow S_1) = \frac{k_p \times k_d \times \Phi_d}{k_{isc}(S_1 \rightarrow T_1) \times \Phi_p}$$

The $\tau_{p,(Ar)}$, $\tau_{d,(Ar)}$, and $\tau_{p,(air)}$ values were experimentally obtained by the analysis of fluorescence decay profiles under an Ar atmosphere or air. The $\Phi_{FL,(Ar)}$ and $\Phi_{FL,(air)}$ values were also experimentally measured under an Ar atmosphere or air. The physical properties used in this method are listed in **Table S9**. The experiments required for their determination are provided in the "Measurement of fluorescence quantum yield and lifetime" section.

Table S9. Physical properties used in method-A

OPS	$\Phi_{FL,(air)}$	$\Phi_{FL,(Ar)}$	$\tau_{prompt,(Air)}$ (ns)	$\tau_{prompt,(Ar)}$ (ns)	$\tau_{delay,(Ar)}$ (ns)
**OPS5**	0.05	0.06	4.66	4.78	616
**OPS7**	0.08	0.20	1.68	1.68	34400

[Method-B]

For an OPS that does not exhibit TADF (**OPS47**), the fluorescence quantum yield and fluorescence lifetime were measured under an Ar atmosphere to calculate the $k_r(S_1 \rightarrow S_0)$ value. The $k_{isc}(S_1 \rightarrow T_1)$ and $k_{isc}(T_1 \rightarrow S_0)$ values were determined by performing transient absorption measurements, followed by multi-wavelength global analysis to deconvolute the singlet and triplet species. The $k_{ic}(S_1 \rightarrow S_0)$ value was calculated by subtracting the rate constant of the radiative decay process (from S_1 to S_0) and that of the intersystem crossing process (from S_1 to T_1) from the inverse of the fluorescence lifetime. Meanwhile, the $k_{risc}(T_1 \rightarrow S_1)$ value was determined through the measurement of the ΔE_{ST} value from the difference between fluorescence and phosphorescence spectra and applying it to the following Boltzmann distribution equation to back-calculate from $k_{isc}(S_1 \rightarrow T_1)$.

$$k_p = 1 / \tau_{p,(Ar)}$$

$$k_{ems}(S_1 \rightarrow S_0) = \Phi_{FL,(Ar)} \times k_p$$

$$k_{isc}(S_1 \rightarrow T_1) = 1 / \tau_{S_1 \rightarrow T_1 (Ar)}$$

$$k_{ic}(S_1 \rightarrow S_0) = k_p - k_{ems}(S_1 \rightarrow S_0) - k_{isc}(S_1 \rightarrow T_1)$$

$$k_{isc}(T_1 \rightarrow S_0) = 1 / \tau_{T_1 \rightarrow S_0 (Ar)}$$

$$k_{risc}(T_1 \rightarrow S_1) = k_{isc}(S_1 \rightarrow T_1) \times e^{\frac{-\Delta E_{ST}}{RT}}$$

The $\tau_{p,(Ar)}$ value was experimentally obtained by analysis of the fluorescence decay profile. The $\tau_{S_1 \rightarrow T_1 (Ar)}$ and $\tau_{T_1 \rightarrow S_0 (Ar)}$ values were obtained by transient absorption spectroscopic analysis with picoTAS,[S8] followed by the multi-wavelength global analysis. The ΔE_{ST} value was measured value (**Table S10**). The experiments required for their determination are provided in the "Measurement of fluorescence quantum yield and lifetime", "Measurement of ΔE_{ST}", and "Multi-wavelength global analysis" sections.

Table S10. Physical properties used in Method-B

	$\Phi_{FL,(Ar)}$	$\tau_{prompt,(Ar)}$ (ns)	$\tau_{S_1 \rightarrow T_1 (Ar)}$ (ns)	$\tau_{T_1 \rightarrow S_0 (Ar)}$ (ns)	ΔE_{ST} (eV)
**OPS47**	0.02	3.51	4.68	106	0.15

Table S11. Experimentally determined rate constants

**OPS**	$k_r(S_1 \rightarrow S_0)$ (s^{-1})	$k_{ic}(S_1 \rightarrow S_0)$ (s^{-1})	$k_{isc}(S_1 \rightarrow T_1)$ (s^{-1})	$k_{risc}(T_1 \rightarrow S_1)$ (s^{-1})	$k_{isc}(T_1 \rightarrow S_0)$ (s^{-1})
**OPS1**	1.7×10^{7}[a]	1.5×10^{6}[a]	5.1×10^{7}[a]	2.7×10^{6}[a]	–
**OPS5**	1.1×10^{7}	–	2.0×10^{8}	2.9×10^{5}	–
**OPS7**	4.8×10^{7}	–	5.5×10^{8}	4.7×10^{4}	–
**OPS47**	5.7×10^{6}	6.6×10^{7}	2.1×10^{8}	6.2×10^{5}	9.4×10^{6}

Method-A was used for **OPS5** and **OPS7**, while Method-B was used for **OPS47**. [a] Rate constants of **OPS1** are taken from ref. [S9].

Measurement of Excited-State Lifetimes Used for ML by Transient Absorption Spectroscopy

(1) Methods for measurement

The measurement of excited-state lifetimes by transient absorption spectroscopy was performed under an Ar atmosphere in toluene or DMSO using picoTAS.[S8] In cases where solubility issues made measurements difficult, MeCN was used as an alternative solvent to toluene, and DMF was used as an alternative solvent to DMSO. The sample concentration was adjusted to ensure that the absorbance at 355 nm (the excitation wavelength for the transient absorption measurements) was between 0.6 and 0.9, unless there were any issues. The sample solutions were degassed by bubbling Ar for 10 minutes before the measurements.

(2) Methods for fitting

In this study, the OPSs were classified into three types. The first type consists of compounds whose decay curves contain a single component that can be fitted with a single exponential function. In this case, the measured lifetime itself was used as the descriptor. The second type includes compounds with decay curves that can be fitted with a double exponential function but were determined not to exhibit delayed fluorescence. For these compounds, the shorter excited-state lifetime of the two components was used as the descriptor. The third type corresponds to TADF compounds, for which the longer excited-state lifetime was used as the descriptor.

Table S12. Excited-state lifetimes of each photosensitizer in transient absorption measurements

OPS	LT_t (ns)	LT_d (ns)	OPS	LT_t (ns)	LT_d (ns)	OPS	LT_t (ns)	LT_d (ns)
**OPS1**	1.2×10^{3}	1.6×10^{3}	**OPS21**	1.5×10^{4}	3.1×10^{4}	**OPS41**	7.2	1.6×10^{1}
**OPS2**	9.5×10^{2}	9.0×10^{2}	**OPS22**	7.8×10^{2}	3.1×10^{4}	**OPS42**	7.3	1.2×10^{1}
**OPS3**	1.2×10^{3}	1.1×10^{3}	**OPS23**	1.7×10^{3}	2.0×10^{4}	**OPS43**	1.5×10^{1}	3.5×10^{1}
**OPS4**	1.4×10^{3}	4.1×10^{2}	**OPS24**	6.9	1.0×10^{4}	**OPS44**	1.9×10^{1}	2.0×10^{1}[b]
**OPS5**	3.8	2.9×10^{-1}	**OPS25**	9.1×10^{3}	1.8×10^{4}	**OPS45**	1.9×10^{1}	2.5×10^{1}
**OPS6**	1.1×10^{3}	1.3×10^{3}	**OPS26**	1.5×10^{1}	3.8×10^{-1}	**OPS46**	2.7×10^{1}	2.3
**OPS7**	3.9×10^{3}	1.6×10^{4}	**OPS27**	5.4×10^{3}	2.9×10^{4}	**OPS47**	7.7×10^{-1}	2.4×10^{-1}
**OPS8**	1.0×10^{4}	1.6×10^{4}	**OPS28**	7.0×10^{3}	3.4×10^{4}	**OPS48**	2.0×10^{3}	1.7×10^{3}
**OPS9**	6.7×10^{3}	6.8×10^{3}	**OPS29**	5.2×10^{3}	3.1×10^{4}	**OPS49**	4.3×10^{2}	1.8
**OPS10**	7.0×10^{3}	1.8×10^{4}	**OPS30**	4.9×10^{3}	2.9×10^{4}	**OPS50**	2.5×10^{4}	5.1×10^{3}
**OPS11**	7.6×10^{3}	1.6×10^{4}	**OPS31**	2.3×10^{3}	1.7×10^{4}	**OPS51**	3.0×10^{3}	8.2×10^{2}
**OPS12**	8.2×10^{3}	1.3×10^{4}	**OPS32**	3.0×10^{3}	9.3×10^{3}	**OPS52**	7.0×10^{1}	5.5×10^{4}
**OPS13**	8.1×10^{2}	3.2×10^{2}	**OPS33**	7.8×10^{2}	4.6×10^{3}	**OPS53**	1.2×10^{3}	4.7×10^{3}
**OPS14**	2.6×10^{3}	3.4×10^{2}	**OPS34**	6.0	1.9×10^{4}	**OPS54**	4.0×10^{3}	5.1×10^{3}
**OPS15**	2.7×10^{3}	2.0×10^{4}	**OPS35**	2.9	1.1×10^{4}	**OPS55**	1.4×10^{1}	2.0×10^{4}
**OPS16**	1.8×10^{3}	1.8×10^{3}	**OPS36**	7.6×10^{2}	1.1×10^{4}	**OPS56**	1.4×10^{1}	5.3[b]
**OPS17**	2.2×10^{3}	6.3×10^{3}	**OPS37**	1.0×10^{3}	6.5×10^{3}	**OPS57**	1.9×10^{3}	8.6×10^{3}
**OPS18**	1.6×10^{3}	1.5×10^{4}	**OPS38**	2.4×10^{3}	1.6×10^{4}	**OPS58**	7.9	6.7
**OPS19**	1.0×10^{3}	1.6×10^{4}	**OPS39**	4.3×10^{2}	3.3×10^{4}	**OPS59**	6.1×10^{3}[a]	2.3[b]
**OPS20**	1.9×10^{3}	2.1×10^{4}	**OPS40**	6.5	9.4	**OPS60**	1.1×10^{4}[a]	2.2×10^{-1}[b]

LT_t represents the excited-state lifetime measured in toluene, while **LT_d** represents that measured in DMSO. [a] MeCN or [b] DMF was used as solvents for the measurement.

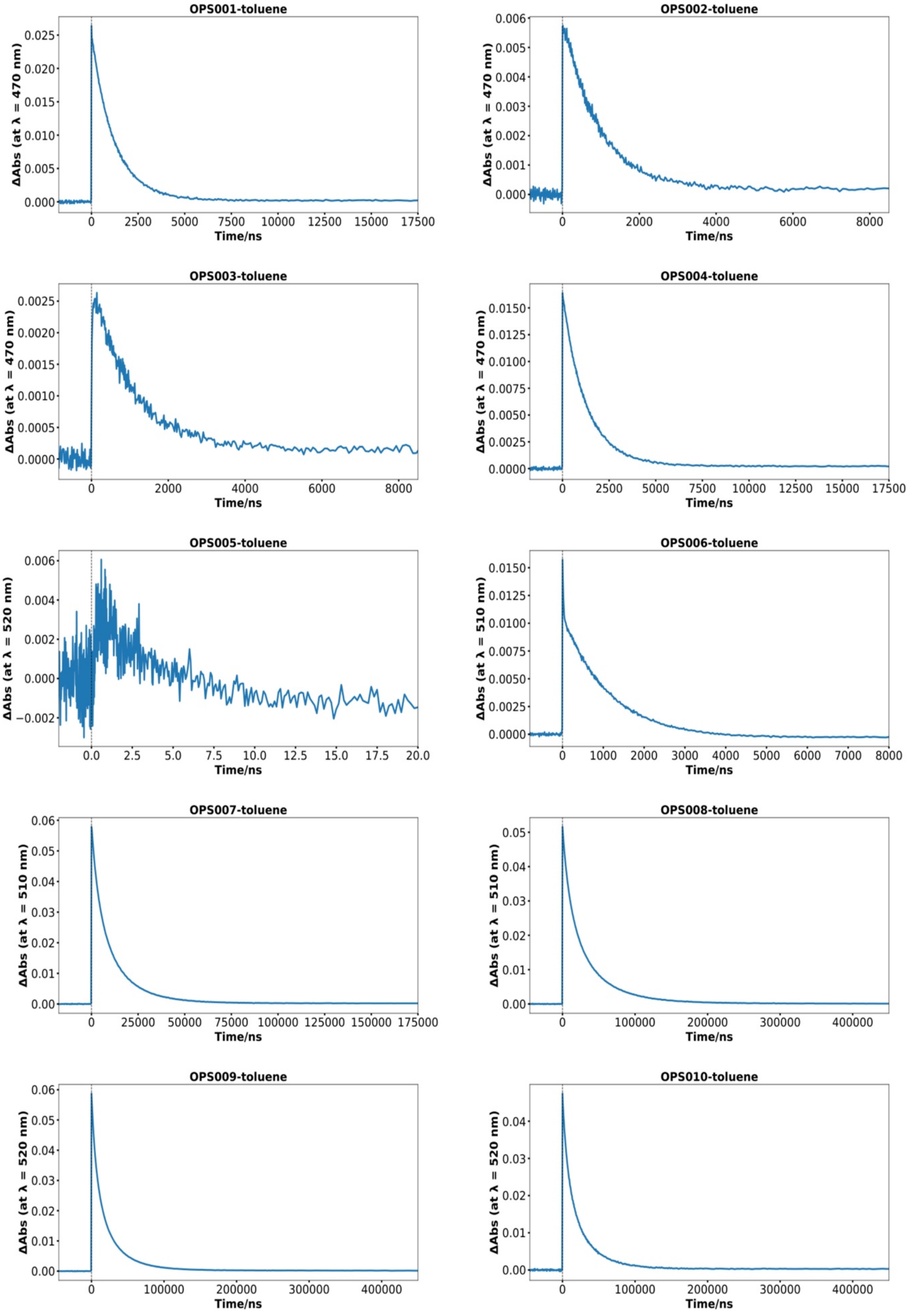

Figure S6-1. Decay time profiles of the transient absorption

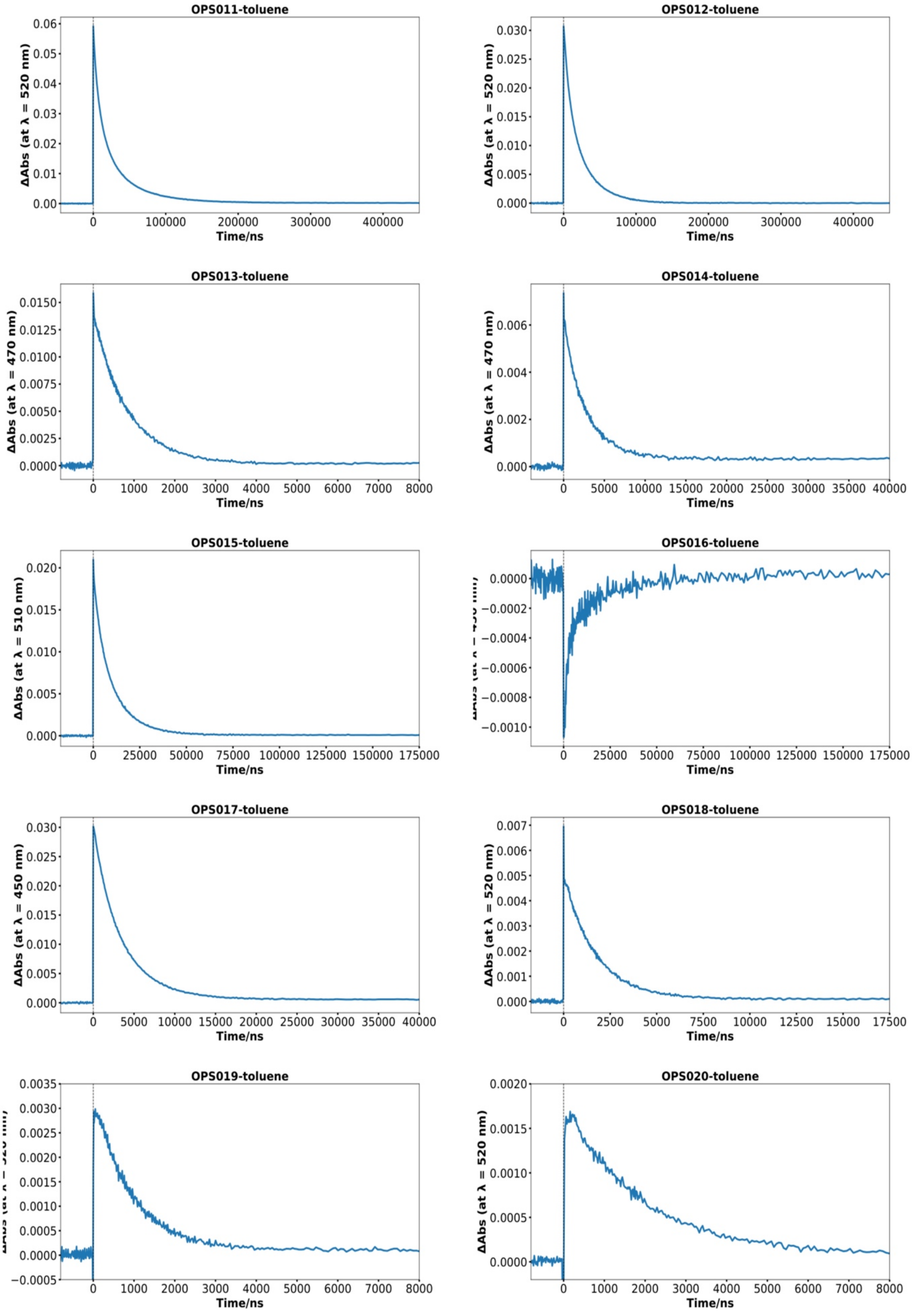

Figure S6-2. Decay time profiles of the transient absorption

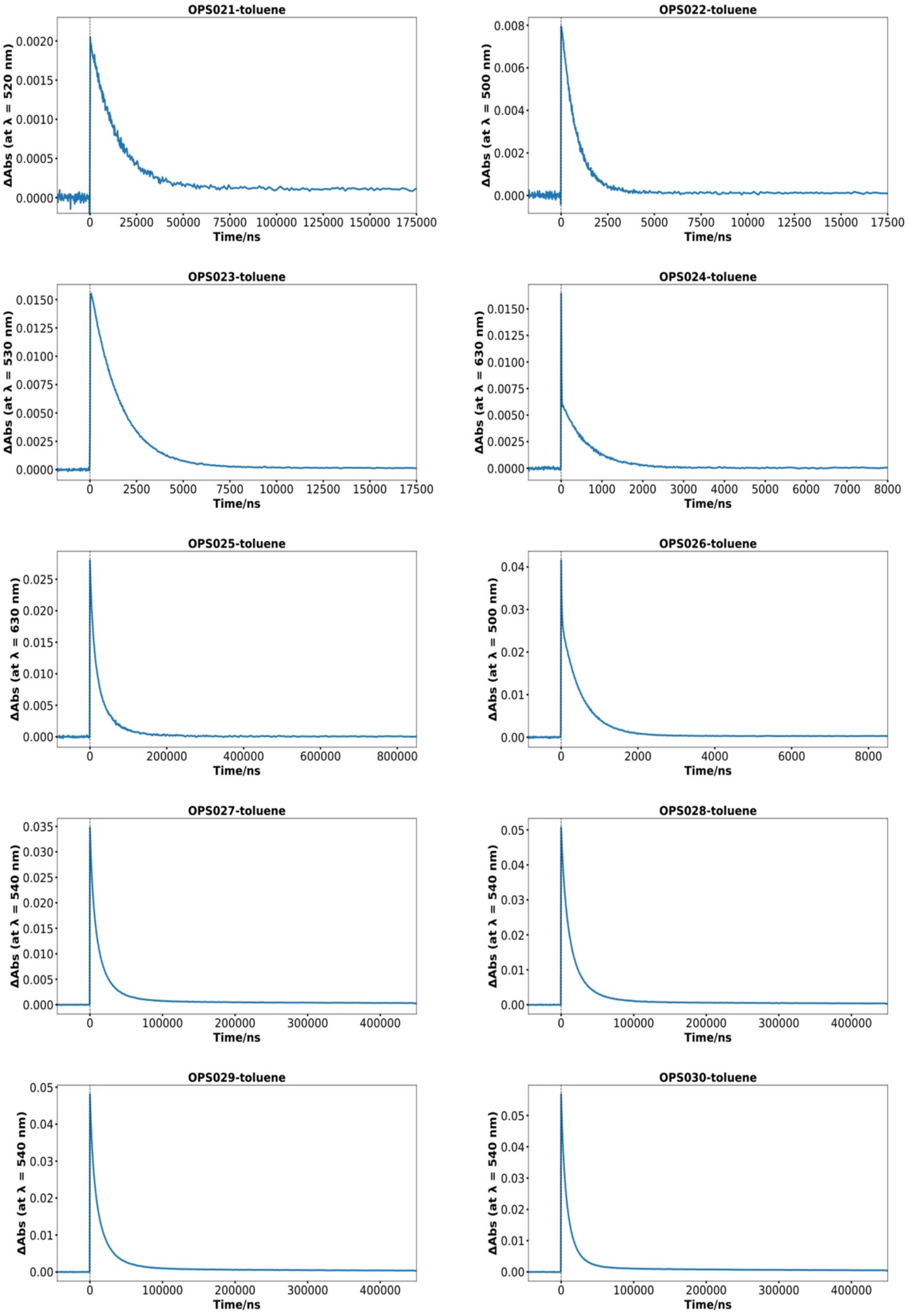

Figure S6-3. Decay time profiles of the transient absorption

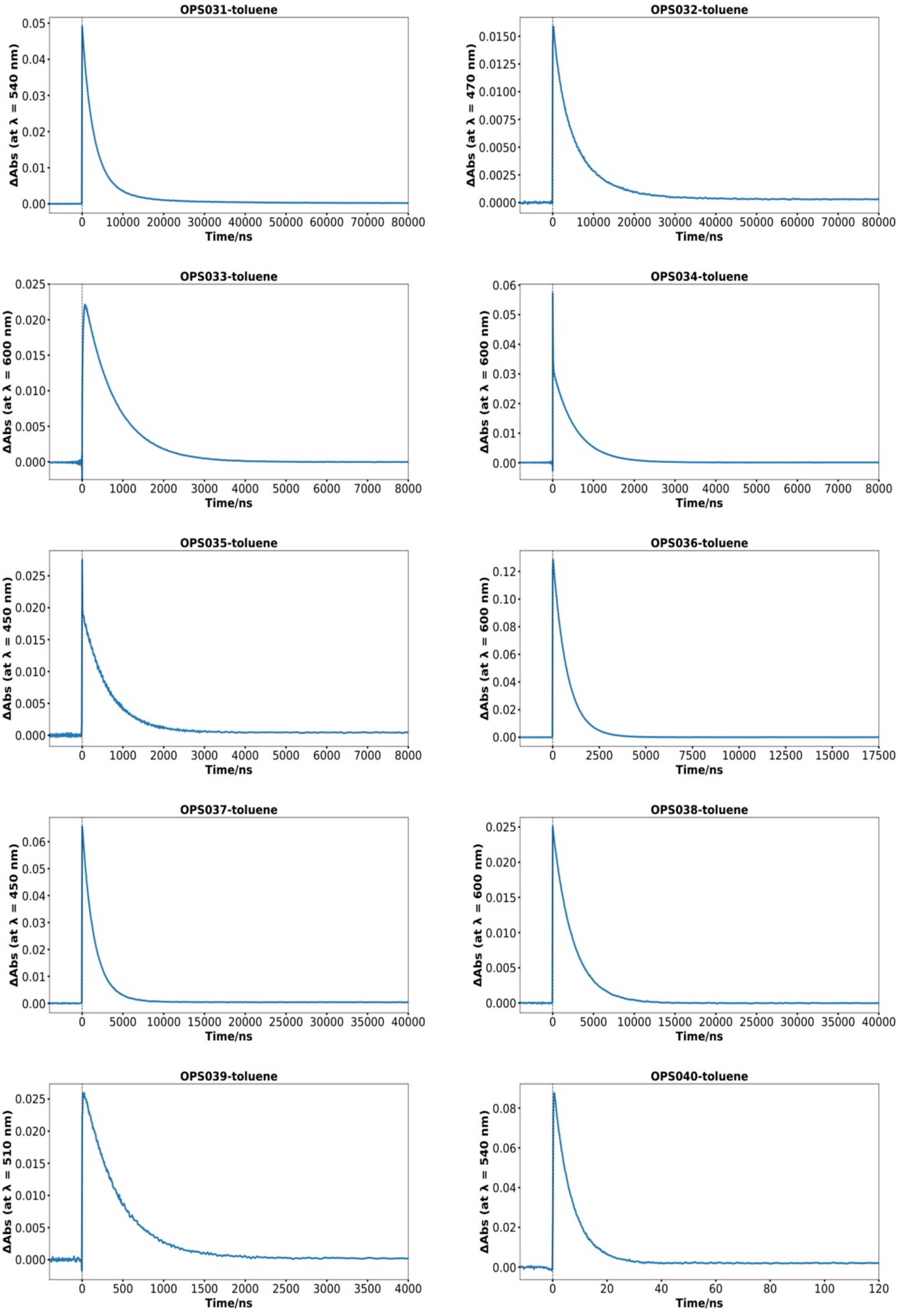

Figure S6-4. Decay time profiles of the transient absorption

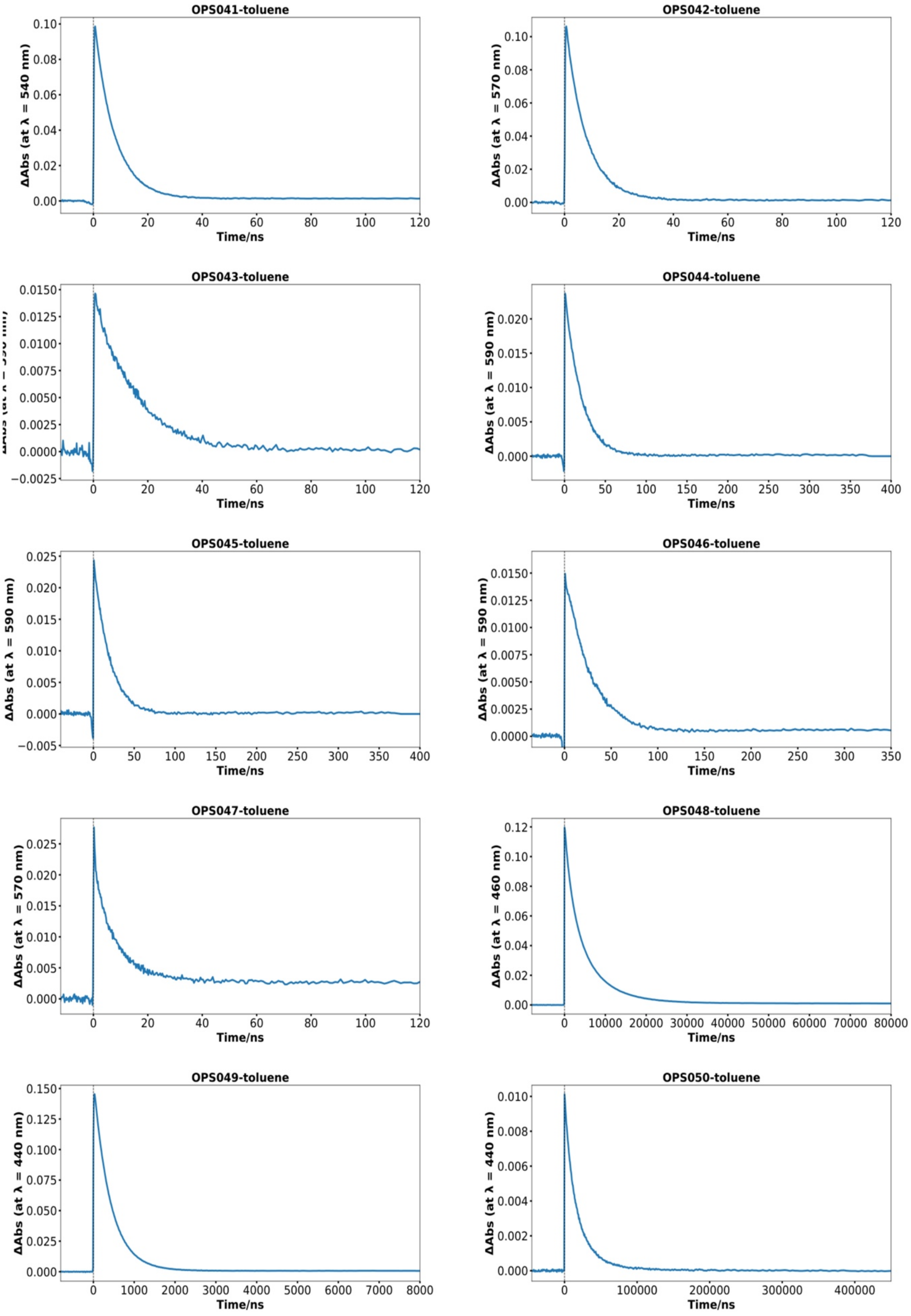

Figure S6-5. Decay time profiles of the transient absorption

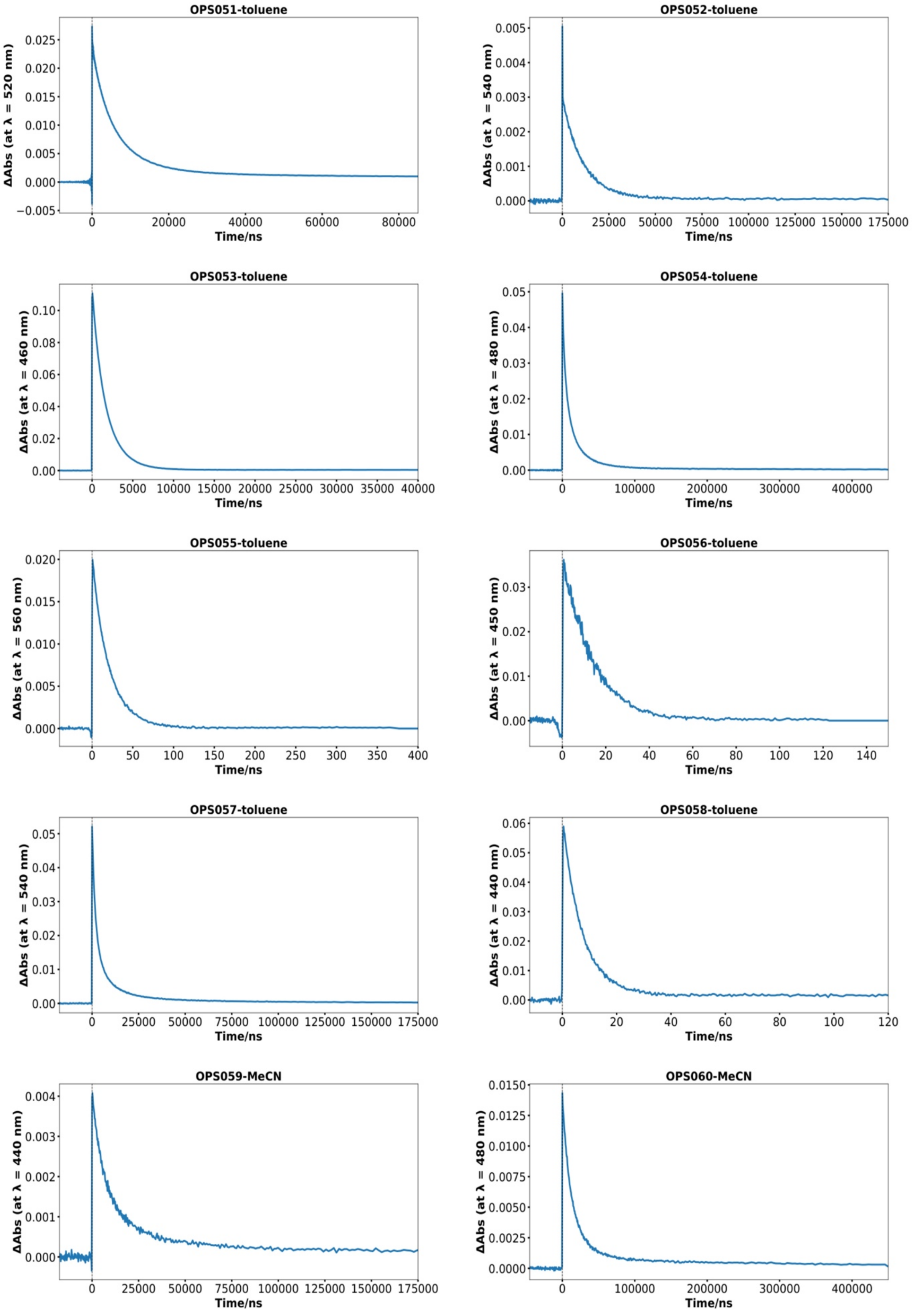

Figure S6-6. Decay time profiles of the transient absorption

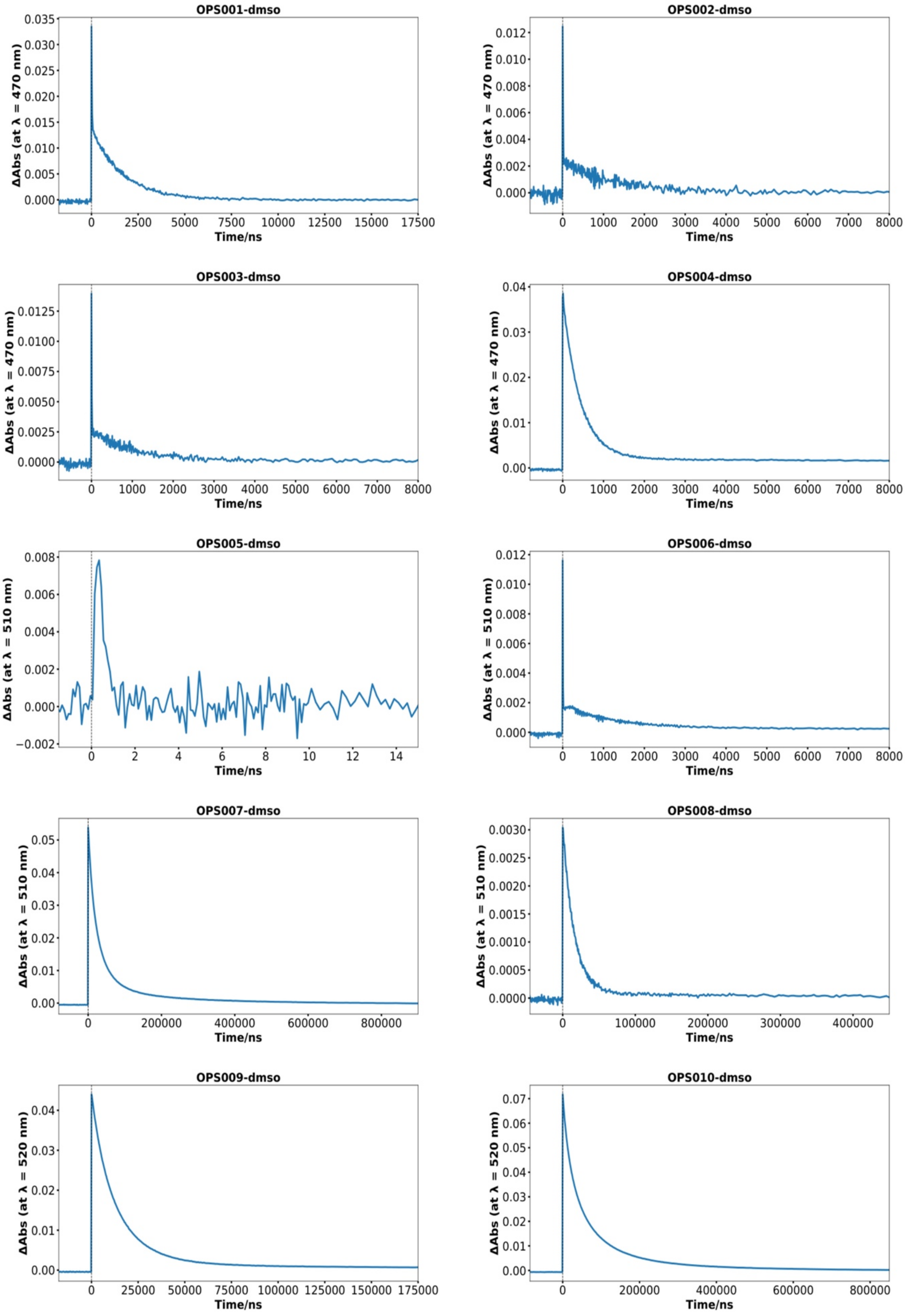

Figure S6-7. Decay time profiles of the transient absorption.

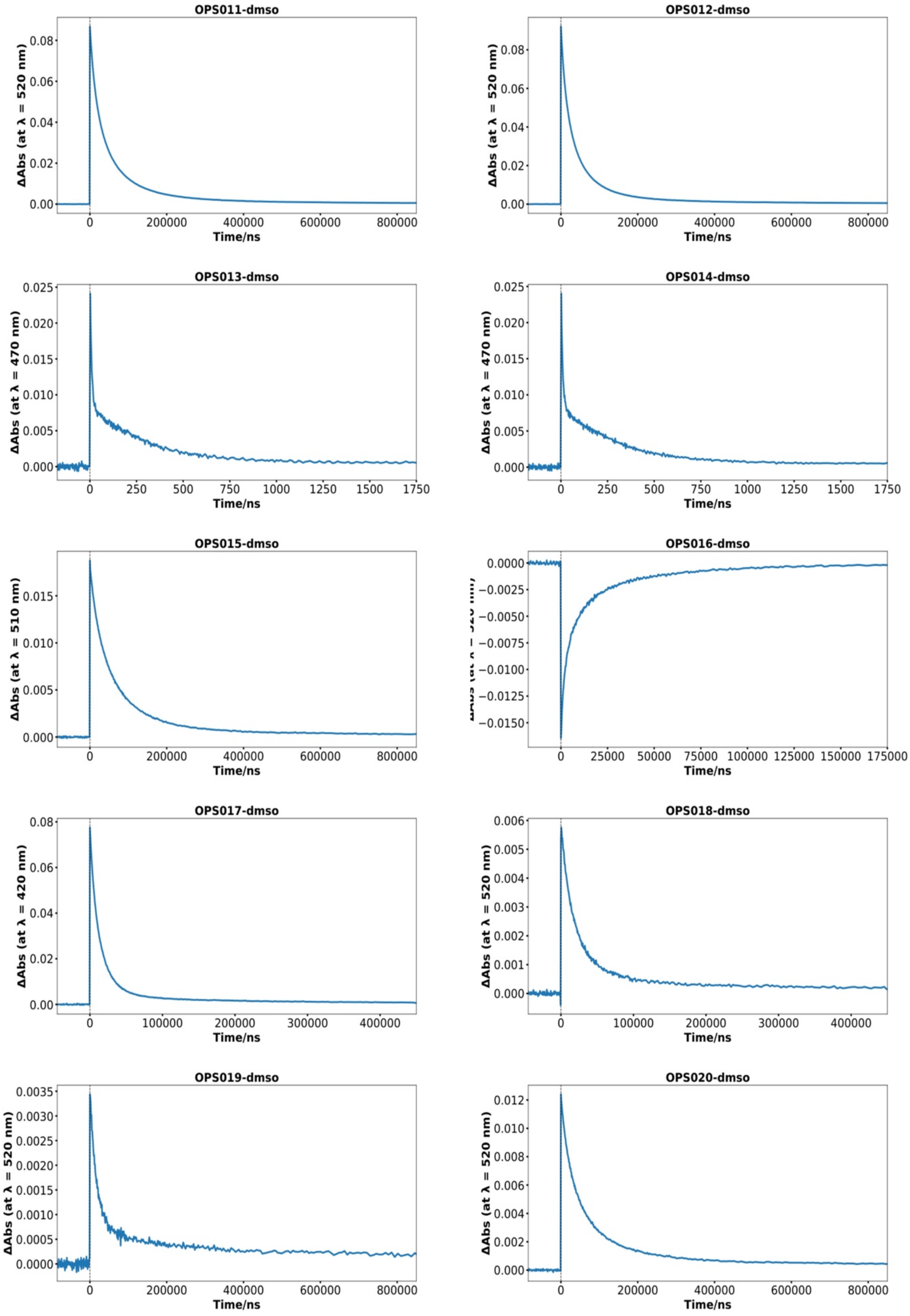

Figure S6-8. Decay time profiles of the transient absorption

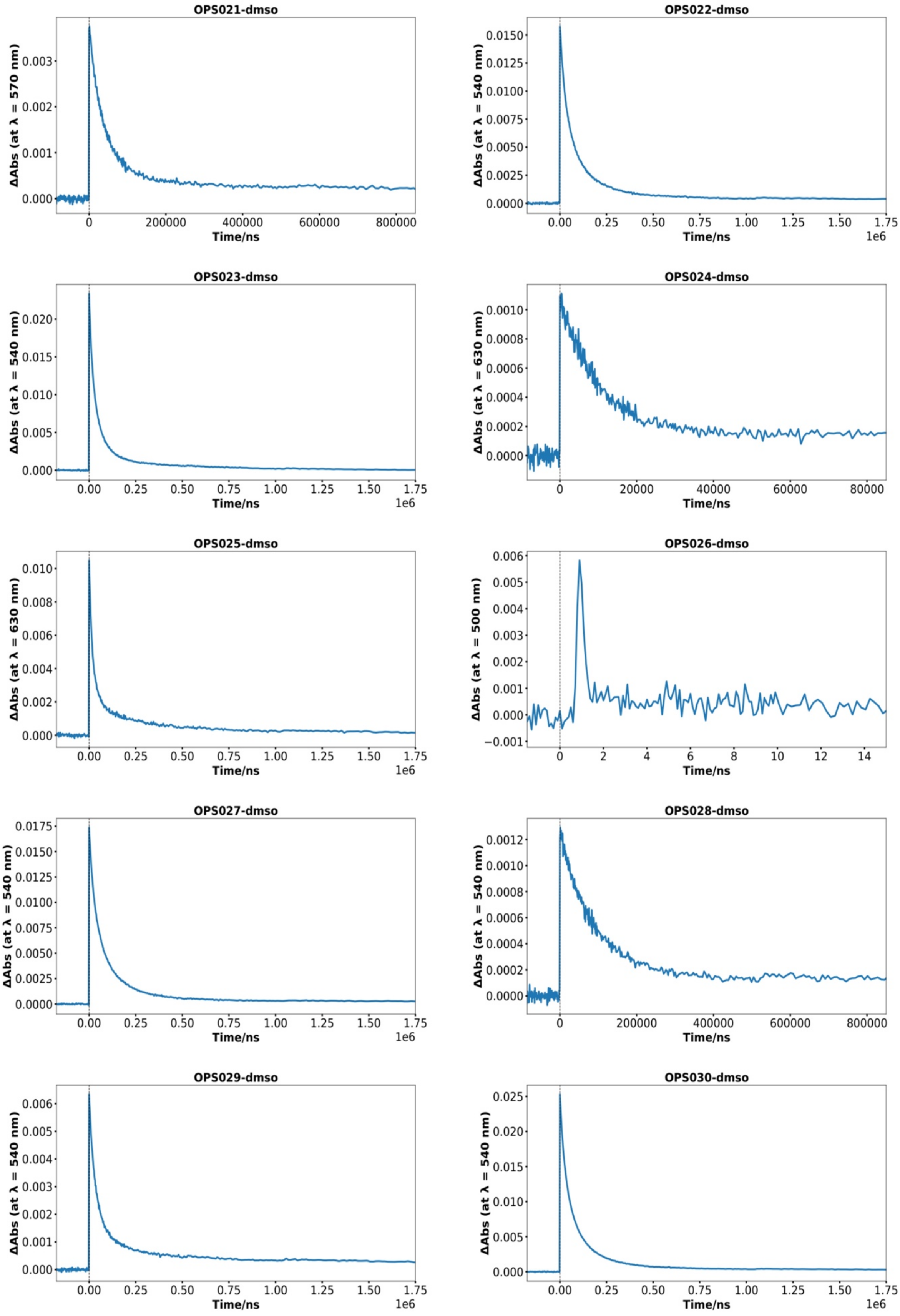

Figure S6-9. Decay time profiles of the transient absorption

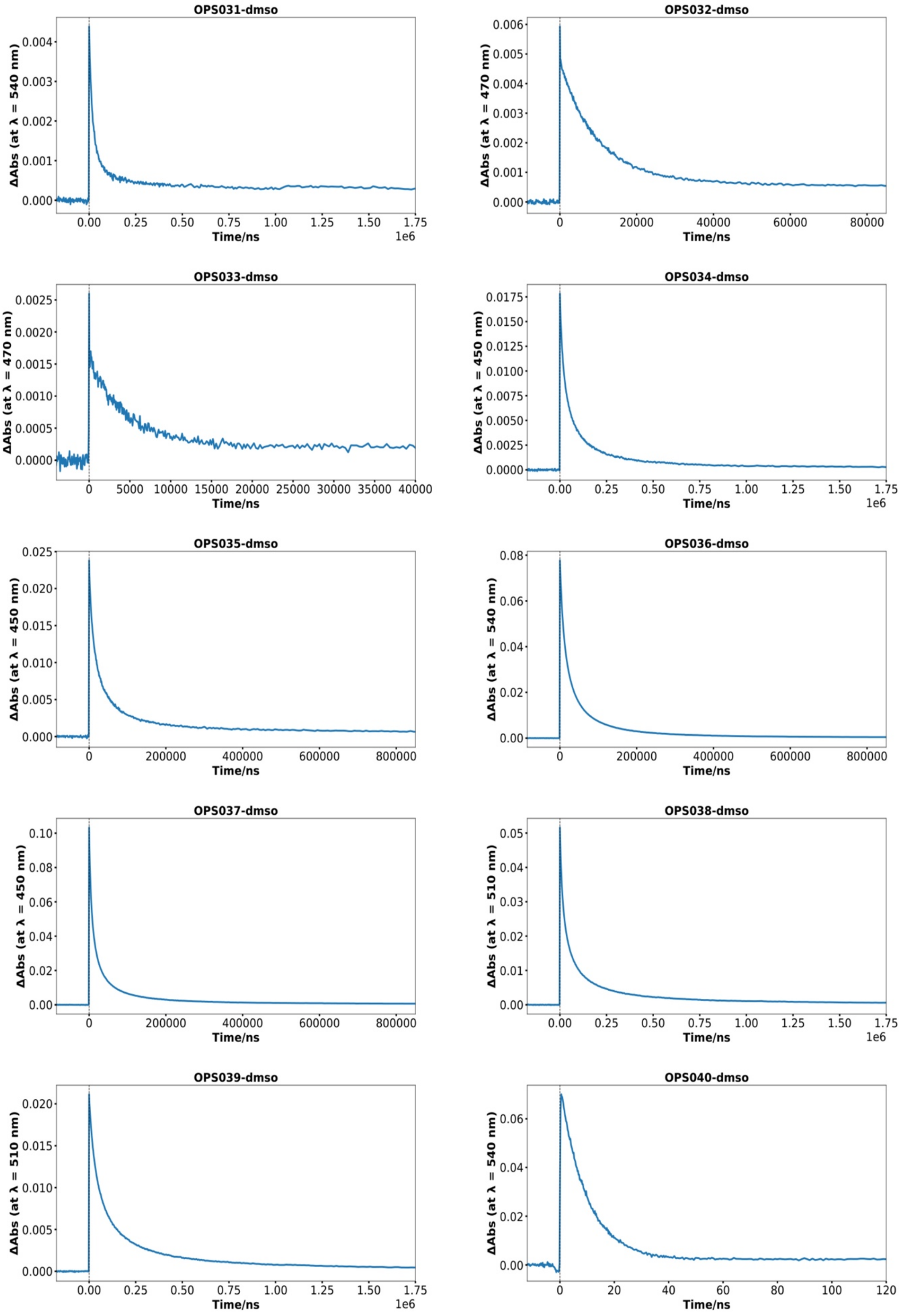

Figure S6-10. Decay time profiles of the transient absorption

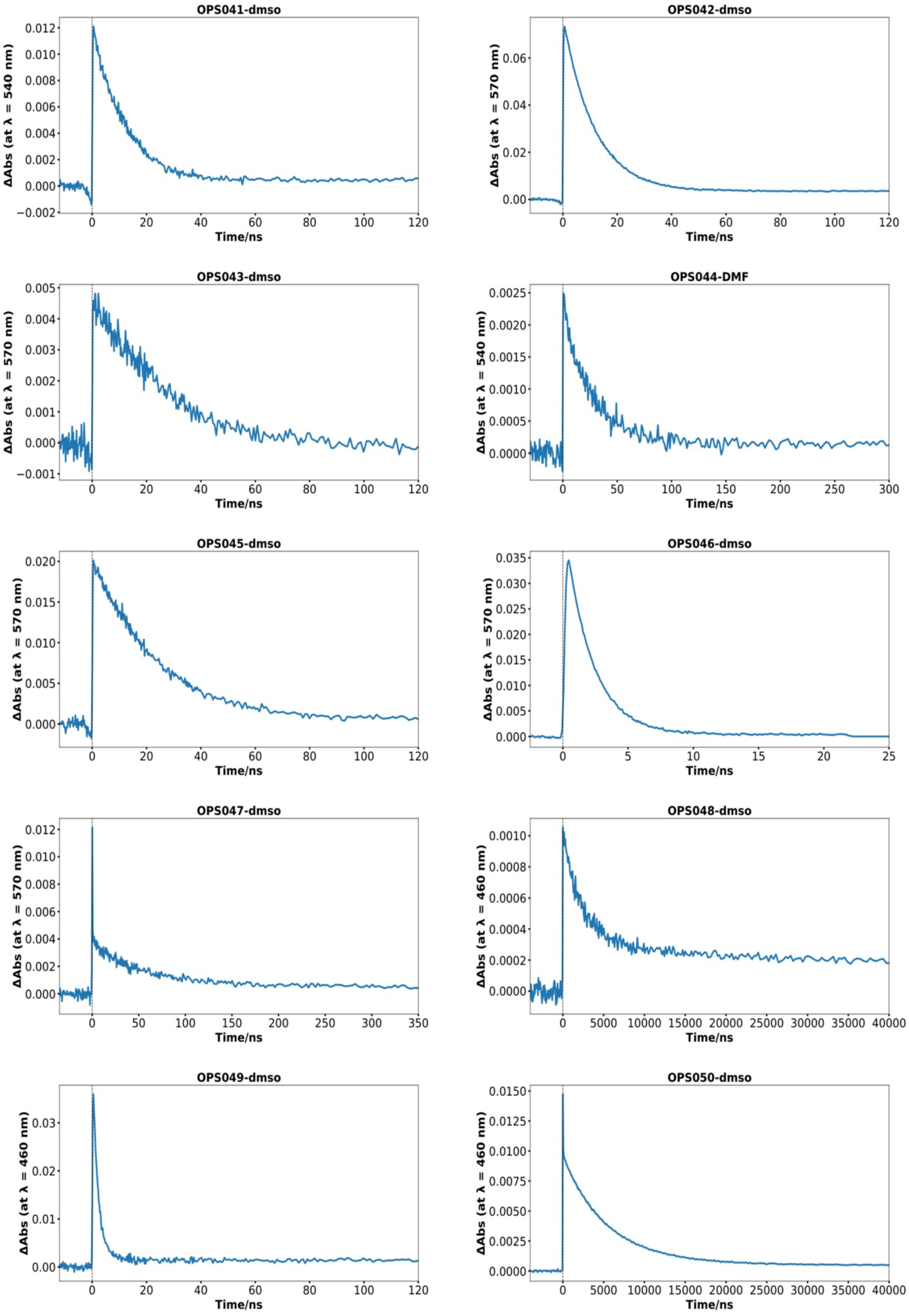

Figure S6-11. Decay time profiles of the transient absorption

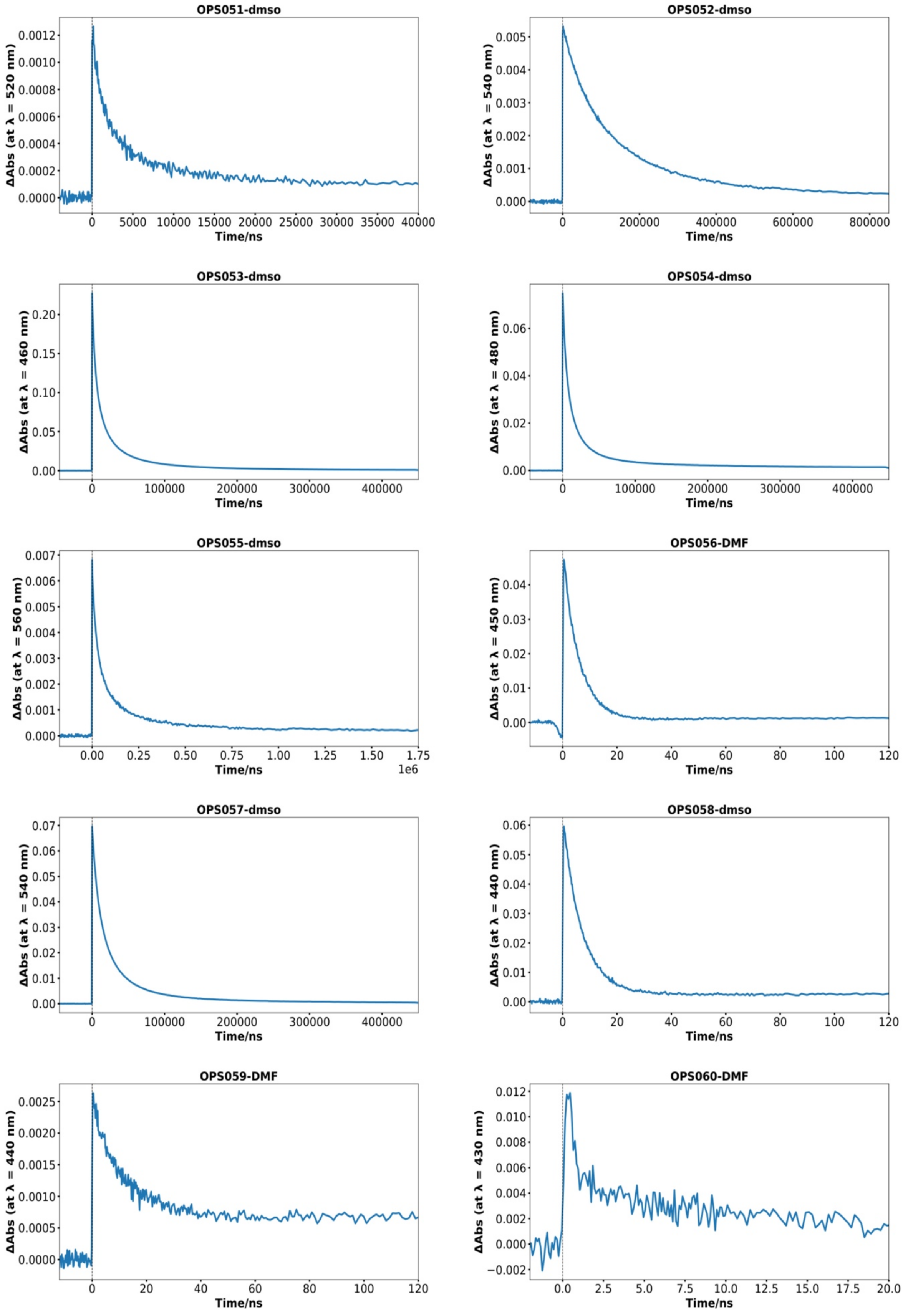

Figure S6-12. Decay time profiles of the transient absorption

Computational Details for the Design of Descriptors

(1) Quantum chemical calculations of physical properties: E_{HOMO}, E_{LUMO}, E_{S1}, f_{S1}, E_{T1}, ΔE_{ST}, and ΔDM

To generate a dataset based on physical properties of OPSs, we performed DFT and TD-DFT calculations at the CAM-B3LYP-D3BJ/6-31G(d) level of theory. To this end, we thoroughly used molecular geometries optimized in the singlet ground state (S_0). The solvation effect in toluene was treated using the Polarized Continuum Model (PCM), where the excited-state energies were computed based on the linear response scheme with equilibrium solvation. All quantum chemical calculations were carried out using the Gaussian 16 program package.[S10]

- E_{HOMO}, E_{LUMO}: HOMO and LUMO energy levels obtained through DFT calculation.
- E_{S1}: Total energy of the lowest-lying singlet excited state (S_1), which was determined by the single-point TD-DFT calculation (TD-CAM-B3LYP/6-31G(d) level).
- f_{S1}: Oscillator strength of vertical excitation between S_0 and S_1 states offered by the TD-DFT calculation.
- E_{T1}: Total energy of the lowest-lying triplet excited state (T_1), which was determined by the single-point DFT calculation with the unrestricted open-shell treatment.
- ΔE_{ST}: Vertical S_1–T_1 energy splitting, given as $\Delta E_{\mathrm{ST}} = E_{\mathrm{S1}} - E_{\mathrm{T1}}$.
- ΔDM: Difference in the predicted dipole moment (DM) between the S_0 and S_1 states, which is gauged using the following formula as also employed in our previous work:[S1]

$$\Delta\mathrm{DM} = \sqrt{\left(\mathrm{DM}_{x,\mathrm{e}} - \mathrm{DM}_{x,\mathrm{g}}\right)^2 + \left(\mathrm{DM}_{y,\mathrm{e}} - \mathrm{DM}_{y,\mathrm{g}}\right)^2 + \left(\mathrm{DM}_{z,\mathrm{e}} - \mathrm{DM}_{z,\mathrm{g}}\right)^2}$$

$\mathrm{DM}_{x,\mathrm{g}}$, $\mathrm{DM}_{y,\mathrm{g}}$, $\mathrm{DM}_{z,\mathrm{g}}$: Dipole moment (x-, y-, or z-coordinate) of OPSs in the S_0 state

$\mathrm{DM}_{x,\mathrm{e}}$, $\mathrm{DM}_{y,\mathrm{e}}$, $\mathrm{DM}_{z,\mathrm{e}}$: Dipole moment (x-, y-, or z-coordinate) of OPSs in the S_1 state at the TD-CAM-B3LYP/6-31G(d) level

(2) Decay rate constant simulation

Numerical simulation of the decay rate constants of electronic transitions was performed using the thermal vibration correlation function (TVCF) formalism[S11,S12] based on Fermi's golden rule for excited-state dynamics. The rate constant formulas of the TVCF theory were evaluated using molecular data obtained by the preceding quantum chemical (QC) calculations at the DFT level of theory.

In the QC calculations, we determined three optimized structures for each OPS compound for the S_0, S_1, and T_1 states, respectively. We employed the CAM-B3LYP-D3BJ/6-31G(d) level of theory, along with the PCM to account for the solvation effect in toluene. The T_1-optimized geometries were obtained using the unrestricted open-shell Kohn-Sham treatment, while the S_1-optimized geometries were determined through the TD-DFT approach. We calculated harmonic vibrational frequencies and their normal modes for these three states at the corresponding geometries. Nonadiabatic coupling (NAC) and transition dipole moments between the S_0 and S_1 states were computed in the process of the TD-DFT calculations at the S_1 geometry. We used the Gaussian 16 (C.01) program package for these QC calculations.

Given the QC data for the S_0, S_1, and T_1 states, we performed decay rate constant calculations using our in-house computer implementation of the TVCF theory.[S13,S14,S15] The following transition processes

were considered in the TVCF calculations to predict the decay rate constants:

- $k_r(S_1 \rightarrow S_0)$: Radiative decay rate constant from S_1 to S_0.
- $k_{ic}(S_1 \rightarrow S_0)$: Nonradiative decay rate constant via internal conversion (IC) from S_1 to S_0.
- $k_{isc}(S_1 \rightarrow T_1)$: Nonradiative decay rate constant via intersystem crossing (ISC) from S_1 to T_1.
- $k_{risc}(T_1 \rightarrow S_1)$: Rate constant of reverse intersystem crossing (RISC) from T_1 to S_1.
- $k_{isc}(T_1 \rightarrow S_0)$: Nonradiative decay rate constant via ISC from T_1 to S_0.

The formulas of radiative decay transition rate (k_r) and nonradiative ISC/RISC rate (k_{isc} or k_{risc}) were evaluated using transition dipole moments and spin-orbit coupling matrix elements (SOCMEs), respectively, as underlying coupling factors with the Franck–Condon (FC) treatment. The IC rate (k_{ic}) was simulated with the direct nonadiabatic transition term at the first-order perturbation. The redundant internal coordinate space was used for the normal coordinate construction. Our prior work can be referred for details on the theoretical aspects of our TVCF calculation.[S14,S15]

In the TVCF approach, the FC-level rate constant formula for the S_1-T_1 ISC process with the spin-sublevel $M\ (=-1,0,1)$ is written as

$$k_{\mathrm{isc}}^{M} = \frac{1}{\hbar^2 Z_{\mathrm{i}}} \left| H_{\mathrm{fi}}^{\mathrm{SO},M} \right|^2 \int dt\ e^{-i\frac{\Delta E_{\mathrm{ST}}}{\hbar}t} \rho^{\mathrm{FC}}(t,T) \qquad \text{(eq. S1)}$$

with the correlation function $\rho^{\mathrm{FC}}(t,T)$

$$\rho^{\mathrm{FC}}(t,T) = \mathrm{Tr}\left[\exp\left(-i\tau_{\mathrm{i}}\hat{H}_{\mathrm{i}}\right)\exp\left(-i\tau_{\mathrm{f}}\hat{H}_{\mathrm{f}}\right)\right] \qquad \text{(eq. S2)}$$

where Z_{i} and T are the partition function of the vibrational state in the initial state and temperature, respectively, $\tau_{\mathrm{i/f}}$ is the converted time t for the initial/final vibrational Hamiltonian $\hat{H}_{\mathrm{i/f}}$, and $H_{\mathrm{fi}}^{\mathrm{SO},M}$ represents the SOCME. The definitions of these entities are detailed in Ref. [S12] and Ref. [S14]. The $\rho^{\mathrm{FC}}(t,T)$ is readily evaluated at given t and T using the prepared QC data. As seen in eq. S1, k_{isc}^{M} forms as a function of ΔE_{ST}, for which the QC prediction should be used. We may take advantage of this direct dependence of k_{isc}^{M} on ΔE_{ST}; hence, in some cases, the experimental ΔE_{ST} can be used in place of the QC prediction in evaluating k_{isc}^{M}. This scheme is beneficial when it is challenging to obtain a reliable prediction of ΔE_{ST} using the DFT-based approach.[S15,S16] Indeed, we exploited this strategy to achieve a more reliable prediction of $k_{isc}(S_1 \rightarrow T_1)$ and $k_{risc}(T_1 \rightarrow S_1)$ by employing experimental ΔE_{ST}.

The SOCMEs required for the k_{isc} and k_{risc} simulations as inputs were obtained using the ORCA (version 5.0.3)[S17,S18] program package. The CPCM(toluene)-ZORA-TD-CAM-B3LYP/ZORA-def2-SV(P) level[S19–S21] was employed for DFT-based SOCME calculations. As acceleration techniques, we used the density fitting to the Coulomb term with the SARC/J basis[S22] and the chain-of-spheres exchange method for the exchange term, referred as RIJCOSX method.[S23,S24] For the SOC operator, a Breit-Pauli type Hamiltonian with a mean-field approximation for the 2-electron term[S25] was used. Solvation effect on the excited states were computed based on the linear response in the nonequilibrium regime.

(3) Design of scaled descriptors

RC consists of five descriptors, i.e., ($k_r(S_1 \rightarrow S_0)$, $k_{ic}(S_1 \rightarrow S_0)$, $k_{isc}(S_1 \rightarrow T_1)$, $k_{risc}(T_1 \rightarrow S_1)$, and $k_{isc}(T_1 \rightarrow S_0)$). **s_RC** is defined as the ratio of each rate constant to the sum of all five rate constants, as given by the following equation:

$$s\_k_r(S_1 \rightarrow S_0) = \frac{k_r(S_1 \rightarrow S_0)}{k_r(S_1 \rightarrow S_0) + k_{ic}(S_1 \rightarrow S_0) + k_{isc}(S_1 \rightarrow T_1) + k_{risc}(T_1 \rightarrow S_1) + k_{isc}(T_1 \rightarrow S_0)}$$

For example, in the case of **OPS1**, the values of each rate constant are 2.0×10^7, 4.4×10^2, 2.6×10^7, 5.3×10^1, and 3.8×10^0, respectively, so s_$k_r(S_1 \rightarrow S_0)$ can be calculated as follows:

$$s\_k_r(S_1 \rightarrow S_0) = \frac{2.0 \times 10^7}{2.0 \times 10^7 + 4.4 \times 10^2 + 2.6 \times 10^7 + 5.3 \times 10^1 + 3.8 \times 10^0)} = 4.3 \times 10^{-1}$$

Results of Machine Learning

(1) General information for ML

Python (3.10.14) was used as a language for this research, and used packages were matplotlib (3.9.0), numpy (1.26.4), pandas (2.2.2), shap (0.46.0), and scikit-learn (1.4.2).

For the code and used datasets, see: https://github.com/Naoki-Noto/P6-20240509-RK

(2) Comparison of ML models

Table S13. Comparison of ML models

Algorithm	HGB	RF	SVM	LASSO
R^2_test	0.83	0.78	0.69	0.49
(sd)	(0.04)	(0.07)	(0.07)	(0.06)
RMSE_test	11.8	13.5	16.1	20.8
(sd)	(1.3)	(2.1)	(2.1)	(1.9)

The descriptors used were **s_RC** + 'E_{HOMO}, f_{S1}, ΔE_{ST}, and ΔDM'.

The R^2 and RMSE scores are averaged over 10 runs, with standard deviations in parentheses.

Table S14. Explored hyperparameters

Algorithm	Hyperparameters
Lasso	'alphas': [0.001, 0.01, 0.1, 1.0, 10.0], 'max_iter': [1000, 5000, 10000], 'tol': [1e-4, 1e-3, 1e-2].
SVM	'C': [1e+02, 1e+03 1e+04], 'gamma': [1e-03, 1e-02, 1e-01, 1], 'gamma': [0.1, 0.5, 1]
RF	'max_depth': [4, 6, 8], 'n_estimators': [100, 1000, 5000]
HGB	'max_depth': [6, 7, 8], 'l2_regularization': [100, 1000, 5000], 'min_sample_leaf': [3, 5, 10]

(3) Comparison of descriptor performance derived from the difference in methods of DFT calculation

The impact of three different calculation methods on the predictive performance was investigated. The PCM(toluene)-PBE1PBE-D3/6-31G(d) (denoted as PBE0_tol), PCM(toluene)-CAM-B3LYP-D3/6-31G(d) (denoted as CAM_tol), and PCM(DMSO)-TD-CAM-B3LYP-D3/6-31G(d) (denoted as CAM_dmso) levels were tested.

Table S15. Comparison of rate constant-based descriptor sets in the C–O bond formation

Method	PBE0_tol		CAM_tol		CAM_dmso	
Descriptor	**RC**	**s_RC**	**RC**	**s_RC**	**RC**	**s_RC**
R^2_test	0.78	0.77	0.79	0.78	0.78	0.75
(sd)	(0.05)	(0.05)	(0.05)	(0.04)	(0.04)	(0.04)
RMSE_test	13.5	13.8	13.1	13.5	13.4	14.3
(sd)	(1.3)	(0.9)	(1.3)	(1.1)	(1.2)	(0.7)

R^2 and RMSE scores are averaged over 10 runs, with standard deviations in parentheses.

Table S16. Performance of rate constant-based descriptors calculated at the PCM(DMSO)-CAM-B3LYP/6-31G(d) level in the radical addition

Descriptor	**RC**	**s_RC**	**RC**+'E_{HOMO}, E_{LUMO}, f_{S1}, ΔE_{ST}, ΔDM'
R^2_test	0.71	0.65	0.78
(sd)	(0.06)	(0.04)	(0.03)
RMSE_test	18.1	19.8	15.6
(sd)	(2.1)	(1.7)	(1.4)

R^2 and RMSE scores are averaged over 10 runs, with standard deviations in parentheses.

(4) Relationship between physical properties and catalytic activities of OPSs

Table S17. Summary of catalytic properties.

**OPS**	E_{HOMO} (eV)	E_{LUMO} (eV)	ΔE_{ST} (eV)	Yield (%) **CO-a**	Yield (%) **CO-b**	Yield (%) **CO-c**	Yield (%) **CO-d**	Yield (%) **CO-e**
**OPS1**	-6.9384	-1.3187	0.3173	62	92	69	50	41
**OPS7**	-6.6426	-0.9578	0.4691	88	88	67	47	63
**OPS38**	-6.2067	-0.1317	0.1962	10	69	42	13	14
**OPS40**	-6.1648	-0.0509	0.7597	0	38	18	6	24
**OPS49**	-5.5680	0.0457	0.3045	0	3	2	8	0
**OPS56**	-7.3273	-1.9323	1.2445	0	5	23	11	2
**OPS58**	-6.4208	-1.2398	1.1812	0	0	2	0	0
**OPS59**	-9.3820	-3.4945	0.6213	0	0	2	0	0
**OPS60**	-9.0977	-3.4450	0.8680	0	0	0	0	0

E_{HOMO}, E_{LUMO}, and ΔE_{ST} values for the selected photosensitizers were obtained using calculations at the PCM(toluene)-CAM-B3LYP-D3/6-31G(d) level.

(5) SHAP waterfall plots including more detailed information

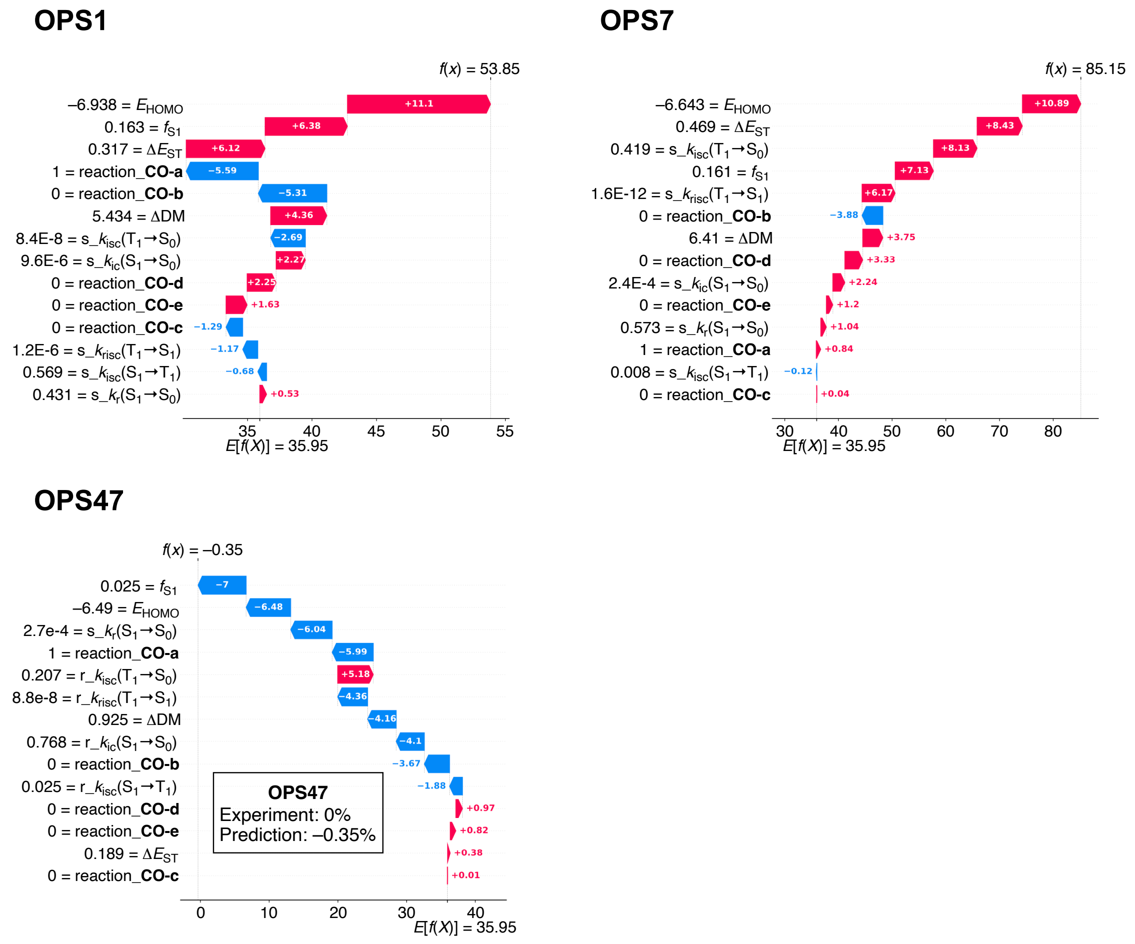

Figure S7. SHAP waterfall plots in the C–O bond formation

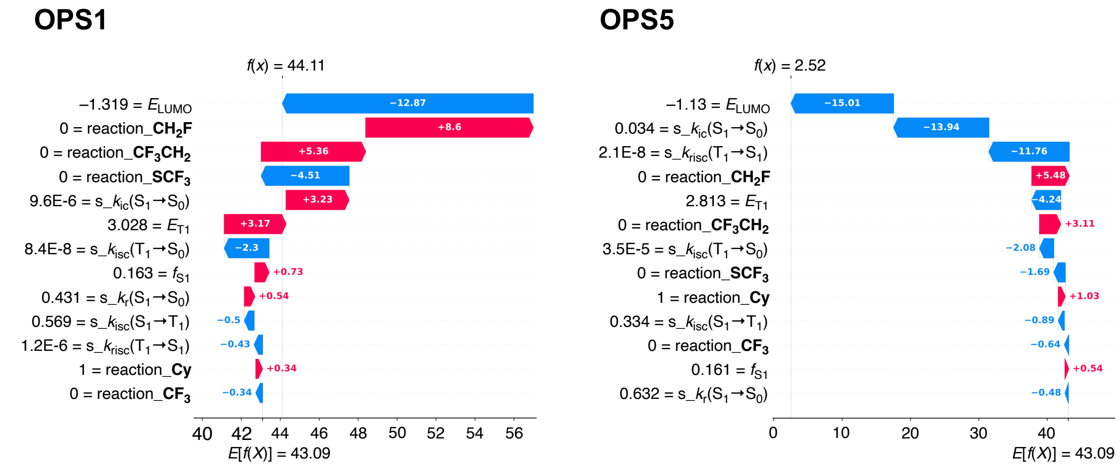

Figure S8. SHAP waterfall plots in the radical addition

Description of SHAP Plots

(1) SHAP summary plot / bar plot

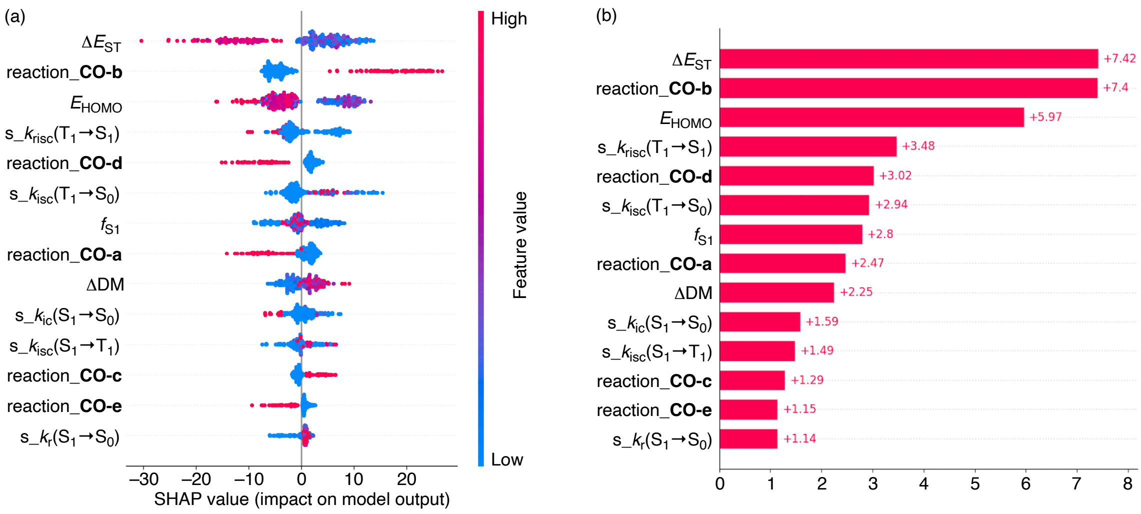

Figure S9. Examples of (a) SHAP summary and (b) bar plots

The SHAP summary plot (Figure S9a) provides a comprehensive overview of the overall trends in feature contributions. The display conventions for this plot are as follows:

- Each descriptor is listed along the vertical axis. Descriptors with higher mean absolute SHAP values are positioned toward the top of the plot, reflecting their greater overall influence on the model's predictions.
- The horizontal axis represents the SHAP values, which quantify both the magnitude and direction of each descriptor's influence on the output. In this plot, data points located on the right-hand side correspond to positive SHAP values, indicating that the respective descriptors contribute positively to the prediction. In contrast, data points positioned on the left-hand side correspond to negative SHAP values, indicating a negative contribution to the predicted outcome.
- Data points with high descriptor values are shown in red, those with low values appear in blue, and those with intermediate values are depicted in purple.

The SHAP bar plot (Figure S9b) focuses specifically on the quantitative magnitude of feature contributions. Each bar represents the mean absolute SHAP value of a descriptor, which is also shown numerically beside the bar. Similar to the summary plot, descriptors with higher mean absolute SHAP values are positioned toward the top. While this plot effectively highlights the relative importance of descriptors in determining the model output, it does not convey whether their influence is positive or negative.

For instance (Figure S9), ΔE_{ST} is the descriptor with the largest contribution, showing a mean absolute SHAP value of +7.42. Samples with high ΔE_{ST} values tend to exhibit negative SHAP values, whereas those with low ΔE_{ST} values tend to show large positive SHAP values. In contrast, s_k_r(S_1→S_0) provides the smallest contribution, with a mean absolute SHAP value of +1.14.

(2) SHAP scatter plot

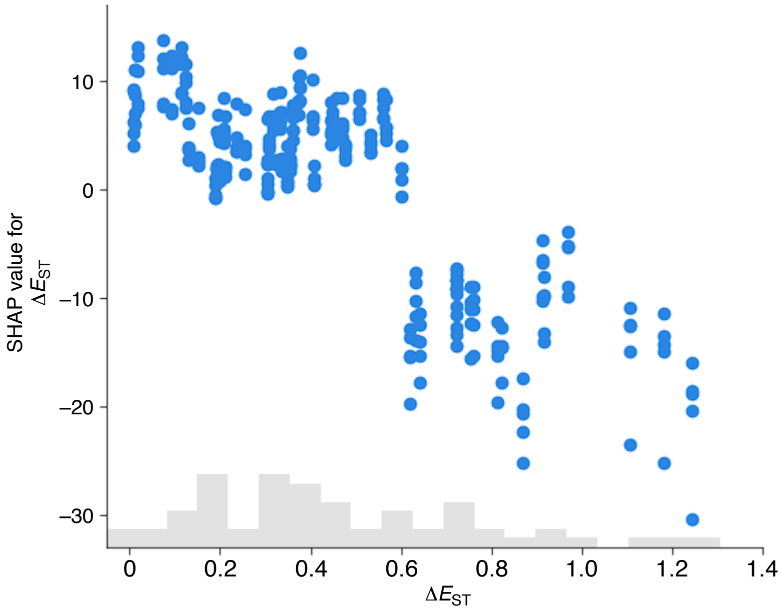

Figure S10. Example of a SHAP scatter plot

The SHAP scatter plot (Figure S10) provides the SHAP values of a specific descriptor. The display conventions for this plot are as follows:

- The horizontal axis represents the actual values of each descriptor.
- The vertical axis shows the corresponding SHAP values for each sample.
- A histogram is overlaid in the background, allowing the data distribution of each descriptor to be visualized.

In Figure S10, when ΔE_{ST} is smaller than c.a. 0.6, it tends to exhibit positive SHAP values, whereas when it is larger than c.a. 0.6, it shows a tendency to result in high, negative SHAP values. Compared with the summary plot, the scatter plot provides more detailed information on the specific ranges in which each descriptor influences the prediction and the manner of that influence.

(3) SHAP waterfall plot

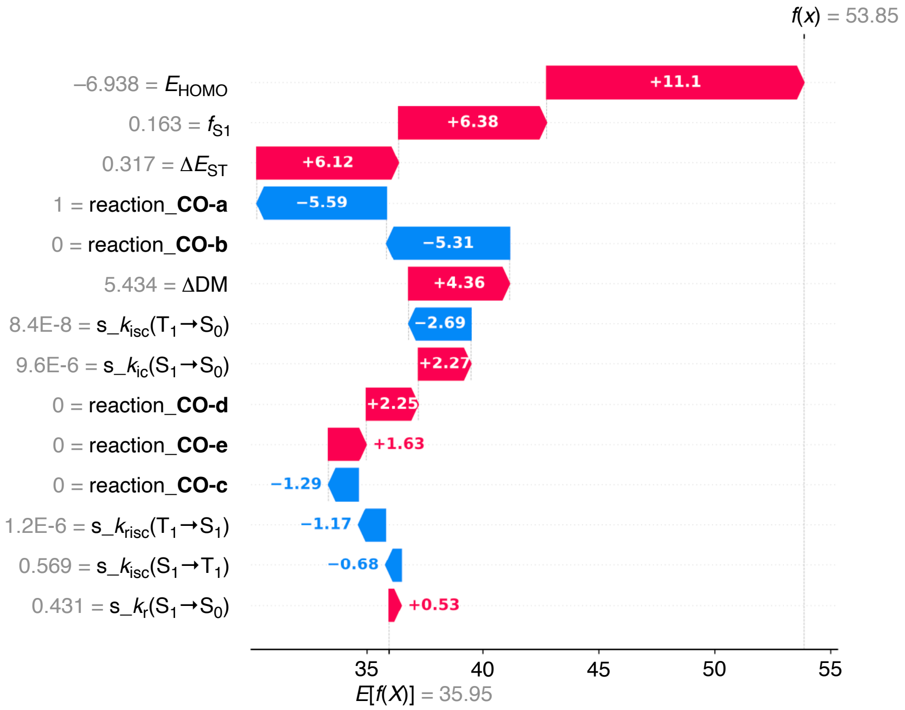

Figure S11. Example of a SHAP waterfall plot

The SHAP waterfall plot (Figure S11) provides detailed information on the feature contributions to an individual prediction. The display conventions for this plot are as follows:

- The lower baseline labeled $E[f(X)]$ represents the model's average prediction value, whereas $f(x)$ at the top indicates the predicted value for the specific sample under analysis, thus illustrating how the final prediction is derived from the global mean through the additive contributions of individual descriptors.
- Descriptors with larger absolute SHAP values are placed higher in the plot, where positive contributions are shown in red and negative contributions in blue, along with their corresponding SHAP values.
- Each descriptor is accompanied by its corresponding value. (In the main text, these values are omitted due to space limitations.)

In the displayed example (Figure S11), the five descriptors with the largest contributions are E_{HOMO}, f_{S1}, ΔE_{ST}, reaction_**CO-a**, and reaction_**CO-b**. In this sample, the actual values of E_{HOMO}, f_{S1}, ΔE_{ST}, reaction_**CO-a**, and reaction_**CO-b** are –6.938, 0.163, 0.317, 1, and 0, respectively. Among them, E_{HOMO}, f_{S1}, and ΔE_{ST} show positive contributions, with SHAP values of +11.1, +6.38, and +6.12, respectively. In contrast, reaction_**CO-a** and reaction_**CO-b** exhibit negative contributions, with SHAP values of –5.59 and –5.31, respectively. Consequently, the resulting predicted value (53.85) exceeds the model's average prediction (35.95) due to the cumulative effect of several descriptors with high, positive SHAP values.

References

[S1] N. Noto, R. Kunisada, T. Rohlfs, M. Hayashi, R. Kojima, O. García Mancheño, T. Yanai and S. Saito, *Nat. Commun.*, 2025, **16**, 3388.

[S2] L.-H. Wu, K. Zhao, Z.-L. Shen and T.-P. Loh, *Org. Chem. Front.*, 2017, **4**, 1872–1875.

[S3] S. Ni, Y. Zhang, C. Xie, H. Mei, J. Han and Y. Pan, *Org. Lett.*, 2015, **17**, 5524–5527.

[S4] R. Honeker, R. A. Garza-Sanchez, M. N. Hopkinson and F. Glorius, *Chem. – Eur. J.*, 2016, **22**, 4395–4399.

[S5] (a) H. Uoyama, K. Goushi, K. Shizu, H. Nomura and C. Adachi, *Nature*, 2012, **492**, 234–238. (b) M. Y. Wong and E. Zysman-Colman, *Adv. Mater.*, 2017, **29**, 1605444. (c) Z. Yang, Z. Mao, Z. Xie, Y. Zhang, S. Liu, J. Zhao, J. Xu, Z. Chi and M. P. Aldred, *Chem. Soc. Rev.*, 2017, **46**, 915–1016. (d) E. Speckmeier, T. G. Fischer and K. Zeitler, *J. Am. Chem. Soc.*, 2018, **140**, 15353–15365.

[S6] J. J. Snellenburg, S. Laptenok, R. Seger, K. M. Mullen and I. H. M. Van Stokkum, *J. Stat. Softw.*, 2012, **49**, 1–22

[S7] Y. Matsui, Y. Yokoyama, T. Ogaki, K. Ishiharaguchi, A. Niwa, E. Ohta, M. Saigo, K. Miyata, K. Onda, H. Naito and H. Ikeda, *J. Mater. Chem. C*, 2022, **10**, 4607–4613.

[S8] T. Nakagawa, K. Okamoto, H. Hanada and R. Katoh, *Opt. Lett.*, 2016, **41**, 1498–1501.

[S9] R. Ishimatsu, S. Matsunami, K.Shizu, C. Adachi, K. Nakano and T. Imato, *J. Phys. Chem. A*, 2013, **117**, 5607–5612.

[S10] M. J. Frisch, G. W. Trucks, H. B. Schlegel, G. E. Scuseria, M. A. Robb, J. R. Cheeseman, G. Scalmani, V. Barone, G. A. Petersson, H. Nakatsuji, X. Li, M. Caricato, A. V. Marenich, J. Bloino, B. G. Janesko, R. Gomperts, B. Mennucci, H. P. Hratchian, J. V. Ortiz, A. F. Izmaylov, J. L. Sonnenberg, D. Williams-Young, F. Ding, F. Lipparini, F. Egidi, J. Goings, B. Peng, A. Petrone, T. Henderson, D. Ranasinghe, V. G. Zakrzewski, J. Gao, N. Rega, G. Zheng, W. Liang, M. Hada, M. Ehara, K. Toyota, R. Fukuda, J. Hasegawa, M. Ishida, T. Nakajima, Y. Honda, O. Kitao, H. Nakai, T. Vreven, K. Throssell, J. A. Montgomery, Jr., J. E. Peralta, F. Ogliaro, M. J. Bearpark, J. J. Heyd, E. N. Brothers, K. N. Kudin, V. N. Staroverov, T. A. Keith, R. Kobayashi, J. Normand, K. Raghavachari, A. P. Rendell, J. C. Burant, S. S. Iyengar, J. Tomasi, M. Cossi, J. M. Millam, M. Klene, C. Adamo, R. Cammi, J. W. Ochterski, R. L. Martin, K. Morokuma, O. Farkas, J. B. Foresman, D. J. Fox, Gaussian 16, revision B.01 (Gaussian Inc., 2016).

[S11] Y. Niu, Q. Peng, C. Deng, X. Gao and Z. Shuai, *J. Phys. Chem. A*, 2010 **114**, 7817–7831.

[S12] Q. Peng, Y. Niu, Q. Shi, X. Gao and Z. Shuai, *J. Chem. Theory Comput.*, 2013, **9**, 1132–1143.

[S13] A. Takiguchi, N. Inai, S. Kang, M. Hagai, S. Lee, T. Yanai, D. Kim and H. Shinokubo, *Chem. Commun.*, 2022, **58**, 5956–5959.

[S14] N. Inai, S. Yamaguchi and T. Yanai, *ACS Phys. Chem Au*, 2023, **3**, 540–552.

[S15] M. Hagai, N. Inai, T. Yasuda, K. J. Fujimoto, and T. Yanai, *Sci. Adv.*, 2024, **10**, eadk3219.

[S16] H. Sun, C. Zhong, J.-L. Brédas, *J. Chem. Theory Comput.*, 2015, **11**, 3851–3858.

[S17] F. Neese, *WIREs Comput. Mol. Sci.*, 2012, **2**, 73–78.

[S18] F. Neese, *Mol. Sci.* 2022, **12**, e1606.

[S19] F. Weigend and R. Ahlrichs, *Phys. Chem. Chem. Phys.*, 2005, **7**, 3297–3305.

[S20] E. van Lenthe, J. G. Snijders and E. J. Baerends *J. Chem. Phys.*, 1996, **105**, 6505–6516.

[S21] C. van Wüllen *J. Chem. Phys.*, 1998, **109**, 392–399.

[S22] F. Weigend, *Phys. Chem. Chem. Phys.*, 2006, **8**, 1057–1065.

[S23] F. Neese and F. Wennmohs, A. Hansen and U. Becker, *Chem. Phys.*, 2009, **356**, 98–109.

[S24] B. Helmich-Paris, B. de Souza, F. Neese, and R. Izsák, *J. Chem. Phys.*, 2021, **155**, 104109.

[S25] F. Neese, *J. Chem. Phys.*, 2005, **122**, 034107.

NMR Spectra

^{1}H NMR (600 MHz, $CDCl_3$, rt)

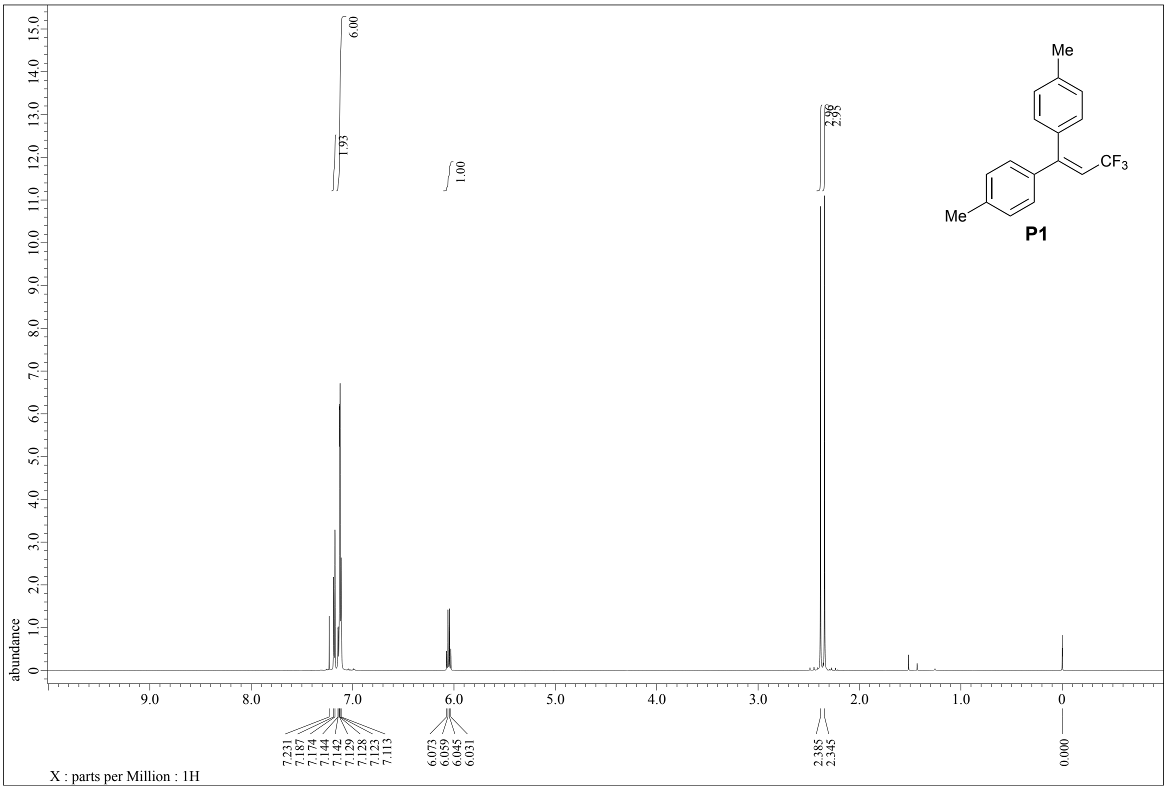

^{13}C NMR (151 MHz, $CDCl_3$, rt)

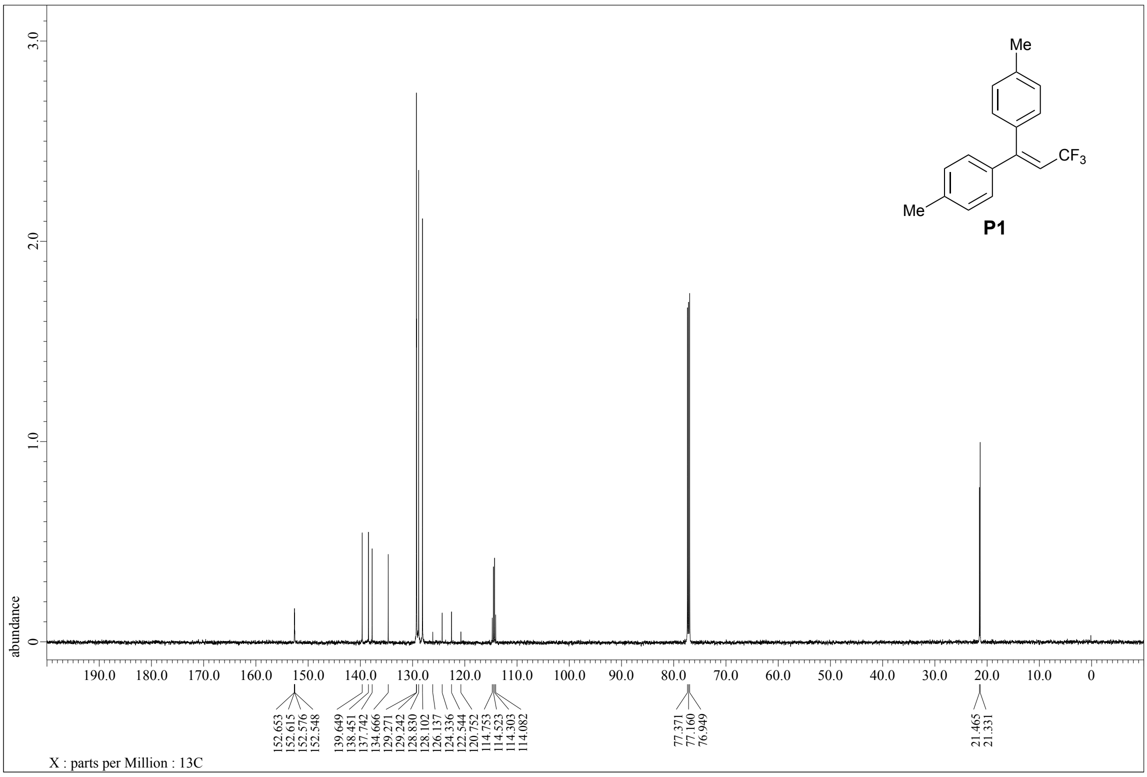

^{19}F NMR (564 MHz, $CDCl_3$, rt)

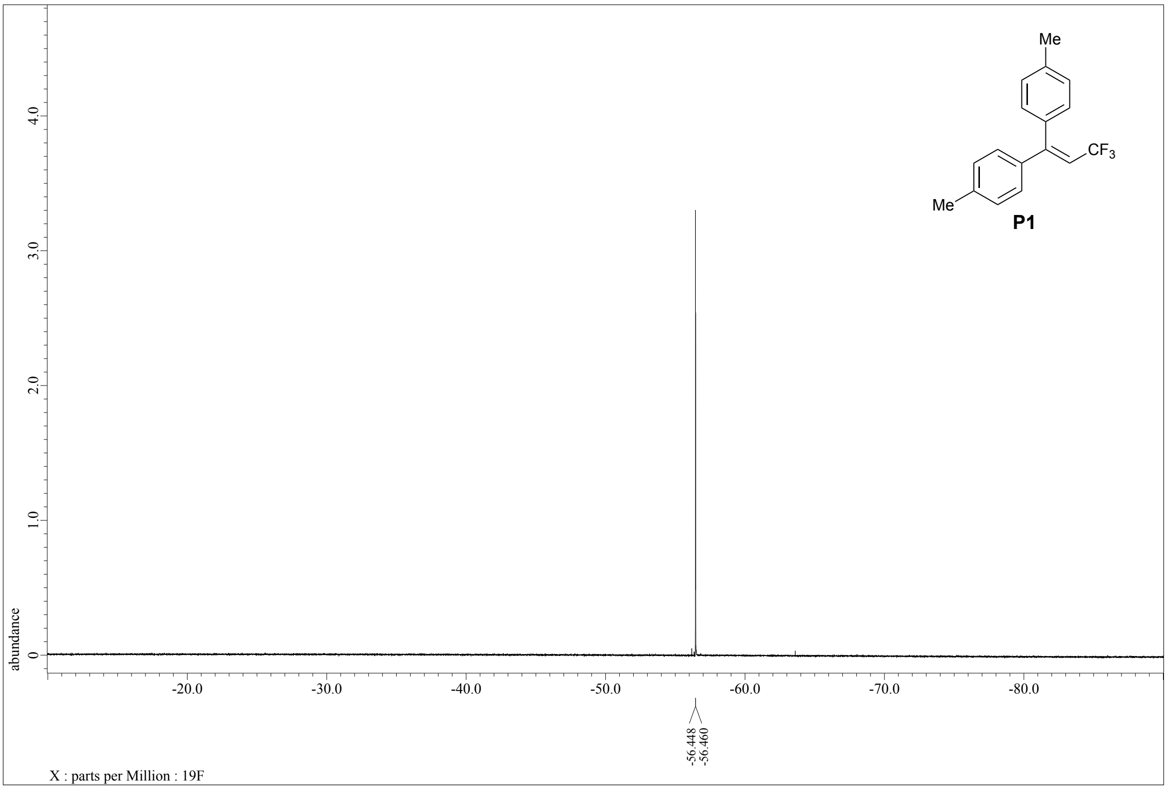

^{1}H NMR (600 MHz, $CDCl_3$, rt)

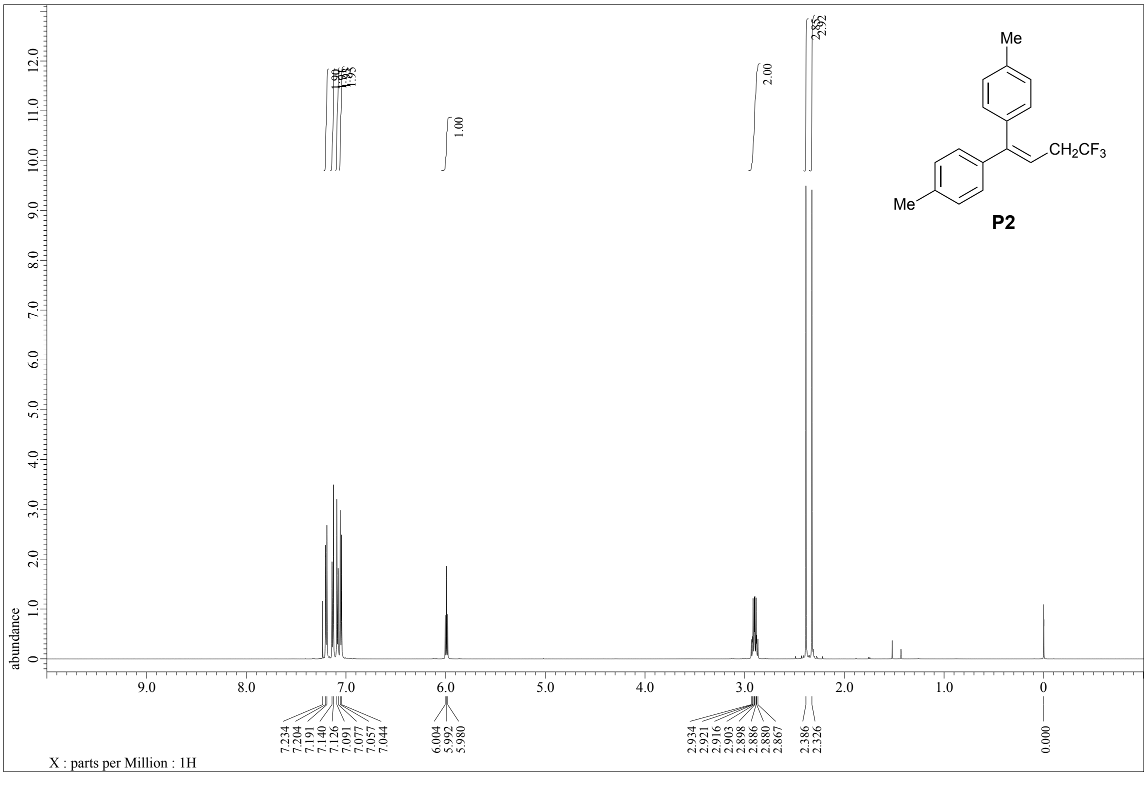

^{13}C NMR (151 MHz, $CDCl_3$, rt)

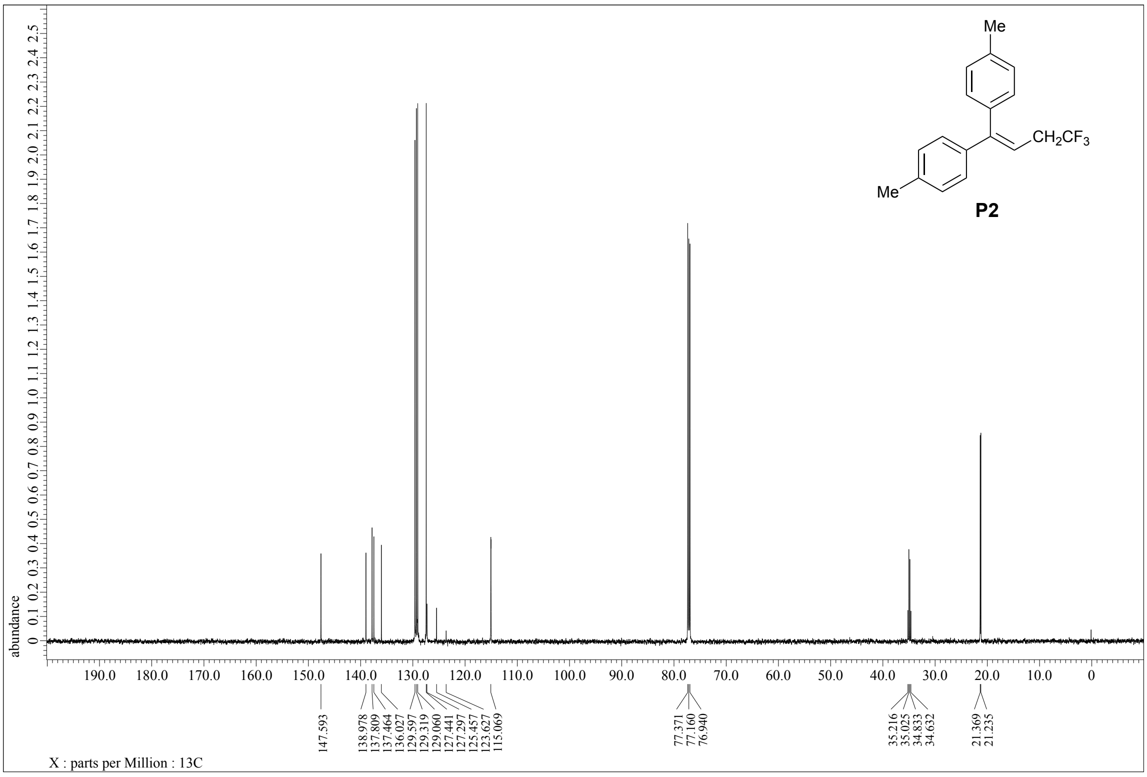

^{19}F NMR (564 MHz, $CDCl_3$, rt)

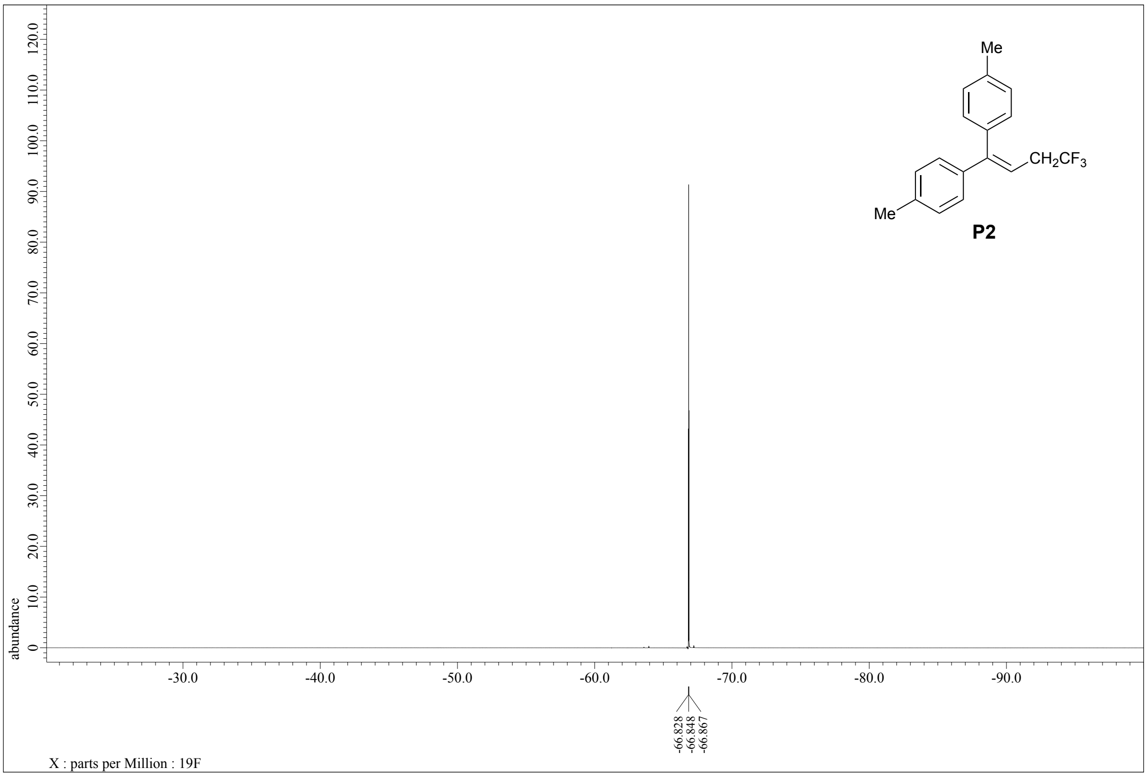

^{1}H NMR (600 MHz, $CDCl_3$, rt)

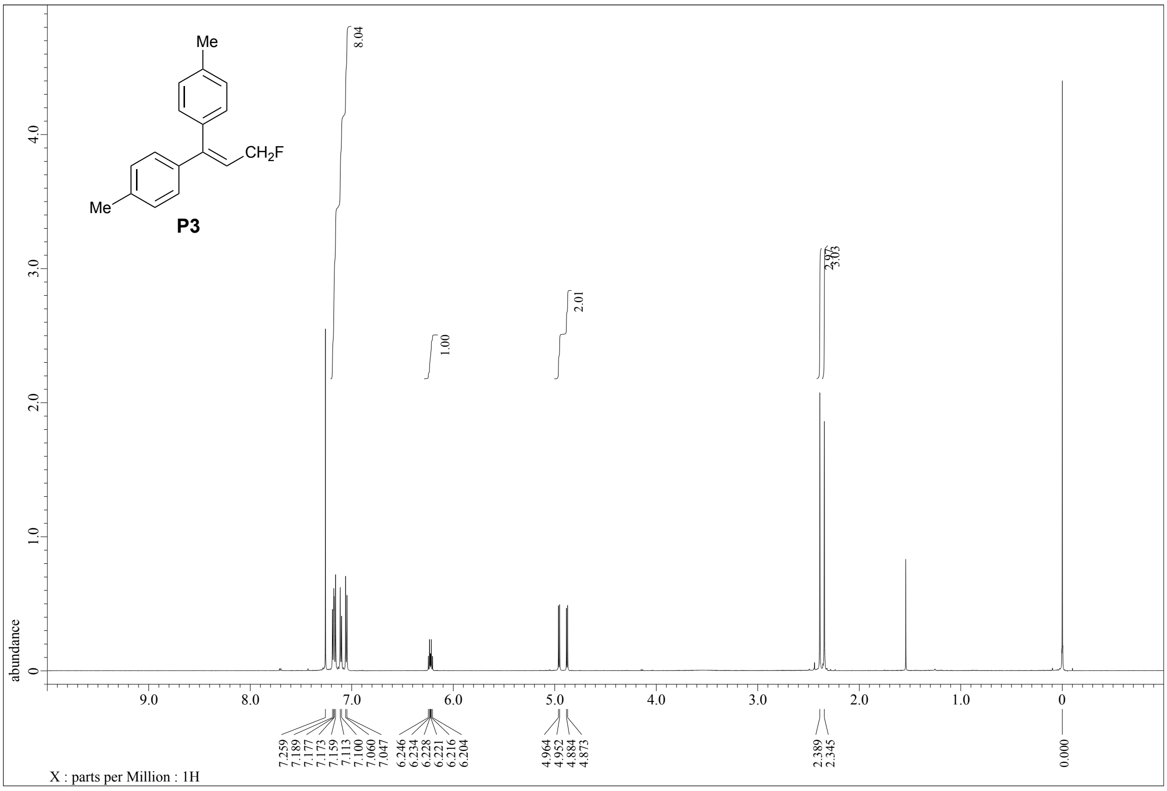

^{13}C NMR (151 MHz, $CDCl_3$, rt)

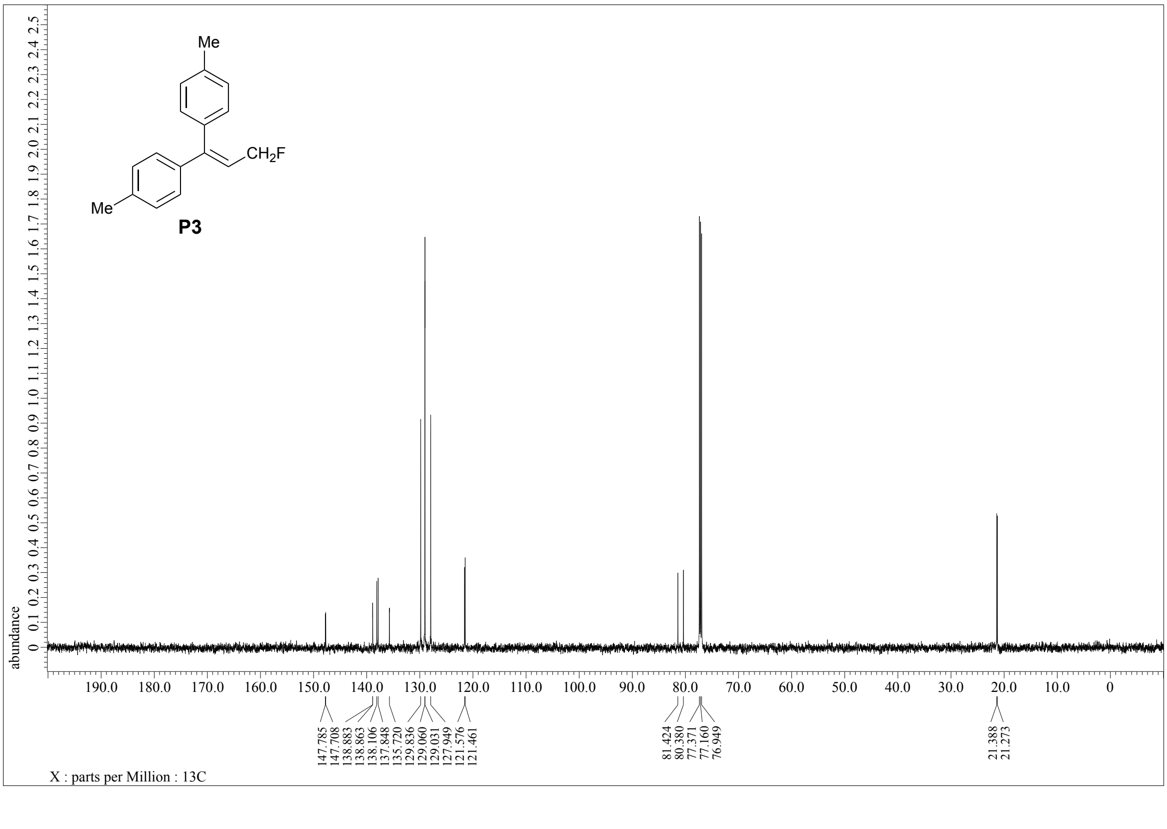

^{19}F NMR (564 MHz, $CDCl_3$, rt)

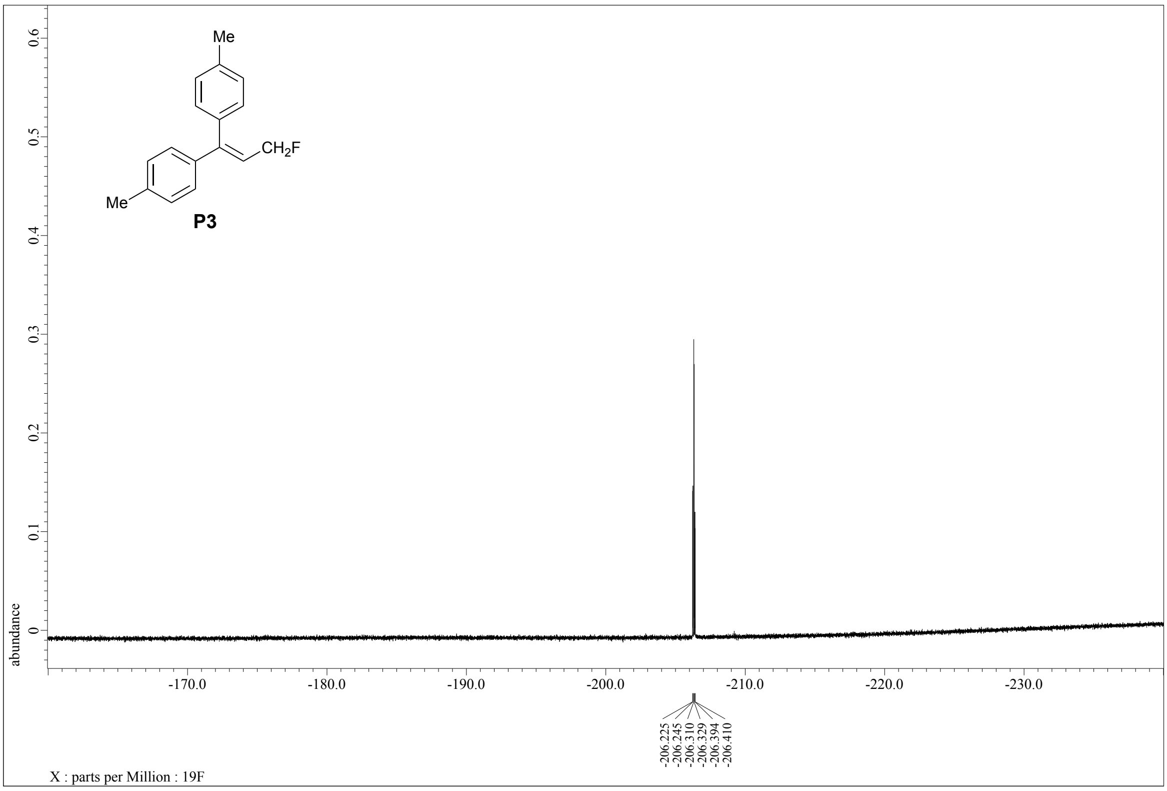

^{1}H NMR (600 MHz, $CDCl_3$, rt)

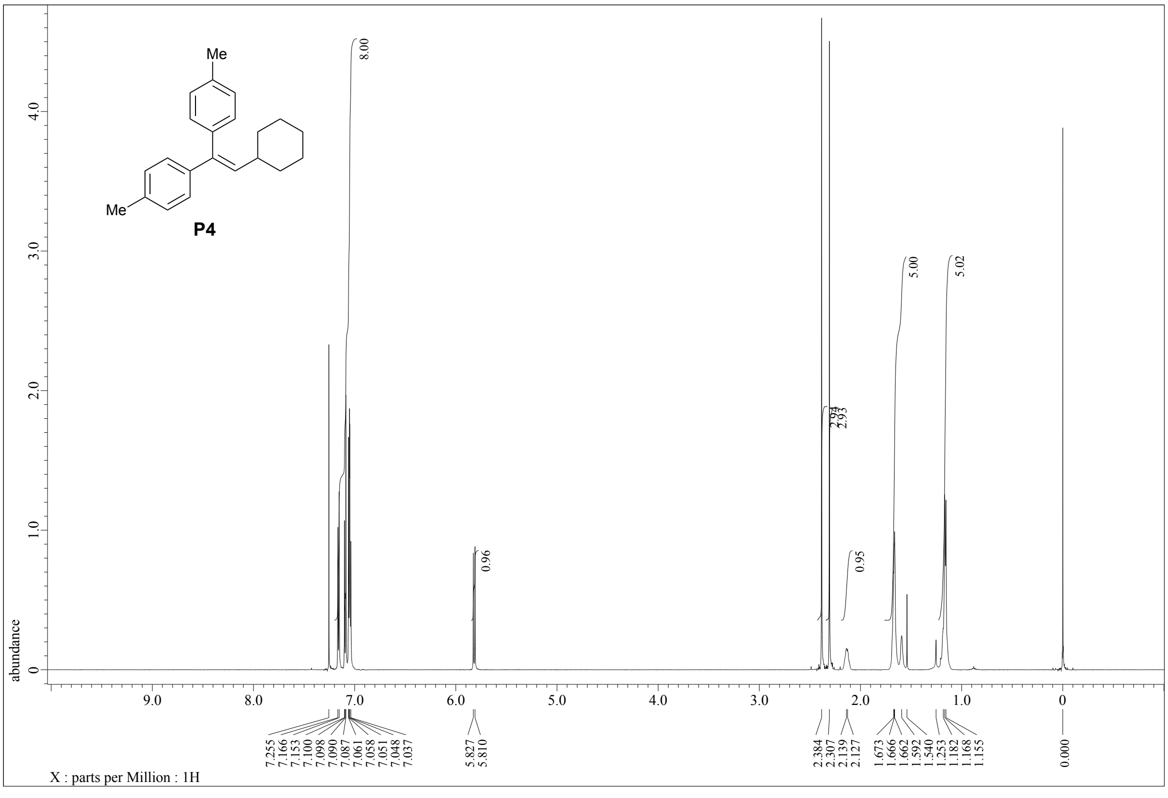

^{13}C NMR (151 MHz, $CDCl_3$, rt)

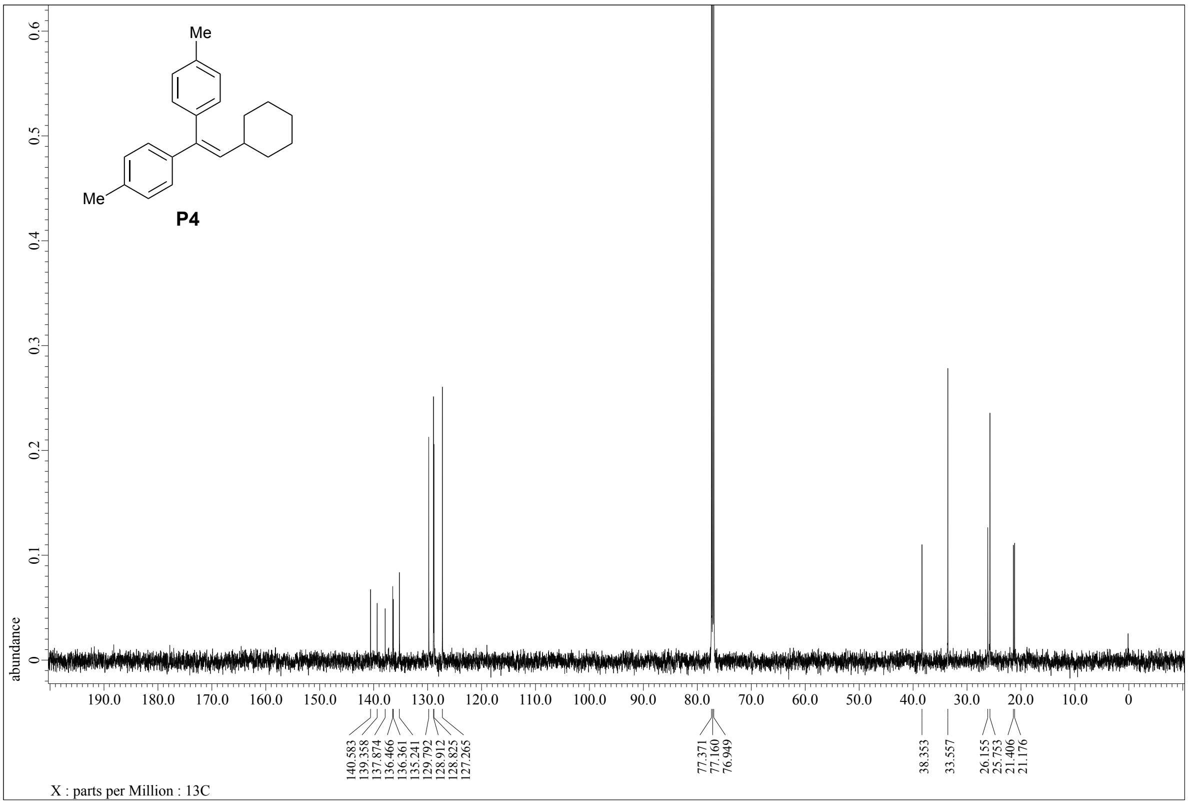

^{1}H NMR (600 MHz, $CDCl_3$, rt)

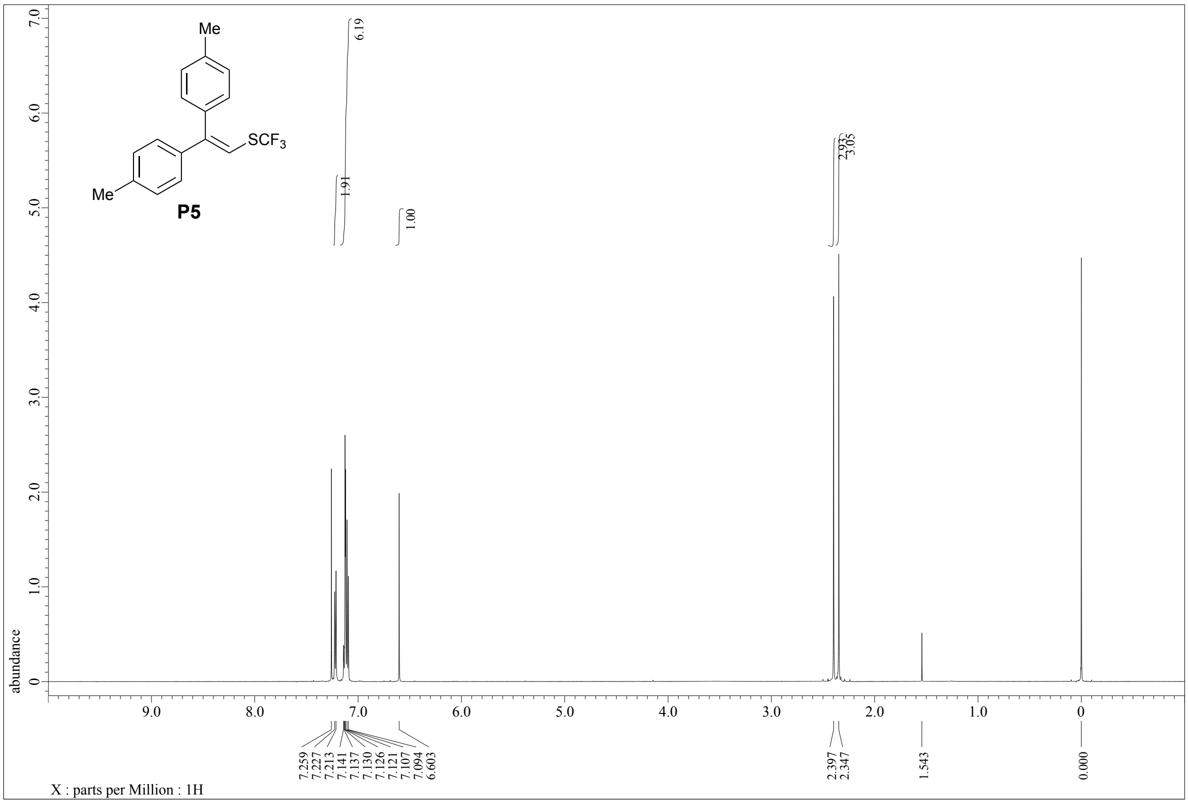

^{13}C NMR (151 MHz, $CDCl_3$, rt)

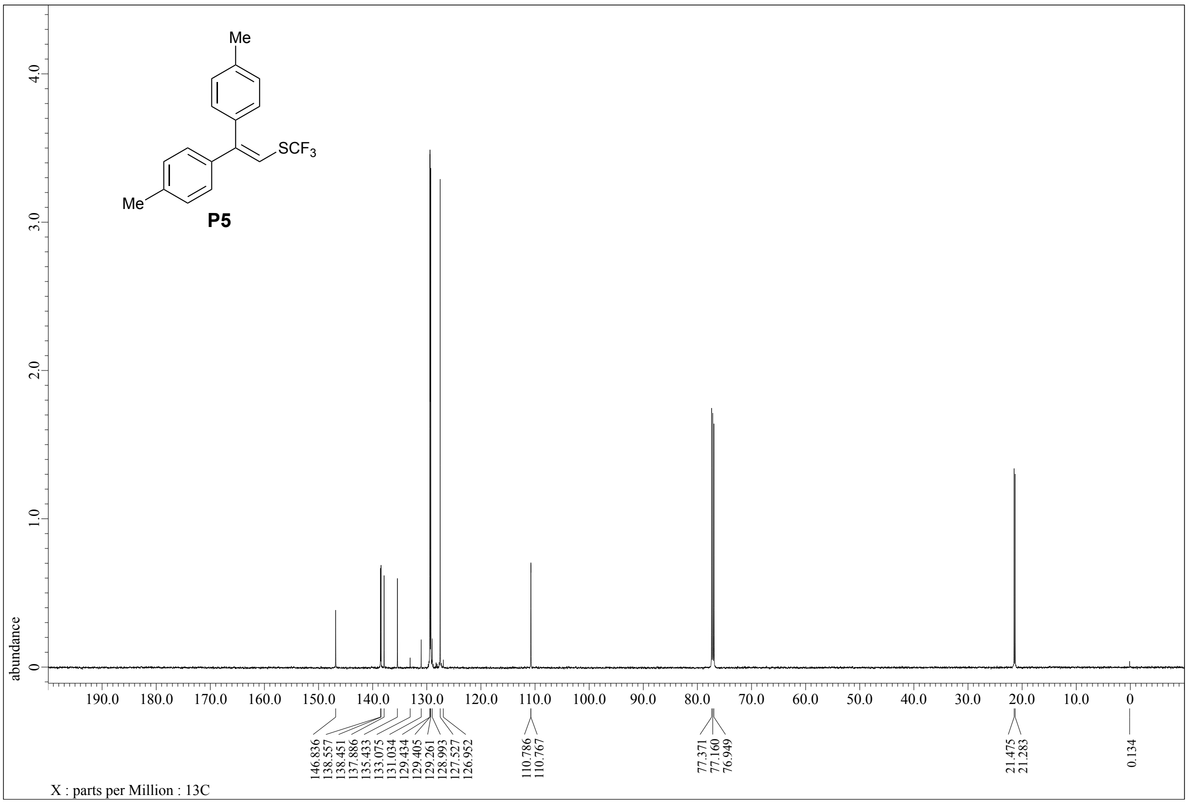

^{19}F NMR (564 MHz, $CDCl_3$, rt)

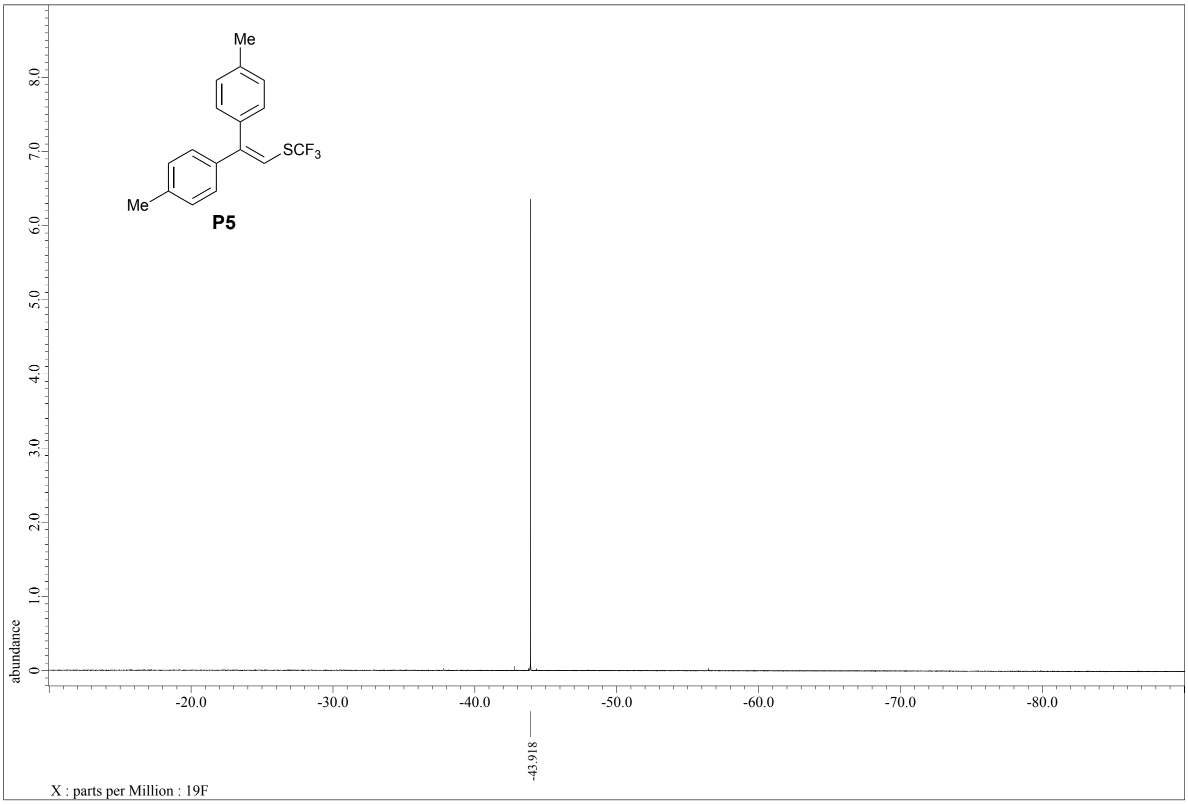

Supplement: SC-017-D5SC06465A-s001 [file SC-017-D5SC06465A-s001.pdf]
